# The Genome and Population Structure of the Spruce Beetle, *Dendroctonus rufipennis* (Kirby, 1837), a Transcontinental Pest of North American Spruce

**DOI:** 10.1111/1755-0998.70209

**Published:** 2026-10-29

**Authors:** Erin O. Campbell, Philip D. Batista, Véronique Lévesque‐Tremblay, Catherine Béliveau, Kathia Bernier, Kateryna Romanenko, Ward B. Strong, Katherine P. Bleiker, Dezene P. W. Huber, Christopher I. Keeling

**Affiliations:** ^1^ Ottawa Plant Lab Canadian Food Inspection Agency Ottawa Ontario Canada; ^2^ Department of Biological Sciences, Faculty of Science University of Alberta Edmonton Alberta Canada; ^3^ British Columbia Ministry of Forests Prince George British Columbia Canada; ^4^ Laurentian Forestry Centre, Canadian Forest Service Natural Resources Canada Québec Québec Canada; ^5^ Département de Biochimie, de Microbiologie et de Bio‐Informatique, Faculté des Sciences et de Génie, Université Laval Québec Québec Canada; ^6^ British Columbia Ministry of Forests Vernon British Columbia Canada; ^7^ Pacific Forestry Centre, Canadian Forest Service Natural Resources Canada Victoria British Columbia Canada; ^8^ Faculty of Environment University of Northern British Columbia Prince George British Columbia Canada

**Keywords:** alpha/beta‐hydrolases, carboxylesterase, co‐sequencing, cytochrome P450, karyotype, pheromone biosynthesis, population structure, speciation, symbionts

## Abstract

The spruce bark beetle *Dendroctonus rufipennis* (Family: Curculionidae; Subfamily Scolytinae) is a major forest insect pest throughout the transcontinental range of spruce species (*Picea* spp.) in North America. Here, we present an annotated, near chromosome‐level, genome assembly of 271 Mb, with 86% of the assembly contained in 16 pseudochromosomes and containing 17,778 predicted gene models. We also annotated 13 protein‐coding genes, 2 rRNAs, and 22 tRNAs in its 19,637 bp mitochondrial genome. In addition, we present the annotated complete nuclear and mitochondrial genomes of an associated endoparasitic nematode, *Contortylenchus* sp. T'it'q'et (Order: Tylenchida; Family: Allantonematidae). Its nuclear genome assembly was 76.2 Mb, with 99.8% of the assembly contained in nine pseudochromosomes and containing 12,208 predicted gene models. We also annotated 12 protein‐coding genes, 2 rRNAs, and 22 tRNAs in its extremely AT‐rich, 15,509 bp mitochondrial genome. Population genomic analyses revealed broad consistencies with past microsatellite work, supporting the hypothesis of multiple range expansions from glacial refugia. Our analyses also identified alternate modes of population diversification which suggest that both neutral and non‐neutral processes may be shaping current population genetic structure across the spruce beetle range.

## 1 Introduction

At least 21 species of bark beetles in the genus *Dendroctonus* (Coleoptera: Curculionidae) infest conifers, with all but two native to North America. The genus likely contains some cryptic species. *Dendroctonus* beetles exhibit individual or mass attack behaviour, acting as early successional saprophages, parasites, and predators that colonize the lower or mid‐bole and roots of trees (Six and Bracewell 2015). Species which mass attack the main bole can kill large diameter mature trees during outbreaks and several particularly eruptive species are notable tree killers. The increasing magnitude and severity of outbreaks of tree‐killing *Dendroctonus* spp., especially in western Canada and the western United States, have raised concern due to effects on forest structure and composition, as well as ecological and socioeconomic impacts, including reduced fibre supply for industry, changes in hydrological cycles, and altered carbon sequestration dynamics (Hicke et al. 2016; Kurz et al. 2008; Meddens et al. 2012). Although bark beetle outbreaks are a natural part of coniferous forests, evidence indicates that anthropogenic effects—primarily climate change and forest management practices—have pushed recent outbreaks beyond historic norms in terms of scale, severity, and impacts (Bentz et al. 2010; Taylor and Carroll 2004; Weed et al. 2013). Genetic and genomic resources for *Dendroctonus* can help inform forest management decisions by addressing questions related to species identification, physiology, phylogeography, population genetic and spatial community structure, adaptive variation, and species interactions, (Cullingham et al. 2018).

The North American spruce beetle, *Dendroctonus rufipennis* (Kirby, 1837) (Coleoptera: Curculionidae: Scolytinae) is considered the most impactful disturbance agent in spruce (*Picea* spp.) forests in Canada and the United States. It plays an important ecological role promoting forest decomposition, succession, and biodiversity (Allen et al. 2006; DeRose and Long 2010; Veblen et al. 1991; Werner et al. 2006). Large diameter recently downed trees or standing trees with weakened defences are preferentially colonized; however, populations can build up in pulses of ideal breeding material, for example, recent windthrow, and then attack and kill more vigorous hosts once preferred host material is exhausted (Bleiker and Safranyik 2021). Sizeable outbreaks have occurred roughly every ten years over the last century in western North America (Bleiker 2021). Warmer temperatures, increased drought, and more frequent extreme weather events attributed to climate change may exacerbate spruce beetle outbreak risk (Bentz et al. 2010; Bleiker and Safranyik 2021).

In contrast to the two notorious pine killers in the genus, mountain pine beetle ( *Dendroctonus ponderosae* Hopkins) and southern pine beetle ( *Dendroctonus frontalis* Zimmermann), whose ranges are limited by cold temperatures, the range of spruce beetle is limited by the occurrence of its *Picea* hosts. Thus, spruce beetle has an extensive geographic distribution that stretches across Canada's boreal and sub‐boreal forests from Alaska in the northwest to Newfoundland in the northeast, through the Great Lakes region, protruding south along the coastal and Rocky Mountains in the west and the Appalachian Mountains in the east (Figure 1). Six spruce species and their natural hybrids occur and serve as potential hosts; exotic spruce species such as Norway spruce have the potential to be attacked (Bleiker and Safranyik 2021; Isitt et al. 2023). The ranges of the different host species vary in size as well as continuity and degree of overlap with each other (Figure 1). Dispersing spruce beetles select a suitable host based on primary attraction and bore under the bark to the phloem where galleries are constructed and brood develop. Attacking beetles carry a myriad of organisms into trees, including bacteria, viruses, yeasts, and filamentous fungi, mites, and nematodes (Cardoza et al. 2008; Davis 2015; Kanzaki et al. 2008; McCambridge and Knight 1972; Pineda‐Mendoza et al. 2024). Aggregation and anti‐aggregation pheromones are released to attract conspecifics and regulate attack density during host colonization. Spruce beetle populations across the range emit and respond to a variety of pheromone blends (Isitt et al. 2018; Pureswaran et al. 2024).

**FIGURE 1:**
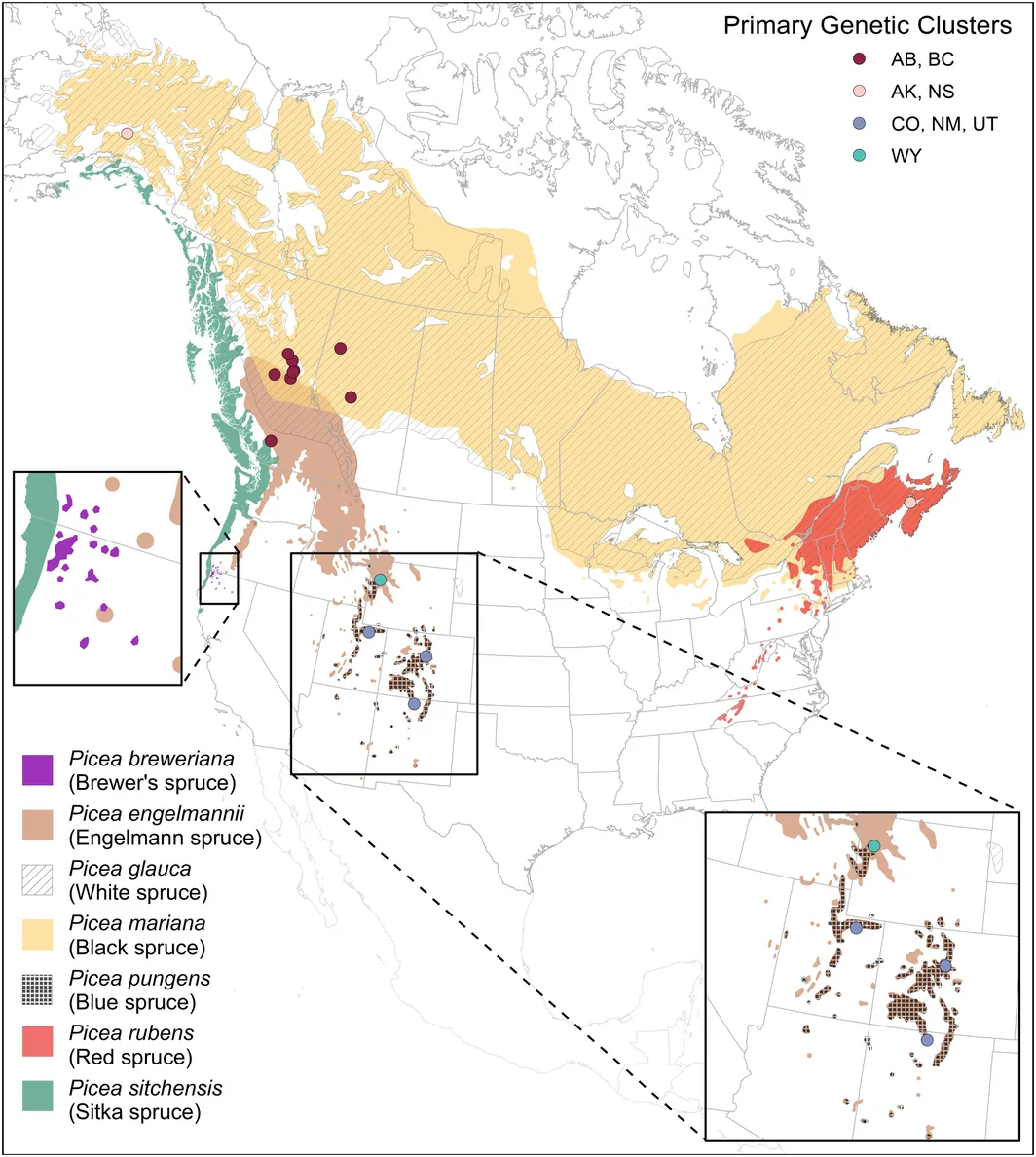
Ranges of North America spruce species and sampling locations. Map showing the ranges of the seven spruce species (*Picea* spp.) native to North America. Map insets show the local ranges of *P. breweriana* and *P. pungens* . Small circles indicate beetle sampling locations, coloured based on the primary genetic clusters determined in this study.

While there is the potential for gene flow from New Mexico to Alaska to Nova Scotia, the distribution and availability of the different *Picea* species may lead to geographic, behavioural, phenological, or other reproductive barriers in neighbouring populations. The most extensive population genetic study on spruce beetle across its range was completed by Maroja et al. (2007) using mitochondrial sequences and nine microsatellite loci. They found evidence of two northern haplotype groups extending from Alaska to Maine and Newfoundland, and a Rocky Mountain clade extending from British Columbia and Washington to Arizona. The Rocky Mountain clade consisted of two subclades—a southern subclade including Arizona and Utah populations, and a subclade including the remaining northern populations. The authors noted that the northern clades were associated with *P. glauca* while the Rocky Mountain clade was associated with *Picea engelmannii* and that the populations using the two hosts likely diverged about 1.7 mya. The population structure indicated expansion from three or more refugia following the most recent glaciation, somewhat analogous to population patterns seen in the closely related mountain pine beetle (Dowle et al. 2017).

There are currently genome assemblies available for 13 species from six genera within Scolytinae. Near chromosome‐level genome assemblies include: *D. frontalis* (Copeland et al. 2024), *D. ponderosae* (Keeling et al. 2022), *D. valens* (Liu et al. 2022), *Euwallacea fornicatus* (Eichhoff) and *E. similis* (Ferrari) (Bickerstaff et al. 2024), *Hypothenemus hampei* (Ferrari) (Navarro‐Escalante et al. 2021), *Ips nitidus* Eggers (Wang et al. 2023), *I. sexdentatus* (NCBI accession CDRJTJ01), *I. typographus* L. (Powell et al. 2021), and *Taphrorychus bicolor* (Herbst) (Telfer et al. 2024). *Dendroctonus* species have at least eight different karyotypes, ranging from 5 AA + neo‐XY to 14 AA + Xy_p_, due to multiple fusion events. The 14 AA + Xy_p_ karyotype, which includes *D. rufipennis* , is believed to be ancestral (Zúñiga, Cisneros, et al. 2002; Zúñiga, Salinas‐Moreno, et al. 2002). Five species contain neo‐XY sex chromosomes due to fusion of one or more autosomes with the sex chromosomes. Our objectives were (1) to sequence the spruce beetle genome and compare it with the genomes of other Scolytinae, particularly its well‐studied congener, the mountain pine beetle (11 AA + neo‐XY karyotype); and (2) to explore population structure and indications of differential selective pressures and geographical isolation, leading to co‐evolutionary mosaics (Thompson 2005) or even speciation (Dowle et al. 2017), across this insect's massive geographic and diverse host ranges. We also manually annotated the cytochromes P450 and carboxyl/cholinesterases. Members of these two large gene families are important not only for endocrine function, but also in xenobiotic detoxification, pesticide resistance, and pheromone biosynthesis (Cruse et al. 2023; Dermauw et al. 2020; Nauen et al. 2022). They are critical in the adaptation of bark beetles to different host species, for example in pheromone‐mediated aggregation behaviour, host choice through olfaction, and host defence detoxification (Blomquist et al. 2021).

## 2 Materials and Methods

### 2.1 Sources of Beetles

Spruce beetles were collected from infested trees near Duffey Lake, Elkford, and Prince George, BC, Canada between 2017 and 2021 for the different uses as described in Supplementary Materials and Methods. Voucher specimens from the population genetics collections from 14 sites in North America (Table S1 in S21) were deposited in the Spencer Entomological Collection at the University of British Columbia, Vancouver, BC, Canada (S22).

### 2.2 DNA Extractions, Genome Sequencing Library Preparation and Sequencing

Genomic DNA for sequencing was extracted using the Blood and Cell Culture DNA Mini Kit (QIAGEN) or a protocol for single insect samples by 10× Genomics (2018) and then Oxford Nanopore, Dovetail Chicago, Hi‐C, and 10× Chromium libraries were prepared and sequenced using PromethION or Illumina technologies by our service providers as described in Supplementary Materials and Methods S21.

### 2.3 Genome Assembly

We assembled the genome in multiple steps (Figure S1). Nanopore reads were filtered and cleaned with *NanoFilt* (v2.7.1, De Coster et al. 2018) to trim 30 bp from the end of each read and remove reads less than 1000 bp or with an average quality score of less than 9. We then removed reads that had significant *BLASTn* (blast+ v2.12.0) matches to the NCBI UniVec database (v2017‐03‐20). The resulting reads were assembled using *flye* (v2.9‐b1778, Kolmogorov et al. 2019, 2020) in metagenome mode. This assembly was then polished with two rounds of *Pilon* (v1.24, Walker et al. 2014) using the Illumina reads from male 10× Chromium sequencing data prepared using *Long Ranger* (v2.2.2, 10× Genomics) and mapped to the assembly with *bwa‐mem* (v0.7.17‐r1188, Li 2013).

The assembly was then scaffolded using *ARCS* (v1.2.1, Yeo et al. 2018) and all three 10× Chromium read sets. The assembly was further scaffolded with *SALSA2* (v2021/06/21, Ghurye et al. 2017) and the combined Chicago and Hi‐C data. The scaffolding of this assembly was examined and manually corrected and improved using *Juicer* (v1.6, Durand et al. 2016) *3D‐DNA* (v201008, Dudchenko et al. 2017) and *Juicebox* (v2.15, Robinson et al. 2018) assembly tools and then further corrected with *HiC‐Hiker* (v1.0.0, Nakabayashi and Morishita 2020). We filled gaps less than 110 bp with *ABySS‐Sealer* (v2.2.5, Paulino et al. 2015) with the Illumina reads from the male 10× Chromium dataset and a sweep of *k* values (40, 50, 60, 70, 80, 90, 100, 110, and 120). We used *PhylOligo* (v1.0, Mallet et al. 2017), *BLASTn* searches against the NCBI nt Release 251 database, and *FCS‐GX* (v0.5.5‐0‐g0bda491, Astashyn et al. 2024) to identify non‐insect scaffolds.

### 2.4 Nuclear Genome Size Estimation

Genome size was estimated by *GenomeScope2.0* (Ranallo‐Benavidez et al. 2020) after *k*‐mer counting with *FastK* (v1.2, Myers Jr. 2026) of the female 10× Chromium Illumina reads with *k*‐mer values of 17, 19, 21, 23, 25, 27, 29, and 31. We also estimated genome size by flow cytometry as described in Gagalova et al. (2022) using three individuals of each sex and *Drosophila melanogaster* as the external reference.

### 2.5 Sex Chromosome Identification

Spruce beetle has a 14 AA + Xy_p_ karyotype and the homogametic sex is female. We predicted the scaffolds representing the sex chromosomes using *DifCover* (v3.0.1, Smith et al. 2018) and the differential coverage of the Illumina reads extracted with Longranger (v2.2.2, 10× Genomics) from the male and female 10× Chromium sequencing datasets. To confirm identification, like done for *D. ponderosae* (Chiu et al. 2018), primer pairs unique to the putative X and Y chromosomes were designed and tested by PCR with genomic DNA from 16 adult beetles of each sex (see Supplementary Materials and Methods S21). To ensure robustness of the primers, these beetles were collected in the summer of 2021 north of Elkford, BC, Canada at 50.21493° N, 114.97922° W, elevation 1400 m, over 500 km from the population of beetles used for genome sequencing.

### 2.6 Shared Synteny and Karyotypes With Other *Dendroctonus* sp.

We compared the spruce beetle assembly to other publicly available chromosome‐level *Dendroctonus* genome assemblies, *D. ponderosae* (Keeling et al. 2022), *D. frontalis* (Copeland et al. 2024), and *D. valens* (Liu et al. 2022) with *ntSynt* (v1.0.8, assuming maximum 20% sequence divergence, Coombe et al. 2024) and *ntSynt‐viz* (v1.0.7, Coombe et al. 2025) to identify orthologous regions and differences in relative chromosomal organization. Analyses were limited to scaffolds over 500 kb, representing each species' pseudochromosomes. We estimated species divergence using *mash* (v2.3, Ondov et al. 2016).

### 2.7 Gene Prediction and Annotation

We used *RepeatModeler* (v2.0.3) and *RepeatMasker* (v4.1.3) and their dependencies within the Dfam TE Tools Container (v1.6, Dfam Consortium 2022) to identify and mask repetitive regions in the assembly. We annotated the masked assembly using *BRAKER2* (Easter 2021 version, Barnett et al. 2011; Bruna et al. 2020, 2021; Buchfink et al. 2015; Gotoh 2008; Hoff et al. 2016, 2019; Iwata and Gotoh 2012; Li et al. 2009; Lomsadze et al. 2005, 2014; Stanke et al. 2006, 2008) with the NCBI Invertebrate RefSeq Protein Release 214 (Goldfarb et al. 2025) and the odb10_arthropoda database (v100, Kriventseva et al. 2019) as protein evidence. We used *TSEBRA* (v1.0.3, Gabriel et al. 2021) to edit the gene predictions. Functional annotations were made based on *BLASTp‐fast* similarity to the full NCBI RefSeq Protein Release 214. We assessed the completeness of predicted transcripts, and the genome assembly, with Benchmarking sets of Universal Single‐Copy Orthologs (*BUSCO* v6.0.0 and the coleoptera_odb12, 2025‐07‐01, *n* = 3729 database, Simao et al. 2015). We removed additional non‐insect scaffolds after annotation, and thus we reidentified repetitive regions in the assembly with *RepeatModeler* (v2.0.7), *RepeatMasker* (v4.2), and their dependencies within the *Dfam TE Tools Container* (v1.93, Dfam Consortium 2022).

### 2.8 Annotation of Cytochromes P450 and Carboxyl/Cholinesterases (CCEs) Gene Families

Cytochrome P450 and carboxyl/cholinesterase (CCE) gene families were manually curated using *Apollo* (v2.6.4, Dunn et al. 2019), preexisting bark beetle proteins, and the ESTHER database (downloaded 2025‐02‐20, Chatonnet et al. 2023). Alignments were performed using *MUSCLE* (v3.0, Edgar 2004) and *mafft* (v7.526, Katoh and Standley 2013), and phylogenies were prepared using *FastTree2* (v2.1.10, Price et al. 2010) and visualized with *FigTree* (v1.4.4, Rambaut 2018) (see Supplementary Materials and Method S21).

### 2.9 Mitochondrial Genome Assembly and Annotation

Nanopore reads were mapped to the published *D. rufipennis* mitochondrial genome with *minimap2* (v2.28, Li 2021) and then assembled using *flye* (v2.9.6‐b1802, Kolmogorov et al. 2019, 2020). The resulting scaffolds were polished with *Pilon* (v1.24, Walker et al. 2014), manually corrected, and then annotations were curated using *MITOS2* (v999, Donath et al. 2019) and *MitoZ* annotate (v3.6, G. Meng et al. 2019). A detailed description is provided in Supplementary Materials and Methods S21.

### 2.10 Identifying Co‐Sequenced Organisms and Nematode Genome Analyses

We used a multi‐step process of detecting and identifying the contaminant sequences with *PhylOligo* (v1.0, Mallet et al. 2017) and *FCS‐GX* (v0.5.5‐0‐g0bda491, Astashyn et al. 2024). We then annotated and assessed the nematode genome in a similar way to the beetle genome. The nematode contaminants were located taxonomically using a combination of *18S* gene sequences and a species phylogeny with *wASTRAL* (hybrid mode, *ASTER* v1.23.3.7, Zhang and Mirarab 2022). Each step is described fully in Supplementary Materials and Methods S21.

### 2.11 Population Genetic Studies

#### 2.11.1 Sequencing and Data Processing for Population Genetic Analysis

DNA extracts were prepared for double digest restriction site associated DNA sequencing broadly following the procedure of Peterson et al. (2012) using the restriction enzymes MspI and PstI, and single‐end sequencing was performed on an Illumina NextSeq 500 at the Molecular Biology Services Unit at the University of Alberta, as described in Keeling et al. (2022).

The *Stacks* 2 (v2.61, Catchen et al. 2011; Rochette et al. 2019) module *process_radtags* was used to demultiplex raw ddRAD sequences, after which remnant adapters and low‐quality reads were removed using *fastp* (v0.19.5, Chen et al. 2018). Cleaned sequences were quality checked in *FastQC* (v0.12.1, Andrews 2019) and then aligned to the *D. rufipennis* genome (described above) using *bwa‐mem* (v0.7.17‐r1188, Li 2013). The resulting reference‐aligned .bam files were sorted using *Samtools* (v1.6, Li et al. 2009), and then the *Stacks* 2 *ref_map.pl* script was used to call SNPs for each sample using default parameters for locus construction. Using the *populations* module of *Stacks* 2, loci were filtered to a maximum global missingness of 20%, a minimum minor allele frequency of 5%, and a single SNP was output from each locus to reduce genomic linkage in the retained markers. We used *VCFtools* (v0.1.16, Danecek et al. 2011) to remove samples and loci with overall mean read depths of less than 10.

#### 2.11.2 Data Processing for Phylogenetic Analysis

To estimate *D. rufipennis* population diversification in North America, we downloaded RADseq data from NCBI for five outgroup *Dendroctonus* taxa previously produced by Godefroid et al. (2019) (Table S7 in S21): two specimens each of *D. pseudotsugae, D* *. simplex* , *D* *. micans* , *D* *. punctatus* , and *D. murrayanae* . Samples were quality checked and aligned to the *D. rufipennis* genome as described above. Then, the *D. rufipennis* and outgroup specimens were processed together in *Stacks* 2 to call SNPs. All parameters were the same as for the *D. rufipennis* ‐only dataset, except that we tolerated up to 50% missingness in the dataset to retain more loci despite greater genomic divergences.

#### 2.11.3 Assessment of Genetic Population Structure

Genetic population clusters were first identified using principal component analysis (PCA) in the *adegenet* package (v2.1.10, Jombart 2008) for *R* (v4.3.3, R Core Team 2025), and then further explored using K‐means clustering and discriminant analysis of principal components (DAPC, Jombart et al. 2010), also implemented in *adegenet*, to resolve hierarchical structuring.

We estimated individual ancestries with *ADMIXTURE* (v1.3.0, Alexander et al. 2009), which implements a maximum likelihood algorithm to infer genetic ancestry from SNP data without the use of prior population assignments. We tested values of K between 1 and 14 and performed 10 cross‐validation replicates for each value of K; the value of K with the lowest cross‐validation error is assumed to represent the optimal number of genetic ancestries. Once primary population clusters were identified, we calculated pairwise population weighted *F*_ST_ (Weir and Cockerham 1984) in *vcftools* for each of the 16 putative chromosomes using a sliding window size of 100,000 bp and a step size of 10,000 bp.

We then used the *R* package *pixy* (v2.2.1, Korunes and Samuk 2021) to estimate nucleotide diversity (π), *F*_ST_ and *d*_xy_ for each of the primary clusters, using a window size of 10,000 bp. pixy uses both variant and invariant sites to produce unbiased estimates of genetic variation. Accordingly, we used the *populations* module in *Stacks 2* to output a vcf file containing all sites (‐‐vcf_all option), and used a locus whitelist to ensure that only loci from our filtered dataset were included in this file. We calculated average π for each population and sampling locality, and average *F*_ST_ and *d*_xy_ for each pairwise comparison. We additionally plotted *F*_ST_ and *d*_xy_ along each of the 16 putative chromosomes for population pairs used in outlier analyses. All population genomic analyses conducted in *R* were plotted with ggplot2 (v3.4.1, Wickham 2016).

#### 2.11.4 Phylogenetic Analyses

The combined *D. rufipennis* and outgroup phylogeny was estimated using the maximum likelihood approach implemented in *IQ‐TREE* 2 (v2.1.4, Minh et al. 2020). We allowed the program to determine the optimal substitution model for the data (Kalyaanamoorthy et al. 2017), conducted 1000 ultra‐fast bootstrap replicates (Hoang et al. 2018), and 1000 replicates of SH‐like approximate likelihood ratio branch testing (Guindon et al. 2010), and output the results as a 50% majority rule consensus tree.

#### 2.11.5 Outlier Analyses

Following sliding window *F*_ST_ analyses, three pairwise population comparisons appeared to exhibit specific chromosomal regions with elevated *F*_ST_ values relative to the rest of the genome: British Columbia and Alberta versus Wyoming; British Columbia and Alberta versus Alaska and Nova Scotia; and Alaska and Nova Scotia versus Wyoming. To investigate these markers in more detail, we created three subsets of the SNP dataset used for population genomic analyses so that each subset contained only individuals from the two populations for each of the three pairwise comparisons. For each of these subsets, we then created a null distribution of putatively neutral SNPs in *Plink* (v1.9, Purcell et al. 2007) and then used *OutFLANK* (v2, Whitlock and Lotterhos 2015) implemented in *R* to identify putative outlier loci for each of these population comparisons against the null distribution.

In *OutFLANK* we created an *F*_ST_ matrix for each of the three data subsets described above using the *MakeDiploidFSTMat* function, and then checked for any loci with low heterozygosity and/or low sample size that may have artificially inflated *F*_ST_ values. To create the null SNP distribution for each pairwise comparison, we used the indep‐pairwise function in *Plink* to prune each and remove markers in apparent linkage disequilibrium. We set our window size to 200,000 bp, our step size to 100 bp, and used an *R* ^2^ threshold of 0.5. Next, with the *OutFLANK* function we then used the pruned datasets to estimate the neutral mean *F*_ST_ and degrees of freedom for subsequent fitting of the data to a chi‐square distribution. We specified two populations for each pairwise comparison, a false discovery rate (qthreshold) of 0.05, and a minimum heterozygosity of 0.1.

Lastly, using the *pOutlierFinderChiSqNoCorr* function in *OutFLANK*, we calculated *p*‐values and *q*‐values for all loci in each of the three pairwise comparisons. We used the inferred degrees of freedom, uncorrected *F*_ST_ values and mean *F*_ST_ estimated from the pruned dataset to correct the outlier analysis for each of the three comparisons and set our false discovery rate to 0.05 and our minimum heterozygosity to 0.1. We then visualized statistically significant outlier SNPs by chromosome and cross‐referenced these SNPs against the genome annotation to obtain putative identities.

## 3 Results

### 3.1 Assembly

We assembled a total of 9.8 million Nanopore reads with an N50 of 8632 bp (longest read 651,229 bp) with *flye*. We then polished the resulting initial assembly (395 Mb, N50 = 1.5 Mb and L50 = 54 in 6749 contigs) with two rounds of *Pilon* and scaffolded it with the 10× Genomics data using *ARCS*. The resulting assembly (395 Mb, N50 = 3.0 Mb and L50 = 33 in 6517 sequences) was further scaffolded using the Chicago and Hi‐C data using *SALSA2* to yield an intermediate assembly of 395 Mb, N50 = 7.9 Mb and L50 = 18 in 6051 sequences. We examined this assembly's Hi‐C contact map in detail with *Juicebox* and corrected obvious inversions, ordering errors, mis‐joints, and Hi‐C supported scaffolding and refined it further using *Hi‐C Hiker*, resulting in an assembly of 395 Mb, N50 = 11.5 Mb and L50 11 in 6067 sequences. We then attempted to fill the gaps between contigs within scaffolds with *ABySS‐Sealer*, resulting in 13% of the gaps being filled. This draft assembly contained 395 Mb, N50 = 11.5 Mb and L50 = 11 in 6067 sequences. However, we observed an elevated assembly size and a Hi‐C contact map that showed 16 beetle scaffolds of decreasing length with mutual contacts followed by nine scaffolds with no significant inter‐chromosomal mapping to the larger scaffolds (Figure S2). We subsequently determined that these nine and several other smaller scaffolds originated from Nematoda and other non‐insect taxa. We identified these sequences using *PhylOligo*, *FCS‐GX* (Astashyn et al. 2024), *BLASTn* searches against NCBI nt, and *BLASTp* searches of the annotations against NCBI nr (Supplementary Materials and Methods S21 and Figure S3). After removing these scaffolds, the final spruce beetle assembly contained 271 Mb, N50 = 15.9 Mb and L50 = 7 in 3571 sequences longer than 1000 bp (Table 1 & Figure 2). Assembly completeness was evaluated by aligning the Illumina short reads from the male 10× Illumina data used for polishing against the final genome assembly, resulting in a mapping rate of 87%. In addition, *BUSCO* analysis (v6.0.0 and the coleoptera_odb12, 2025‐07‐01, *n* = 3729 database) revealed that 98.7% of conserved genes were found in the genome, including 96.5% single‐copy, 2.0% duplicated, 0.2% fragmented and just 1.3% missing genes (Figure S4).

**TABLE 1:** Summary of *Dendroctonus rufipennis* assembly statistics.

Parameter	Value
Genome size predictions	
*k*‐Mer counting (Mb)^a^	281
Flow cytometry (Mb)	361 ± 10
Genome sequencing depth^b^	
Nanopore	184‐fold
10× Illumina	218, 281, and 295‐fold^c^
Chicago Illumina	174‐fold
HiC Illumina	143‐fold
Assembly	
Overall assembly	
Assembly size (Mb)	270.7
GC content (%)	35.7
Repetitive sequences (%)^d^	38.83
BUSCO completeness of assembly (%)^e^	98.5
Scaffolds	
Number of scaffolds	3571
Longest scaffold (Mb)	28.2
N50 scaffold length (Mb)	15.9
L50 scaffold count	7
Contigs	
Number of contigs	4122
Longest contig (Mb)	9.8
N50 contig length (Mb)	1.8
L50 contig count	37
Number of contigs in scaffolds	767
Annotation	
Predicted gene models	17,778
Predicted transcripts	19,867
BUSCO completeness of annotation (%)^e^	94.2

^a^ Maximum genome haploid length from GenomeScope 2.0 when *k* = 31.

^b^ Coverage calculations assume a genome size of 361 Mb from flow cytometry.

^c^ For samples SAMN15325384, SAMN15325870, and SAMN15325942, respectively.

^d^ See Table S4 in S21 for details.

^e^ See Figure S4 for details.

**FIGURE 2:**
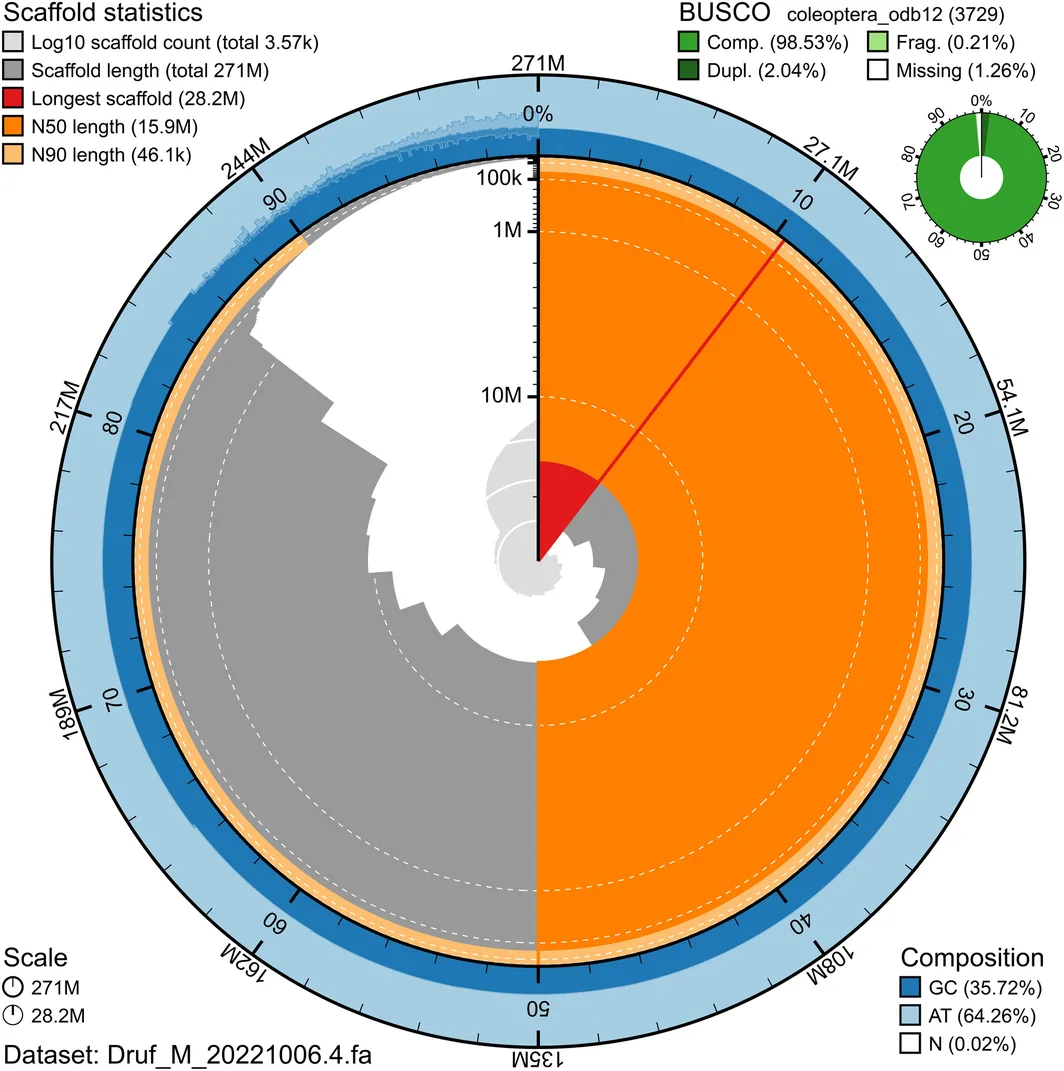
Spruce beetle assembly statistics snail plot. A red segment represents the longest scaffold while all other scaffolds are arranged in size‐order moving clockwise around the plot and drawn in grey starting from the outside of the central plot. Dark and light orange arcs show the N50 and N90 values. The central light grey spiral shows cumulative scaffold count with a white line at each order of magnitude. The dark vs. light blue area around the outside of the plot shows mean, maximum and minimum GC vs. AT content at 0.1% intervals. White regions in this area reflect the proportion of Ns in the assembly. BUSCO scores for the genome assembly are shown in the upper right‐hand corner.

### 3.2 Nuclear Genome Estimation

We used a *k*‐mer based approach to estimate a haploid genome size of 281 Mb (*k* = 31, Figure S5). The female 10× Illumina data was analysed because it originated from one individual beetle and did not show evidence of nematode contamination (via *bwa* mapping to the draft assembly). We also used flow cytometry to estimate genome size based on the amount of DNA contained within the haploid nucleus (C‐value). This estimated haploid genome size of spruce beetle was 0.37 ± 0.01 pg. (mean ± SD) or 361 ± 10 Mb.

### 3.3 Sex Chromosomes

We identified scaffold Druf_M_20221006_9 as the X‐chromosome and scaffold Druf_M_20221006_16 as the Y‐chromosome through differential sequencing coverage (Figure S6). PCR with adult genomic DNA confirmed the Y‐chromosome. The X‐ and Y‐chromosomes were 15.82 and 0.54 Mb long and contained 820 and 25 gene models, respectively. The previously sequenced mountain pine beetle has an 11 AA + neo‐XY karyotype, while the spruce beetle has a 14 AA + Xy_p_ karyotype. These assemblies suggest the MPB neo‐X chromosome is orthologous to spruce beetle scaffolds 1, 9, 11, and 12 (Figure 3).

**FIGURE 3:**
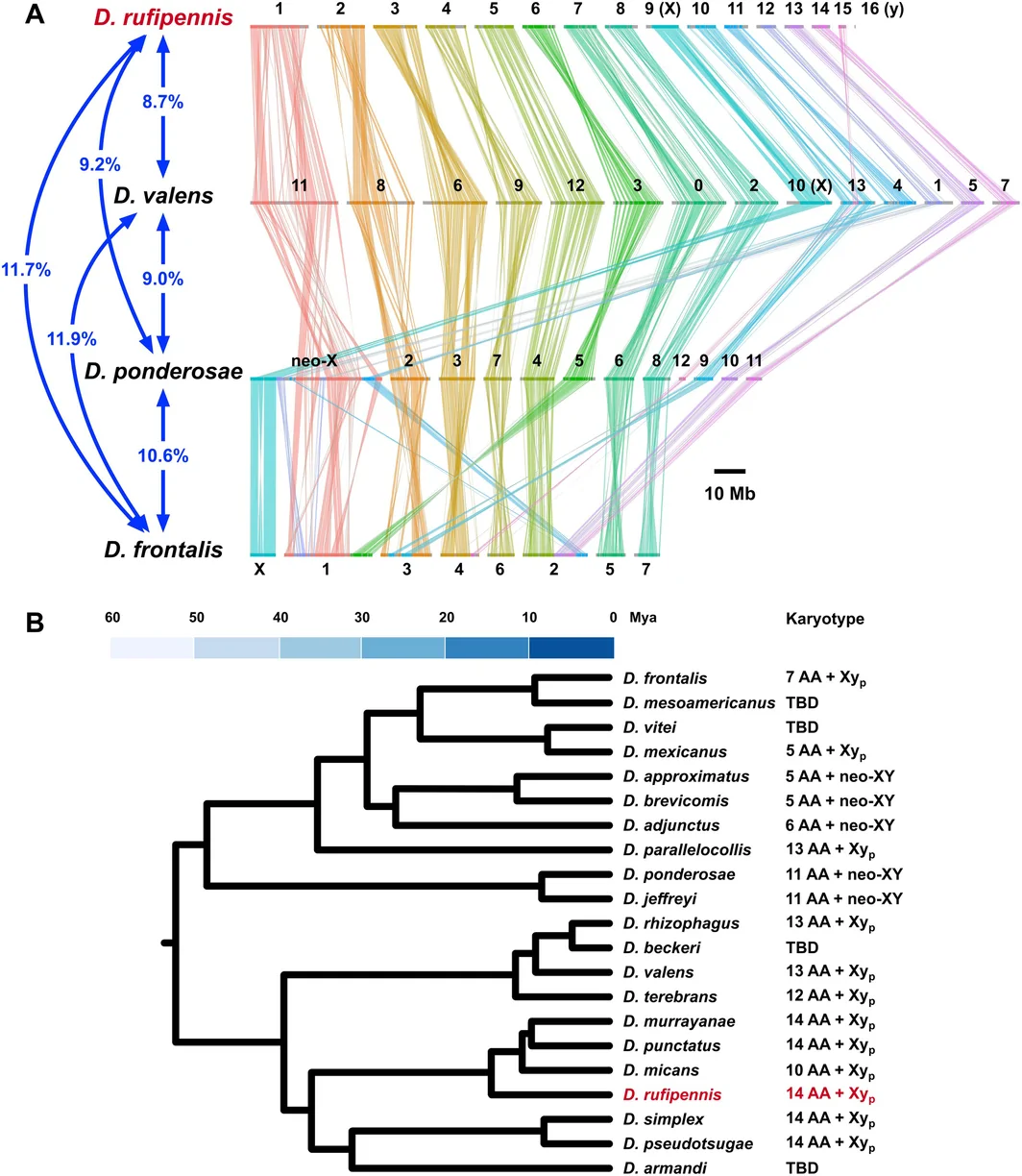
Conserved synteny between spruce beetle, red turpentine beetle, mountain pine beetle, and southern pine beetle genomes and karyotype divergence in *Dendroctonus*. (A) Shared synteny between *D. rufipennis* (this study), *D. valens* (Liu et al. 2022), *D. ponderosae* (Keeling et al. 2022) and *D. frontalis* (Copeland et al. 2024) assemblies. Only the *D. rufipennis* assembly has an identified Y chromosome. This scaffold did not share any synteny with the remaining chromosomes in the other species. Sequence divergence rates between each species are shown in blue on the left, as estimated with *mash* (*p* << 0.0001, Ondov et al. 2016). For greater detail, pair‐wise synteny plots between *D. rufipennis* and the other species are shown in Figure S18. (B) For reference, we show the divergence times for *Dendroctonus* spp. as previously estimated by Ramírez‐Reyes et al. (2023) and corresponding karyotypes as reported by Zúñiga, Cisneros, et al. (2002) and Zúñiga, Salinas‐Moreno, et al. (2002).

### 3.4 Gene Prediction and Annotation

We found 105 Mb of repeat sequences, representing 38.83% of the assembly. Of these, 59% were known repeat elements (Table S2 in S21). We annotated the repeat‐masked genome using *BRAKER2* with the NCBI RefSeq Invertebrate Release 214 and odb10_arthropoda (OrthoDB v10) protein databases. We found 19,867 predicted transcripts from 17,778 predicted gene models. *BUSCO* analysis (v6.0.0 and the coleoptera_odb12, 2025‐07‐01, *n* = 3729 database) of the predicted transcripts (including splice variants) revealed that 96.6% of the conserved genes were found in the annotations, including 82.1% single‐copy, 12.1% duplicated, 2.4% fragmented and 3.4% missing genes (Figure S4). We determined the consensus intron donor/acceptor sites and found that introns ranged between 42 and 27,938 bp (Figure S7).

### 3.5 Cytochromes P450 and Carboxyl/Cholinesterases Gene Families

We annotated 71 cytochromes P450 in the genome assembly, nine from clan 2, 34 from clan 3, 21 from clan 4, and seven from the mitochondrial clan. P450s were distributed throughout the genome except on scaffolds 10 and 16 (y) (Figure 4). Scaffold 1 had disproportionately more P450s, 26 overall, including 11 and 9 from CYP6 and CYP9 families, respectively. All eight CYP9Zs were clustered on scaffold 1 within 90 kb.

**FIGURE 4:**
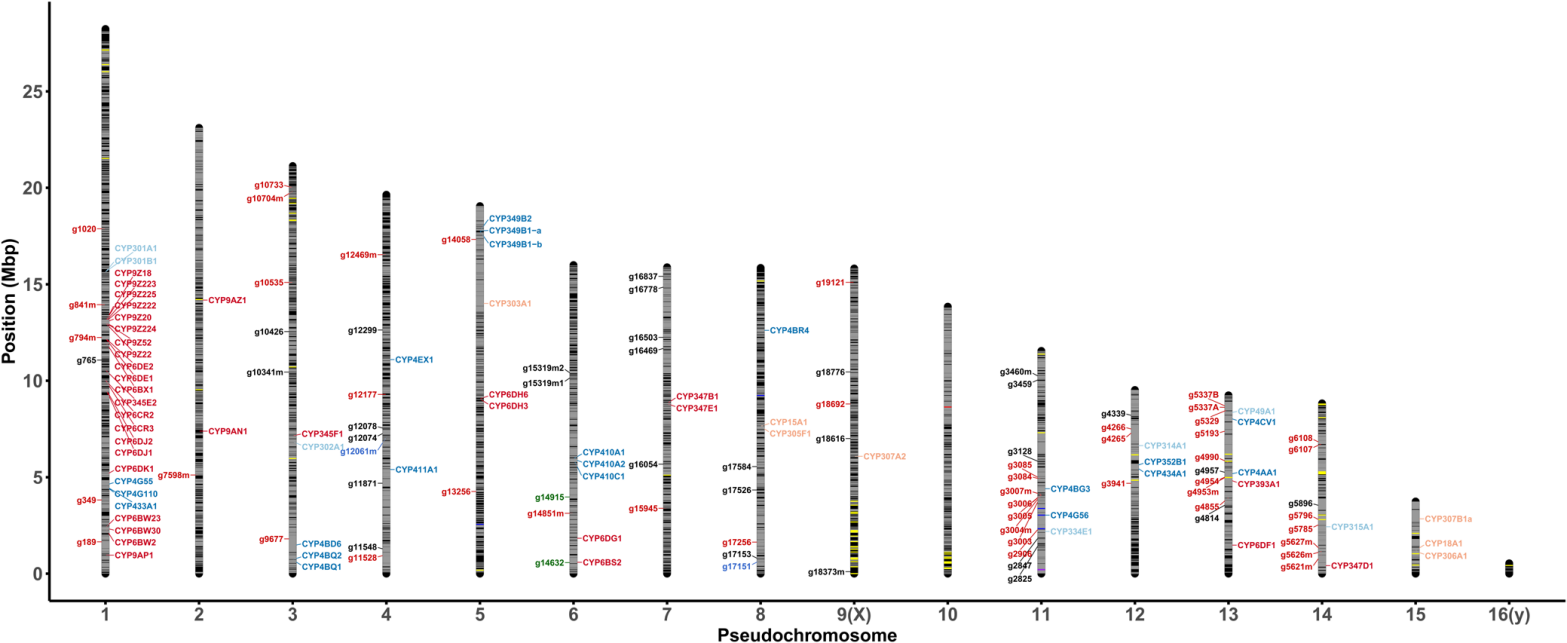
Genome locations of cytochromes P450 and carboxyl/cholinesterases and related genes. The 16 largest scaffolds (pseudochromosomes) are shown to scale. The left side of each scaffold shows the locations of the CCEs and related genes from Block C (red), Block H (blue), Block L (green), and Block X (black). The right side of each scaffold shows the locations of the P450s from Clan 2 (orange), Clan 3 (red), Clan 4 (blue), and the Mitochondrial Clan (pale blue). Within each scaffold, grey bands indicate the locations of all the ddRAD markers. Coloured bands indicate markers deemed to be significant outliers by Outflank: BC‐ABvsWY (77, yellow), BC‐ABvsAK‐NS (1, red), AK‐NSvsWY (5, blue), and both BC‐ABvsAK‐NS and AK‐NSvsWY (2, purple).

We annotated 81 carboxyl/cholinesterases (CCEs) and related genes. They were distributed throughout the genome but were absent from scaffolds 10, 15, and 16 (y) (Figure 4). Scaffolds 11, 12, and 14 had the highest density of CCE gene models, with scaffold 11 containing five paralogues within 18 kb.

### 3.6 Mitochondrial Genome

We confirmed the spruce beetle mitochondrial genome (Meng et al. 2023) using our Nanopore data, which spans the repetitive AT‐rich region. The average coverage was 351‐fold of Nanopore reads after 2% subsampling. The AT content was 74.7%. Although highly similar to that previously reported, we found a larger genome size (19,637 vs. 16,937) due to expansion of the AT‐rich region through a 150 bp repeat between the small rRNA subunit and tRNA‐Ile (gau) totalling 749 bp, and a 40 bp repeat between tRNA‐Ile (gau) and tRNA‐Gln (uug) totalling 1950 bp (Figure S8).

### 3.7 Co‐Sequenced Organisms

We identified and removed 95.7 Mb in 428 scaffolds from the beetle assembly that were not of insect origin. These could be further separated into 411 nematode sequences from the order Tylenchida (17 sequences totalling 76.2 Mb from Allantonematidae, most closely related to *Bradynema listronoti*, and 394 sequences totalling 19.2 Mb mostly from the family Aphelenchoididae); 15 Gammaproteobacteria sequences (14 sequences totalling 226 kb, 89%–97% identical to genomic sequences from *Rouxiella* sp. WC2420 and one sequence of 9.3 kb, 95.5% identical to genomic sequence from *Rahnella bonaserana* strain L46); one Alphaproteobacteria sequence [11 kb, 90.4% identical to genomic sequence from a *Rickettsia* endosymbiont of *Ceutorhynchus obstrictus* (Coleoptera: Curculionidae) isolate 54717]; and one fungus sequence (11 kb, 96.0% identical to mitochondrion sequence from *Yamadazyma tenuis* strain ATCC 10573).

We identified three sequences among the nematode scaffolds that contained *18S* or *28S* ribosomal RNA genes to aid in species identification. Scaffold hicscaffold_000022 (5.9 Mb, one of the 17 sequences from family Allantonematidae) contained both *18S* and *28S* rRNA genes. These were 97% identical over 1721 bp and 98% identical over 756 bp, respectively, to the corresponding *18S* and *28S* sequences from *Contortylenchus* sp. Tomakomai (LC219517) (Allantonematidae) and *Contortylenchus genitalicola* Kyoto (LC744417.1). Scaffold hicscaffold_003047 (6268 bp) was 100% identical over 1699 bp to the *18S* rRNA gene from *Bursaphelenchus rufipennis* (AB368529) (Aphelenchoididae). Scaffold hicscaffold_003829 (4259 bp) was 99% identical over 1710 bp to the *18S* rRNA gene from *Ektaphelenchus obtusus* (AB368532) (Aphelenchoididae). We also located these *18S* rRNA sequences within a phylogeny of sequences from the Nematoda infraorder Tylenchomorpha (Figure S9). Although the taxonomy of both *Bursaphelenchus* and *Ektaphelenchus* genera are incomplete, our results suggest the presence of *B. rufipennis*, *E. obtusus* , or closely related species, and an unknown species of *Contortylenchus* in our beetle samples.

The 17 sequences from the nematode family Allantonematidae appeared to constitute a full genome of the *Contortylenchus* species (Figure S10A) and were examined further, whereas the other remaining nematode sequences did not represent a complete genome (Figure S10B). The *Contortylenchus* sp. genome contained 76.2 Mb, had an N50 of 8.4 Mb in five scaffolds (L50), a GC‐content of 35.84%, and 99.8% of the assembly was contained in nine pseudochromosomes (Table S3 in S21). The SNP densities from the Nanopore data for these pseudochromosomes varied between 0.27 and 0.51 sites/kbp, consistent with the presence of only one species in these scaffolds (c.f., 5.1–6.13 and 0.34 sites/kbp for *Caenorhabditis briggsae* and *Strongyloides stercoralis*, respectively [Koboldt et al. 2010; Liu et al. 2025]). *BUSCO* analysis (v6.0.0 and the nematoda_odb12, 2025‐07‐01, *n* = 596 database) revealed that 97.6% of conserved BUSCO genes were found in the genome, including 93.5% single‐copy, 2.3% duplicated, 1.8% fragmented and 2.3% missing genes (Figure S11). We used these predicted single‐copy conserved genes and those from nematode reference genomes in NCBI to place this species in a species phylogeny of the phylum Nematoda (Figure S12). The species was situated between *Sphaerularia bombi* (Sphaerulariidae), *Deladenus siricidicola* (Sphaerulariidae), and *Bradynema listronoti* (Allantonematidae), consistent with the genus *Contortylenchus* (Allantonematidae).

We identified a total of 11.7 Mb repeat sequences representing 15.41% of the assembly. Among these repeat sequences, 47% were classified as known repeat elements (Table S4 in S21). We then annotated the genome using *BRAKER3* with the NCBI RefSeq Invertebrate Release 231 and metazoa_odb12 (OrthoDB v12) protein databases. We found 13,189 predicted transcripts from 12,208 predicted gene models. *BUSCO* analysis (v6.0.0 and the nematoda_odb12, 2025‐07‐01, *n* = 596 database) of the predicted transcripts (including splice variants) revealed that 97.5% of the conserved genes were found in the annotations, including 85.7% single‐copy, 9.1% duplicated, 2.7% fragmented and just 2.6% missing BUSCO genes (Figure S11).

In addition to the nematode nuclear genome, we identified three putative full‐length nematode mitochondrial genome sequences within the nematode sequences: hicscaffold_001276 (18.8 kb, < 85% identical to any sequenced nematode), hicscaffold_001451, putative mtDNA genome of *Bursaphelenchus rufipennis* (16.8 kb, 97% identical to its COI on NCBI), and hicscaffold_001566 (15.5 kb, < 85% identical to any sequenced nematode). During assembly, *flye* identified all three as circular. The hicscaffold_001566 scaffold had approximately 100× Nanopore coverage and more than 500× Illumina coverage, which allowed us to reliably annotate this mitochondrial genome. This 15,509 bp, extremely AT‐rich (84.9%), circular genome contained all genes on the same (+) strand, including 12 protein‐coding genes (*ATP8* was missing as is common in nematodes), two rRNAs, and 22 tRNAs (Figure S13). Annotation required careful manual curation because *MITOS2* and *MitoZ* identified only 13 and two tRNAs, respectively. We consider hicscaffold_001276 and hicscaffold_001451 as low‐quality draft sequences due to low sequence coverage of their assembly and polishing data and thus excluded them from further sequence analyses (S23).

### 3.8 ddRAD Processing and Summary Statistics

#### 3.8.1 *D. rufipennis* Population Genomic Dataset

After filtering in *Stacks 2*, we retained 22,579 loci in 284 samples. Of these loci, 12,191 were polymorphic (i.e., they each contained one or more SNPs), and these were retained for further filtering and analysis. Rates of missingness ranged from 2% to 30% for individuals, and between 0% and 19% for loci. Average overall missingness was 4.7%. We assessed the distribution of depth at every filtered locus and removed three loci that exhibited a dramatic jump in depth relative to the rest of the filtered loci. After removal, retained loci exhibited generally continuous variation in depth across genotyped individuals. Mean filtered individual read depth ranged between 11 and 138, and mean filtered locus depth ranged between 10.5 and 3619. Average overall depth in the *D. rufipennis* dataset was 55.

#### 3.8.2 *D. rufipennis* and Outgroup *Dendroctonus* Phylogenetic Dataset

After filtering, we retained a total of 12,071 polymorphic loci in 294 samples—284 *D. rufipennis* and ten outgroup specimens (two specimens each for five species). After filtering, this dataset contained 12,071 filtered SNPs. Rates of missingness ranged from 4% to 96% in individuals, and from 0% to 50% among loci. Outgroup specimens exhibited higher rates of missing data, between 45% and 96% (mean of 69%); among ingroup samples, missingness ranged between 4% and 62% (mean of 15%). Average overall missingness was 17%. Mean individual read depth ranged between 7 and 144, and between 13 and 1943 for loci. Average overall read depth was 58.

### 3.9 Genetic Population Structure and Outlier Analysis in *D. rufipennis*

Principal component analysis identified three primary genomic clusters along the first and second axes (Figure 5A): one that contained all specimens sampled from western Canada [British Columbia (BC) and Alberta (AB)] and Wyoming (WY); a second group of southwestern origin comprised of specimens sampled from Colorado (CO), Utah (UT), and New Mexico (NM); and a third that contained specimens from Nova Scotia (NS) and Alaska (AK). In this third cluster, specimens from Nova Scotia clustered separately from but adjacent to specimens from Alaska, suggesting that these two populations, while closely related, were marginally genetically distinct from one another. PC axis one accounted for 29% of the total variation in the dataset, while axis two accounted for 5.5%. Principal component analysis of the western Canada and Wyoming cluster resolved Wyoming as genetically distinct from specimens sampled in British Columbia and Alberta, but a DAPC of the British Columbia and Albertan specimens alone did not suggest additional substructuring in this region (Figure S14A,B). Similarly, subcluster analysis of the southwestern grouping did not recover additional distinct genomic clusters (Figure S14C,D), though specimens sampled in Utah exhibited less cluster overlap than the Colorado and New Mexico samples and were thus slightly more genetically differentiated.

**FIGURE 5:**
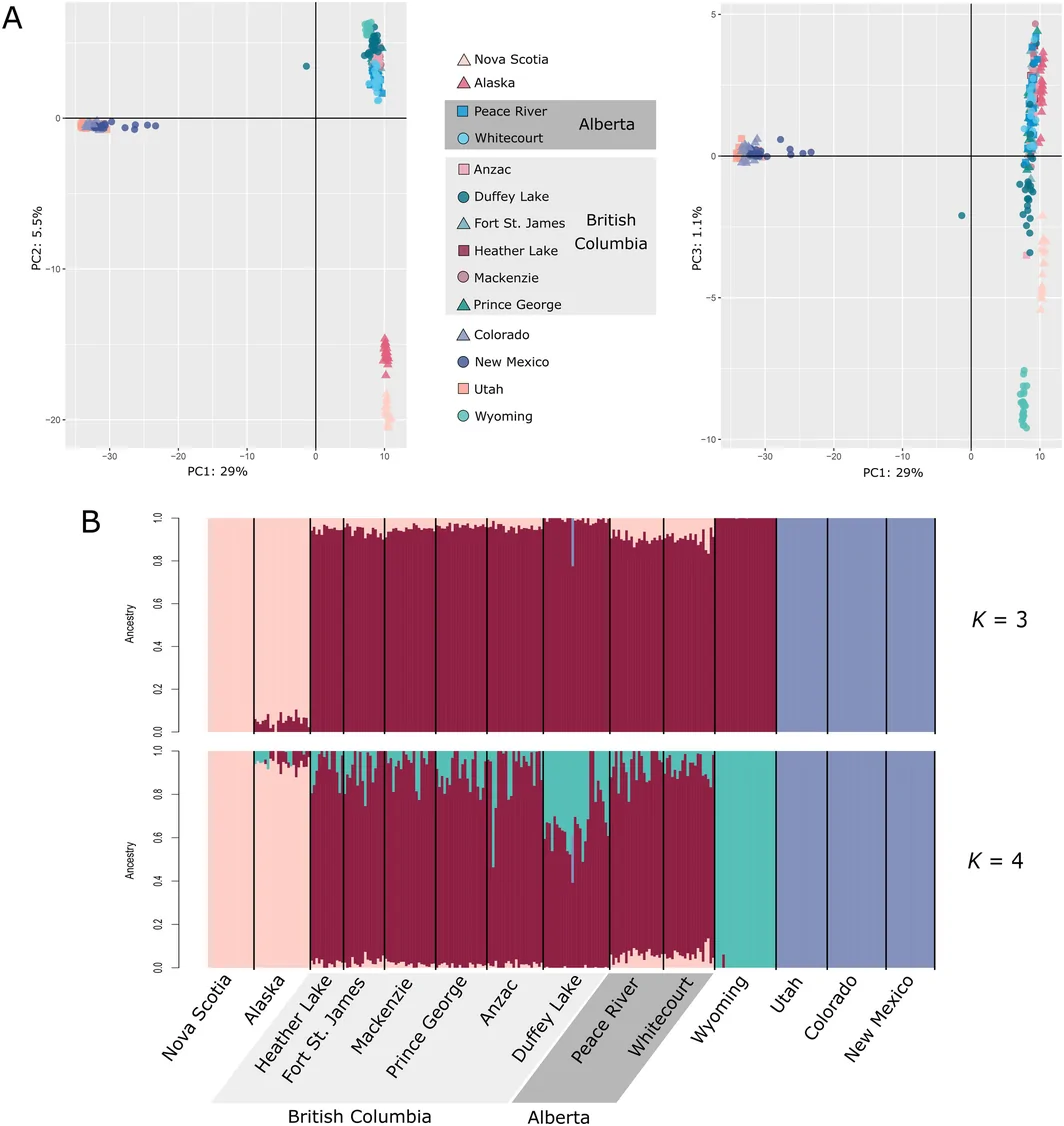
Spruce beetle genetic population structure. (A) Principal component analysis of spruce beetles sampled at 14 locations in Canada and the United States. Axes 1 and 2 distinguish three primary clusters, while Axis three further distinguishes beetles from Wyoming. (B) *ADMIXTURE* analysis for K = 3 and K = 4 demonstrates consistency with the PCA shown in (A).

Cross‐validation with *ADMIXTURE* supported an optimal K = 3, although K = 4 and K = 5 had similar support (Figure S15). At K = 4, Wyoming was resolved as a distinct lineage, while K = 5 began to differentiate Utah from New Mexico and additionally depicted Colorado as primarily admixed between the two other southwestern populations. This was broadly consistent with the PCA sub‐structure analysis for these three localities. For each of these K values, the western Canadian population exhibited admixture, either sharing ancestry with the Nova Scotia/Alaska cluster (K = 3) or both the Nova Scotia/Alaska and Wyoming clusters (K = 4). The Duffey Lake, British Columbia sampling locality exhibited the greatest amount of admixture, and one specimen from this location shared just over 20% genetic ancestry with the southwestern cluster, suggesting possible long‐distance dispersal and hybridization.

Pairwise estimates of *F*_ST_ by collection locality ranged from ~0 to 0.5 and for *d*_xy_ between 0.006 to 0.013. Nucleotide diversity by locality ranged from 0.00543 to 0.0101 (Table S5 in S21). In pairwise comparisons of the four primary populations identified by PCA and *ADMIXTURE* analysis, *F*_ST_ ranged from 0.03 to 0.4 and *d*_xy_ ranged between 0.01 and 0.013, while π for each of the four clusters ranged between 0.00587 and 0.00956 (Table S6 in S21). The Colorado, Utah, and New Mexico localities were highly differentiated from all other sampled groups and exhibited the lowest π. Comparisons between Nova Scotia/Alaska and western Canada, Nova Scotia/Alaska and Wyoming, and western Canada and Wyoming were less differentiated in comparison, and the Wyoming and western Canada populations were the least differentiated overall. However, sliding window *F*_ST_ analysis for these pairwise comparisons illustrated chromosomal regions with spikes in *F*_ST_ values; this was particularly pronounced in the western Canada versus Wyoming comparison (Figure 6). Assessments of *d*_xy_ along each chromosome were less variable than *F*_ST_ and exhibited moderate to high levels of divergence in all comparisons with no obvious spikes or dips across chromosomes (Figure S16).

**FIGURE 6:**
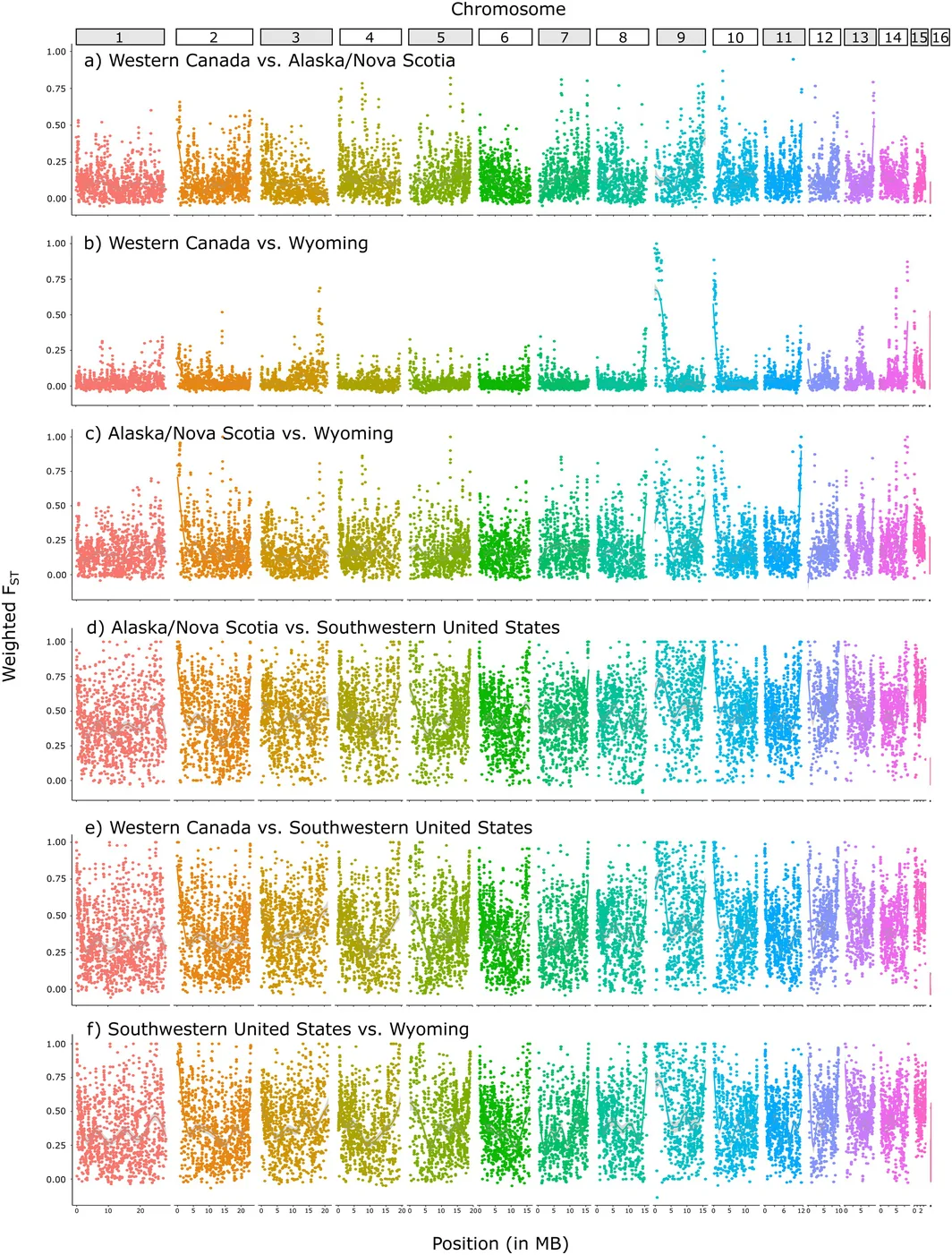
Sliding window *F*_ST_ by chromosome for all major population pairs. Comparison of changes in *F*_ST_ along chromosomes reveals alternate patterns of differentiation between the major spruce beetle populations. The comparisons shown in panels (a), (c), and (d)–(f) demonstrate moderate to high levels of differentiation across most of the genome, consistent with putatively neutral processes leading to divergence between populations. In contrast, the comparison between western Canada and Wyoming, shown in panel (b), illustrates overall low levels of differentiation across the genome, with the exception of a few regions, most notably on chromosomes 9 and 10. This suggests an alternate pattern of differentiation that may reflect non‐neutral processes driving divergence in this region.

*F*_ST_ ‐based outlier analysis of the western Canadian and Wyoming populations identified 77 putative outlier loci (Figure S17; [Link], [Link]); the majority of these were on chromosomes 9 and 10. Analysis of the western Canada vs. Nova Scotia/Alaska populations identified three putative outlier SNPs, one on chromosome 10 and two on chromosome 11, and seven putative outliers were identified between Nova Scotia/Alaska and Wyoming; of these, five were located on chromosome 11. There was very little overlap in the outliers detected for each of the three pairwise comparisons we performed—only two putative outlier SNPs located on chromosome 11 were shared between the Nova Scotia/Alaska vs. Wyoming and the Nova Scotia/Alaska vs. western Canada comparisons.

### 3.10 Phylogenetic Analysis of *D. rufipennis*

Model testing in *IQ‐TREE2* identified GTR + F + R6 as the best‐fitting model under the Bayesian Information Criterion (BIC), and overall the phylogeny was well‐resolved. The clade containing *D. simplex* and *D. pseudotsugae* was used to root the analysis, following the phylogenetic relationships depicted in Ramírez‐Reyes et al. (2023). Each of the six species included in this analysis formed highly supported monophyletic groupings (Figure 7). *D. pseudotsugae* and *D. simplex* together formed a clade, while a second clade contained the remaining species, *D. rufipennis, D. micans, D. punctatus, and D. murrayanae; D. rufipennis* was the earliest branching lineage within this second clade. Within *D. rufipennis* , the analysis indicated a well‐supported split into two primary groupings: one clade containing specimens sampled from the southwestern portion of the *D. rufipennis* range (CO, UT, NM), and another large clade that contained specimens sampled from northern and eastern portions of the species range (AK, BC, AB, WY, NS). These primary groupings were highly supported and very distinct from each other, evidenced by the long branch connecting both clades to the root of the *D. rufipennis* clade. The southwestern clade itself was largely unresolved by locality, though 13 out of the 20 UT specimens formed a distinct, albeit only moderately supported, grouping. The second clade was also similarly unresolved by locality, with two exceptions: the Wyoming specimens formed a well‐supported sub‐clade (though a single specimen from British Columbia and one from Colorado also clustered with the Wyoming samples); and a second, more recently‐diverged and highly‐supported clade depicted the Alaskan and Nova Scotian samples as monophyletic sister groupings distinct from the BC, AB, and WY localities. Overall, the phylogenetic relationships depicted on the tree were highly consistent with assessments of population genetic structuring.

**FIGURE 7:**
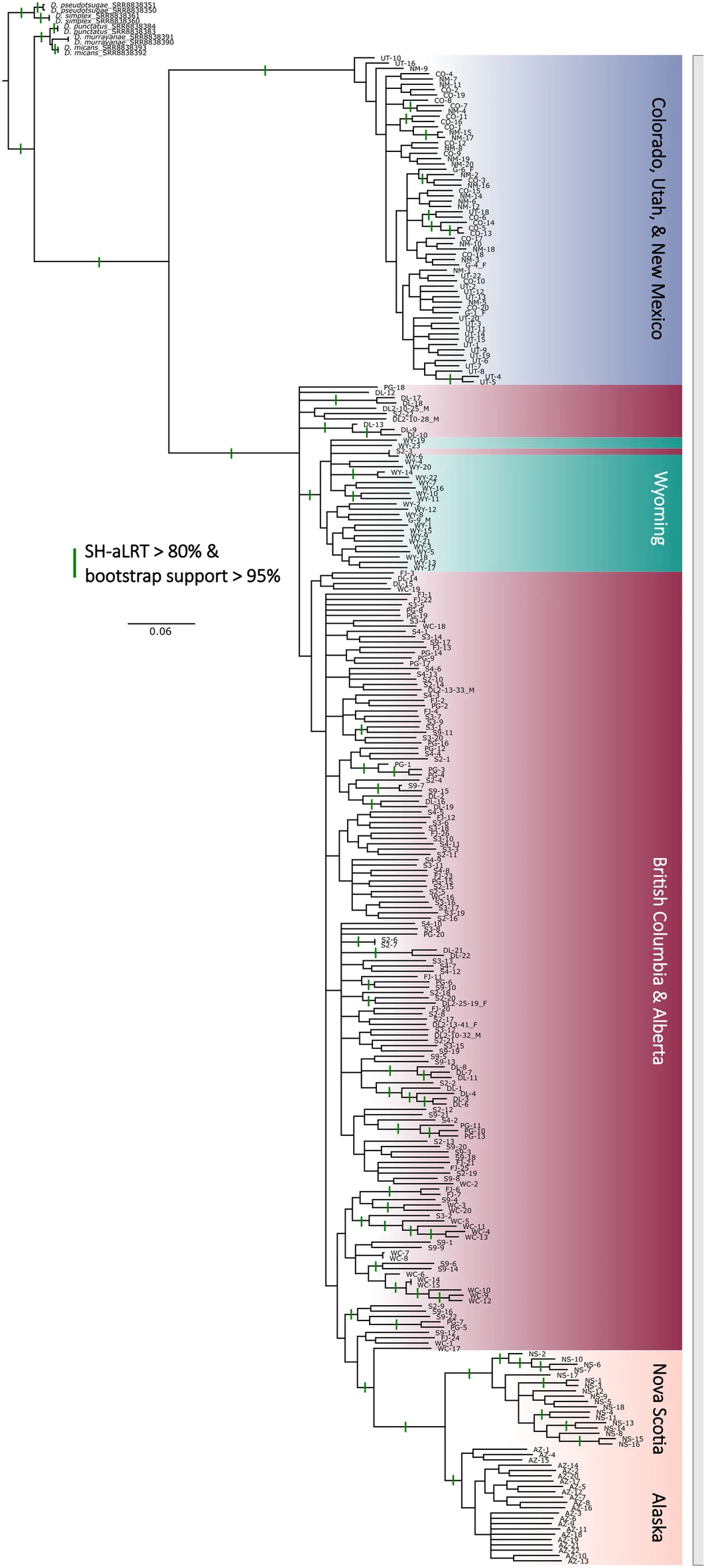
Spruce beetle population‐level phylogeny. Phylogenetic analysis recovered three major groupings and demonstrates a large, highly supported split between the southwestern United States populations from all other sampled localities. Both the southwestern clade and the clade containing Alaska and Nova Scotia beetles were monophyletic, while the grouping containing beetles sampled from western Canada and Wyoming was less resolved. Sample codes and additional sample information in Table S1 in S21.

## 4 Discussion

The spruce beetle is one of the most important insects in North American forest habitats. Its ability to kill standing spruce, use recently fallen trees, and increase populations after windthrow or other disturbances significantly impacts stand structure, succession, nutrient cycling, and carbon dynamics. These stand‐level effects provide important ecosystem services to humans by maintaining spruce forests for recreation, subsistence, carbon sequestration, and fibre production. In recent years, climate change and past forest management practices have been factors in increased spruce beetle infestations, which have local and regional economic and social impacts (Bleiker and Brooks 2021).

The spruce beetle also represents an interesting species in terms of evolutionary research. The insect is able to use every spruce species in its range as a host, which creates a continent‐wide co‐evolutionary mosaic (Thompson 2005). Like other bark beetles, it is itself a host to many symbiotic co‐evolutionary relationships with mites, fungi, other microorganisms, nematodes, and other biota (Cardoza et al. 2006, 2008). The spruce beetle shows some phenotypic evidence of these complex interactions, for instance in its variable aggregation pheromone emissions and responses (Isitt et al. 2020, 2026; Sullivan 2024). It was our objective to produce and analyse a high‐quality spruce beetle genome sequence and to provide a new, higher resolution population genetics analysis to further our understanding of some of the aspects of this ecologically and economically important forest insect.

### 4.1 Assembly

Our use of several sequencing technologies and assembly methods resulted in a final, annotated, near chromosome‐level genome assembly of 271 Mb, with 86% of the assembly contained in 16 pseudochromosomes and containing 17,778 predicted gene models. Although our long‐read Nanopore had an N50 of 8632 bp, the addition of linked‐read and proximity ligation sequencing data improved assembly contiguity approximately ten‐fold. The assembly size was consistent with *k*‐mer based estimations of 281 Mb but fell short of the 361 Mb predicted by flow cytometry. The accuracy of the flow cytometry results may have been affected by our use of an external size standard rather than the preferred internal standard.

### 4.2 Shared Synteny and Karyotypes With Other *Dendroctonus* spp.

The genus *Dendroctonus* contains at least eight different karyotypes among at least 21 species. Karyotypes vary from 5 AA + neo‐XY to 14 AA + Xy_p_, with the latter thought to be ancestral (Zúñiga, Cisneros, et al. 2002; Zúñiga, Salinas‐Moreno, et al. 2002). Our assembly of the spruce beetle genome highlights the differences in genome structure compared to the mountain pine beetle genome ( *D. ponderosae* ), the red turpentine beetle ( *D. valens* ), and the southern pine beetle ( *D. frontalis* ) (Figure 3). *D. rufipennis* has the ancestral 14 AA + Xy_p_ karyotype, whereas *D. valens* , *D. ponderosae* and *D. frontalis* have 13 AA + Xy_p_,11 AA + neo‐XY, and 7 AA + Xy_p_ karyotypes, respectively.

The evolution of sex chromosomes in beetles and other insects is actively being studied (Blackmon et al. 2016; Bracewell et al. 2024). Lanier (1981) suggested that the neo‐XY sex chromosomes in *D. ponderosae* originated from the fusion of an ancestral autosome with the ancestral X‐chromosome to make the neo‐X chromosome, and that the Y‐chromosome was lost and replaced with the other copy of the ancestral autosome as the neo‐Y. However, that ancestral autosome of *D. ponderosae* appears orthologous to not one, but three, autosomes in *D. rufipennis* (1, 11, and 12), indicative of a more complicated origin than first thought (Figure S18A). The ancestral X region of the mountain pine beetle neo‐X corresponds to one of two regions in the genome with increased differentiation (F_ST_) across the genome (Keeling et al. 2022). In comparison to our *D. rufipennis* assembly, the other region with increased differentiation in *D. ponderosae* (in scaffold 4) (Keeling et al. 2022) does not correspond to a fusion event.

*D. frontalis* appears to have retained the original and separate X‐chromosome like *D. rufipennis* , but also shows fusion of the equivalent scaffolds 1, 6, and 12 from *D. rufipennis* . We also observe three other chromosomes with evidence of fusions in *D. frontalis* , equivalent to fusions of scaffolds 2 and 10; 3 and 15; and 5, 11, 13 and 14 in *D. rufipennis* (Figure S18B). We postulate that the fusion of the equivalent of scaffolds 1 and 12 from *D. rufipennis* , found in both *D. ponderosae* and *D. frontalis* , originated before those two species diverged. However, this fusion is not the origin of the 13 AA + Xy_p_ karyotype in *D. valens* (Liu et al. 2022), which is instead the fusion of the equivalent scaffolds 10 and 15 in *D. rufipennis* (Figure S18C). Additional chromosome‐level assemblies are needed to understand how and when karyotypes diversified in this genus, and how it might relate to speciation, the diversity across the genus for host use, colonization behaviour, and geographical range (Godefroid et al. 2019; Ramírez‐Reyes et al. 2023; Zúñiga, Cisneros, et al. 2002).

### 4.3 Mitochondrial Genome of *D. rufipennis*

Morgan et al. (2022) have suggested that coleopteran mitochondrial genomes previously assembled only from short‐read data may not properly assemble repetitive regions and should be confirmed with long‐read data. Compared to the previously published *D. rufipennis* mitochondrial genome only using short‐read data (Meng et al. 2023), we identified a longer region of repeats using Nanopore data. However, the gene order and orientation were consistent with the mitochondrial genomes of other bark beetles including *D. micans* (Du et al. 2021).

### 4.4 Co‐Sequenced Organisms

Genome sequencing of wild‐collected insects can present challenges. There is no opportunity for reducing genetic heterozygosity through inbreeding during laboratory rearing. In addition, we co‐sequenced several other organisms associated with spruce beetles, including bacteria, yeast, and nematodes. Identifying and removing these sequences was not trivial, especially because most of those symbiotic species are not well‐characterized and lack existing sequence data, but the work was greatly aided by tools such as *FCS‐GX* (Astashyn et al. 2024). During that process, we were able to recover a nearly chromosome‐level whole genome of a nematode and the complete mitochondrial genomes from three nematode species with varying degrees of quality. Differential abundance of the various nematode associates between the different samples that were analysed with different sequencing technologies likely explains why the *Contortylenchus* sp. assembly was more complete and contiguous than those of the other nematode species.

Although *Contortylenchus reversus* (Allantonematidae) has been described in *D. rufipennis* previously (Thong and Webster 1983), we lacked morphological data to definitively assign the sequence that we recovered to this species. The only other available sequence data for this genus at the identified species level is for *C. genitalicola* (Kanzaki et al. 2023). We thus describe the nematode species here, pending further identification, as *Contortylenchus* sp. T'it'q'et, in recognition of the T'it'q'et First Nation (P'egp'íg'lha of the St'át'imc Nation) in British Columbia's southern central interior where the organism was sampled.

*Contortylenchus genitalicola* parasitizes gonads of the Japanese pine sawyer, *Monochamus alternatus* Hope 1842 (Coleoptera: Cerambycidae) (Kanzaki et al. 2023). Several other *Contortylenchus* spp. are endoparasites of other bark beetles (Grucmanová and Holuša 2013; for reviews see Kosaka and Kanzaki 2019). In some cases, individual beetles can carry over a thousand nematodes, which can have negative physiological effects on bark beetle hosts (Resnerová et al. 2022; Thong and Webster 1972, 1975a, 1975b) and may also explain our recovery of a complete genome for one *Contortylenchus* species. While we do not know the specific taxonomic identity of the nematode that we sequenced, it is likely that it, too, is an endoparasite like others in its genus. Our full genome sequence for *Contortylenchus* sp. T'it'q'et, plus the location where its host beetle was collected, should provide enough information to recover specimens for further study. The only other nuclear genome published to date in the family Allantonematidae is from *Bradynema listronoti*.

There are no mitochondrial genomes published to date in the family Allantonematidae. However, several are published within the infraorder Tylenchomorpha, which contains the Allantonematidae. Their gene order and compositional diversity varies, even within the same genus (Gendron et al. 2024; Kern et al. 2020). The gene order in the *Contortylenchus* sp. T'it'q'et mitochondrion genome described here closely followed, but was not identical to, the gene order of described species in the family Aphelenchoididae (Figure S13). At 84.9%, the AT‐content of this mitochondrial genome approaches the maximum previously reported in a nematode of 85.4% (Jacob et al. 2009).

The other identified non‐insect sequences originate from organisms known to be associated with bark beetles including: fungi, *Yamadazyma* spp. (Davis 2015; Pineda‐Mendoza et al. 2024); bacteria, *Rickettsia* spp. (Mann et al. 2025), *Rouxiella* spp. (Dohet et al. 2016), and *Rahnella* spp. (Pineda‐Mendoza et al. 2022; Rivera‐Orduña et al. 2023); and nematodes, *Bursaphelenchus* spp. (Kanzaki et al. 2008; Shimizu et al. 2013) and *Ektaphelenchus* spp. (Kanzaki et al. 2008). Unfortunately, limited data for these organisms prevented further analysis.

### 4.5 Cytochromes P450 and Carboxyl/Cholinesterases Gene Families

Both cytochromes P450 and carboxyl/cholinesterase (CCE) gene families have many roles in bark beetle biology, but are of particular interest in pheromone biosynthesis and the detoxification of host defences (Chiu et al. 2017, 2018, 2019a, 2019b, 2019c; Torres‐Banda et al. 2022). When compared to the P450s annotated in other coleopteran genomes and *Dendroctonus* genomes in particular, there was a one‐to‐one correspondence of orthologues for most of the 71 P450s annotated in *D. rufipennis* (Figures S19, S25). One notable exception was the absence of CYP4CV2 in *D. rufipennis* , whereas other *Dendroctonus* appear to have two CYP4CVs. We found eight CYP9Zs clustered together within 90 kb on scaffold 1. This clustering and diversification appears to be a bloom specific to *Dendroctonus*. Although their function has yet to be determined for CYP9Zs in vitro, multiple studies show that some CYP9s have roles in xenobiotic detoxification and insecticide resistance in insects (Sandstrom et al. 2006).

CCEs have a broad range of functions but some are involved in the degradation of host secondary metabolite and olfactory signal transformations, as well as pesticide resistance (Cruse et al. 2023). The coleopteran CCEs in the ESTHER database are limited in number. However, all those previously identified in *D. ponderosae* had a one‐to‐one orthology with each of the 81 annotated *D. rufipennis* CCEs (Figure S20). In almost all cases, one‐to‐one coleopteran orthologues were adjacent to all deduced SB proteins in the phylogeny. However, some carboxylesterases in Block C showed signs of independent diversification within the Coleoptera. *D. rufipennis* has close to the greatest number of CCE genes reported in any insect (Cruse et al. 2023).

### 4.6 Genetic Population Structure

Our results support four primary populations of spruce beetles across North America and are broadly consistent with those of Maroja et al. (2007), who described three populations: the Northern 1 population found in Alaska, northern British Columbia, Yukon, Minnesota, Maine, Newfoundland and Labrador primarily using black, Engelmann, and white spruce as hosts; the Northern 2 population, partly sympatric with the Northern 1 population and found in Alaska, Newfoundland and Labrador, Minnesota, Maine, Michigan, and Yukon primarily using black and white spruce as hosts; and the Rockies population found in Arizona, Utah, southern British Columbia, Colorado, Washington, and Montana and primarily using Engelmann, white, blue, black, and sitka spruce as hosts. Maroja et al. (2007) suggested three range expansions from refugia following the most recent glaciation of North America: from south of the glacial boundary in the current central and eastern United States; from Beringia via Alaska; and from south of the glacial boundary in the Pacific Northwest.

The beetles that we sampled in our study clustered into a north/eastern population (similar to Northern 1) in Alaska and Nova Scotia, a northwestern population (similar to Northern 2) in British Columbia, Alberta, and Wyoming (though substructure analysis did resolve the Wyoming beetles as a distinct population), and a southwestern population (similar to Rockies) in Colorado, Utah, and New Mexico (Figure 1).

Estimates of nucleotide diversity were markedly lower for the southwestern population compared to other populations. This, coupled with high genetic differentiation from the other populations, suggests that the southwestern beetles experienced a significant bottleneck at some point and continue to exhibit reduced within‐population diversity today. This geographic region exhibits fragmented and discontinuous spruce stands and contains the unique blue spruce with a limited natural geographic range. Maroja et al. (2007) suggested that reproductively isolated populations of spruce beetles result from being isolated in small spruce forests during dynamic glaciation, and our results indicate the possibility of incipient or complete allopatric speciation of insects that we assayed in Colorado, Utah, and New Mexico compared to more northern populations.

Estimates of nucleotide diversity in the western Canadian and Wyoming populations were similar and suggestive of moderately high levels of diversity, while overall pairwise *F*_ST_ was low despite the presence of a few highly differentiated chromosomal regions. Sliding window *d*_xy_ estimates were moderately high between these populations and did not vary much across the genome, even in regions containing outlier SNPs. Together, this suggests that identified islands of genomic differentiation are not likely driven by within‐population differences in genetic diversity, but rather by some adaptive process (Cruickshank and Hahn 2014). Interestingly, the overall high genetic similarity of the Wyoming and western Canadian populations is likely the reason we were able to detect these islands of differentiation. Though we did not observe patterns of the same magnitude at these genomic locations in other pairwise population comparisons, it is possible that they are being masked due to significantly higher genome‐wide differentiation between them. This could suggest that the Wyoming or western Canadian population has experienced a selective sweep or acquired some type of structural variant that limits recombination in these chromosomal regions. Whole genome sequencing would help characterize these patterns further.

Our results also identified southern British Columbia as a transition zone for spruce beetle populations, coinciding with the white/Engelmann spruce hybrid zone, further demonstrating the complexities of population interactions across the range of this insect. This supports the hypothesis of Maroja et al. (2007) that the Pacific Northwest is a region where spruce beetle populations have repeatedly had reproductive contact.

### 4.7 SNPs in Coding Regions

Many of the outlier SNPs (S24) that fell within or near annotated regions of the genome could have substantial effects on the insect's physiology and behaviour. For instance, several SNPs were associated with gene or mRNA regulation (a transcription factor, a chromatin remodelling ATP‐ase, and a putative nucleoporin). Several SNPs were associated with genes whose products could be involved in regulating protein activity (protein kinase or kinase‐associated genes, a putative F‐box protein) or protein degradation (ubiquitin‐associated proteins). Several are possibly involved in basic cell processes that could impact energetics, such as lipid metabolism, intracellular vesicular trafficking, and cell signalling. One SNP was present in an ATP‐binding cassette (ABC protein), some of which are involved in the transport of secondary metabolites across membranes during detoxification processes.

While the above annotations are to proteins that are thus far uncharacterized in scolytines, one SNP was found in a homologue with a well‐characterized mountain pine beetle ( *D. ponderosae* ) cytochrome P450—CYP4G56 (MacLean et al. 2018). This cytochrome P450, in conjunction with CYP4G55 (homologue also present in the spruce beetle genome), can convert ten‐carbon alcohols and aldehydes to a variety of hydrocarbon products. One of its products—3‐nonene—is part of a pathway in the insect that produces a precursor for *exo*‐brevicomin. Some of its other products are commonly found as insect cuticular hydrocarbons.

*exo*‐Brevicomin is a component of the aggregation pheromone produced by male, and possibly female, mountain pine beetles and is attractive to both sexes (Pureswaran et al. 2000; Sullivan 2024; Vité and Pitman 1969). Cuticular hydrocarbons play a number of biological roles in insects, including in close‐contact, gustatory intraspecific communication (Martin and Drijfhout 2009). While *exo*‐brevicomin is not known to be an attractive component of the spruce beetle pheromone blend, this species is able to produce it when exposed to its biosynthetic precursor (Perez et al. 1996). This cytochrome P450 has the potential for further study related to variation in both aggregative (Isitt et al. 2018, 2020) direct‐contact spruce beetle chemical communication.

## 5 Conclusion

We developed a high‐quality genomic sequence and a detailed population genomics analysis across the spruce beetle's range, giving us new insights into its ecology and evolution. This also provides a solid foundation for further study of this important insect. The chromosome‐level scaffolds of this spruce beetle genome, along with those of other *Dendroctonus* species, reveal the evolutionary history of several *Dendroctonus* species, including the evolution of crucial gene families like cytochromes P450 and carboxyl/cholinesterases. Our SNP results align with previous work (Maroja et al. 2007) but offer more detailed population‐level insights into this insect that inhabits various spruce species across a vast geography. The SNP analysis shows that the southernmost insect population in our study, with its fragmented habitat and limited‐range spruce host, has high genomic divergence compared to other sampled populations. We have identified several differentiated genomic regions with potential physiological or behavioural consequences. We have also provided a chromosome‐level sequence for a spruce beetle associate and several genomic sequences for various symbionts. This comprehensive genomic and population‐level analysis of the spruce beetle will provide data resources for ongoing and future physiological and ecological studies of this ecologically and economically important insect.

## Author Contributions

**Erin O. Campbell:** data curation, formal analysis, methodology, software, validation, visualization, writing – original draft, writing – review and editing. **Philip D. Batista:** data curation, formal analysis, investigation, methodology, resources, writing – review and editing. **Véronique Lévesque‐Tremblay:** investigation, methodology. **Catherine Béliveau:** data curation, formal analysis. **Kathia Bernier:** data curation, formal analysis. **Kateryna Romanenko:** investigation, methodology. **Ward B. Strong:** conceptualization, funding acquisition. **Katherine P. Bleiker:** conceptualization, funding acquisition, resources, visualization, writing – original draft, writing – review and editing. **Dezene P. W. Huber:** conceptualization, funding acquisition, project administration, resources, supervision, writing – original draft, writing – review and editing. **Christopher I. Keeling:** conceptualization, data curation, formal analysis, funding acquisition, investigation, methodology, project administration, resources, software, supervision, validation, visualization, writing – original draft, writing – review and editing.

## Funding

This study was supported by Natural Resources Canada: A‐base (CIK and KPB), Genomics and Research Development Initiative (CIK and KPB), Canada‐Ukraine Science Partnership program (KR); Genome BC: Sector Innovation Program (Intake 3, WBS, CIK, DPWH); Natural Sciences and Engineering Research Council of Canada (to CIK, RGPIN‐2020‐04260 and DPWH, RGPIN‐2020‐04891). This research was enabled in part by support provided by the BC Digital Research Infrastructure Group and the Digital Research Alliance of Canada (alliancecan.ca), formerly Compute Canada.

## Ethics Statement

All insect specimens utilized in this study were collected with adherence to local and international guidelines regarding conservation and biodiversity preservation. The research was designed to minimize environmental disruption, and no endangered or protected species were harmed during this research. All raw sequence data and associated metadata have been deposited in publicly accessible databases (NCBI) to ensure open‐access scientific transparency. Additional collected specimens not used in this study have been submitted to the Spencer Entomological Collection in the Beaty Biodiversity Museum at the University of British Columbia.

## Conflicts of Interest

The authors declare no conflicts of interest.

## Supporting information


**Figure S1:** Genome assembly schematic. Workflow used to assemble and annotate the genome. Each step is indicated in blue, with the software used shown in italics. The data used at each step are shown in red (with NCBI sample and sequence accessions, if applicable). See main text for further details.

**Figure S2:** Hi‐C contact heatmap. The contacts are shown for the combined Chicago and Hi‐C data at the *Reviewed Hi‐C‐assembly* step (see Figure S1). The 16 pseudoscaffolds of spruce beetle are shown (numbered in black to the right), followed by the nine nematode pseudoscaffolds (numbered in purple to the left), and then the smaller scaffolds of the draft assembly. The heatmap was generated with *Juicebox* at 100 kb resolution with coverage normalization. Green boxes indicate scaffolds from *ARCS‐scaffolded* assembly and blue boxes indicate the scaffolds from the *Reviewed Hi‐C‐assembly*. To provide a more detailed view of the full heatmap, we took multiple screenshots at this resolution and merged the image tiles with OmniGraffle (v7.25, The Omni Group).

**Figure S3:** Schematic of contamination identification using *PhylOligo* and *FCS‐GX*. (A) Clusters generated by *hdbscan* with *PhylOligo* with decreasing minimum scaffold size; (B) Summary of main taxonomic grouping of each cluster from *PhylOligo* based on *BLASTn* searches against NCBI nt. Thick arrows link the same cluster members between scaffold sizes and thin arrows indicate a change of cluster membership for a few scaffolds; (C) *FCS‐GX* identified 430 scaffolds as not being from *D. rufipennis* (taxid 77170) and indicated closest species matching in its dataset. The bar graph shows proportion of each *FCS‐GX* taxonomic grouping in the 1 kbp + *PhylOligo* cluster membership.

**Figure S4:** Benchmarking Universal Single‐Copy Orthologs (BUSCO) results for the spruce beetle genome *BRAKER2* and annotations. Presence of conserved single‐copy orthologs in the genome assembly and the transcripts from *BRAKER2* annotation (including splice variants). Analyses completed with *BUSCO* v6.0.0 and the coleoptera_odb12, 2025‐07‐01, *n* = 3729 database. The E metric refers to the percentage of complete BUSCOs that contain internal stop codons.

**Figure S5:** Genome size estimation based on *k*‐mer counts. (A) *GenomeScope 2.0* predictions of genome haploid length based on *k*‐mer counts calculated by *FastK*; (B) *GenomeScope 2.0* outputs where *k* = 31.

**Figure S6:** Sequencing coverage comparisons between male and female samples. The plot shows an equal normalized coverage for male and female samples across most scaffolds, but a two‐fold higher coverage for female versus male for Druf_M_20221006_9, suggesting the X chromosome, and low female coverage for Druf_M_20221006_16, suggesting the Y chromosome. AC, Adjustment Coefficient = 1.331, a factor to correct for the difference in overall coverage between male and female samples (mode of 142 for female and 189 for male).

**Figure S7:** Spruce beetle intron splice sites consensus sequence and intron lengths. We calculated the consensus intron donor (A) and acceptor (B) splice sites from the *BRAKER2* annotations. (C) Histogram of the intron lengths.

**Figure S8:** Mitochondrion genome of *D. rufipennis* . The circular mitochondrial genome, annotated with protein‐coding genes (13, shown in blue) and non‐coding genes (22 tRNAs and 2 rRNAs, shown in red). The predicted anticodons are shown in brackets for the tRNAs. A 4877 bp AT‐rich region is shown in green, which contains two repeat regions, in pink: a 2443 bp region with a 40 bp repeat and a 941 bp region with an imperfect 150 bp repeat.

**Figure S9:** Phylogeny of *18S* rRNA gene sequences in Tylenchomorpha. We aligned the non‐redundant nematode Tylenchomorpha *18S* rRNA gene sequences from NemaBase with our three nematode *18S* sequences (in red) using *mafft* and prepared a maximum‐likelihood phylogeny with *FastTree2*. The circle diameter and colour at each node indicates the relative Shimodaira‐Hasegawa‐like local support value (bootstrap, between 0 and 1). We used *FigTree* to visualize the tree. We used the *18S* sequence from *Panagrolaimus davidi* (from Panagrolaimomorpha, in blue) as an outgroup to root the tree.

**Figure S10:** Nematode genome assembly statistic plots. Genome assembly statistics for (A) *Contortylenchus* sp. T'it'q'et assembly; (B) the other remaining nematode sequences. A red segment represents the longest scaffold while all other scaffolds are arranged in size‐order moving clockwise around the plot and drawn in grey starting from the outside of the central plot. Dark and light orange arcs show the N50 and N90 values. The central light grey spiral shows cumulative scaffold count with a white line at each order of magnitude. The dark vs. light blue area around the outside of the plot shows mean, maximum and minimum GC vs. AT content at 0.1% intervals. White regions in this area reflect the proportion of Ns in the assembly. BUSCO scores for the genome assembly are shown in the upper right‐hand corner.

**Figure S11:** Benchmarking Universal Single‐Copy Orthologs (BUSCO) results of *Contortylenchus* sp. T'it'q'et genome and *BRAKER3* annotations. Presence of conserved single‐copy orthologs in the genome assembly and the transcripts from *BRAKER3* annotation (including splice variants). Analyses completed with *BUSCO* v6.0.0 and the nematoda_odb12, 2025‐07‐01, *n* = 596 database. The E metric refers to the percentage of complete BUSCOs that contain internal stop codons.

**Figure S12:** Species phylogeny of Nematoda. Species phylogeny of the phylum Nematoda based on separate phylogenies of single copy orthologous sequences from reference nematode genomes available from NCBI (231 species, representing 105 genera). We used *wASTRAL* to create the species phylogeny, and rooted the tree mid‐branch between the main Enoplea and Chromadorea clusters. *Contortylenchus* sp. T'it'q'et is shown in red. The circle diameter and colour at each node indicates the relative Shimodaira‐Hasegawa‐like local support value (bootstrap, between 0 and 1). Species used in the phylogeny and their genome assembly accessions are shown in S26.

**Figure S13:** Mitochondrial genome of *Contortylenchus* sp. T'it'q'et. The circular mitochondrial genome, annotated with protein‐coding genes (12, shown in blue) and non‐coding genes (22 tRNAs and 2 rRNAs, shown in red). The predicted anticodons are shown in brackets for the tRNAs. A 2330 bp repetitive region is shown in pink. All genes are on the same strand.

**Figure S14:** Substructure analysis of spruce beetle genetic population clusters. (A) PCA of western Canada and Wyoming beetles shows differentiation of the Wyoming population; additional DAPC analysis of the western Canadian beetles only failed to resolve further substructure, as illustrated by the BIC plot in (B). Panel (C) illustrates marginal differentiation between specimens sampled in Utah from those sampled in Colorado and New Mexico, but DAPC did not support additional substructuring, as shown in the BIC plot in (D).

**Figure S15:** *ADMIXTURE* cross‐validation and additional *K* value clustering. (A) Cross‐validation results for *ADMIXTURE* analysis indicate *K* = 3 has the lowest CV error, while *K* = 4 and 5 were similarly supported. Panel (B) shows all three of these *K* values, and demonstrates that even at *K* = 5 the four major groupings hold together. At *K* = 5 Utah demonstrates marginal distinction from Colorado and New Mexico, yet the Nova Scotia and Alaska population hold together despite large geographic distances between these sampling localities.

**Figure S16:** *F*_ST_ and *d*_xy_ plotted by chromosome. Illustration of the relationship between *F*_ST_ and *d*_xy_ for the pairwise comparisons shown in the *OutFLANK* outlier analysis. Overall, *d*_xy_ does not appear to vary much across the genome, and *F*_ST_ islands of differentiation are not accompanied by obvious changes in *d*_xy_.

**Figure S17:** *OutFLANK* outlier analysis by chromosome for each pairwise comparison. Illustration of the outliers identified by *OutFLANK* and their location within the genome. The western Canada & Wyoming comparison had the greatest number of statistically significant outliers, while the western Canada & Alaska/Nova Scotia and the Wyoming & Alaska/Nova Scotia comparisons had far fewer significant outliers. Most of the significant outlier loci were located on chromosomes 9 and 10.

**Figure S18:** Pair‐wise shared synteny between spruce beetle and red turpentine beetle, mountain pine beetle, and southern pine beetle genomes. (A) Shared synteny between *D. rufipennis* (this study) and *D. ponderosae* (Keeling et al. 2022); (B) *D. rufipennis* and *D. frontalis* (Copeland et al. 2024); and (C) *D. rufipennis* and *D. valens* (Liu et al. 2022) assemblies.

**Figure S19:** Phylogeny of cytochrome P450 gene family. We aligned the manually curated and annotated P450s from the spruce beetle genome with representative Coleopteran P450 protein sequences using *MUSCLE* with manual refinement and prepared a maximum‐likelihood phylogeny with *FastTree2*. We used *FigTree* to visualize the phylogeny. The circle diameter and colour at each node indicates the relative Shimodaira‐Hasegawa‐like local support value (bootstrap, between 0 and 1). Agra, *Anthonomus grandis* (Boll weevil); Darm, *Dendroctonus armandi* (Chinese white pine beetle); Dpon, *D. ponderosae* (Mountain pine beetle); Drhi, *D. rhizophagus* ; Druf, *D. rufipennis* (Spruce beetle); Dval, *D. valens* (Red turpentine beetle); Efor, *Euwallacea fornicates* (Polyphagous shot‐hole borer); Sory, *Sitophilus oryzae* (Rice weevil); Tcas, *Tribolium castaneum* (Red flour beetle). Human CYP3A4 was used as an outgroup to root the tree. GenBank accessions of P450s used in phylogeny are listed in S25.

**Figure S20:** Phylogeny of insect carboxylesterases and related enzymes. We aligned the manually curated and annotated carboxylesterases and related enzymes from the spruce beetle genome with representative Insecta protein sequences from the ESTHER database (ESTerases and alpha/beta‐Hydrolase Enzymes and Relatives) using *mafft* and prepared a maximum‐likelihood phylogeny with *FastTree2*. We used *FigTree* to visualize the unrooted tree. Druf_M_20221006_gNNNN indicate the spruce beetle gene models. Those ending with an m or A/B indicate a manually modified or split gene model from the automated annotations, respectively. Labels in red and orange indicate *Dendroctonus rufipennis* and other *Dendroctonus* spp., respectively. Other Coleoptera spp. are shown in blue. Species abbreviations (from ESTHER): acypi, *Acyrthosiphon pisum* (Pea aphid); aedae, *Aedes aegypti* (Yellowfever mosquito); anogl, *Anoplophora glabripennis* (Asian longhorn beetle); apime, *Apis mellifera* (Honeybee); bommo, *Bombyx mori* (Silk moth); denpd, *Dendroctonus ponderosae* (mountain pine beetle); drome, *Drosophila melanogaster* (Fruit fly); lepde, *Leptinotarsa decemlineata* (Colorado potato beetle); locmi, *Locusta migratoria* (Migratory locust); lyghe, *Lygus hesperus* (Western plant bug); pedhb, *Pediculus humanus corporis* (human body louse); pedhc, *Pediculus humanus capitis* (human head louse); solin, *Solenopsis invicta* (Red imported fire ant); spoli, *Spodoptera littoralis* (Egyptian cotton leafworm); tenmo, *Tenebrio molitor* (Yellow mealworm beetle); trica, *Tribolium castaneum* (Red flour beetle); and zoone, *Zootermopsis nevadensis* (Dampwood termite). The circle diameter and colour at each node indicates the relative Shimodaira‐Hasegawa‐like local support value (bootstrap, between 0 and 1). Classification Blocks C, H, L, X, their families, and some subfamilies are indicated. We rooted the tree to the midpoint between Block C and H, L, and X Blocks.

**Supplementary Materials and Methods:** Additional Materials and Methods not appearing in the main text.
**Table S1:** Population genetics sample information. Collection details and sample coding for beetles used in population genetic analyses.
**Table S2:** Spruce beetle repetitive sequences. Summary of repetitive sequences identified by *Repeatmasker* in the *D. rufipennis* genome.
**Table S3:** *Contortylenchus* sp. T'it'q'et genome assembly statistics.
**Table S4:** *Contortylenchus* sp. T'it'q'et repetitive sequences. Summary of repetitive sequences identified by *Repeatmasker* in the *Contortylenchus* sp. T'it'q'et genome.
**Table S5:** Average nucleotide diversity, pairwise *d*_xy_ (upper diagonal) and pairwise *F*_ST_ (lower diagonal) for all sampling localities.
**Table S6:** Average nucleotide diversity, pairwise *d*_xy_ (upper diagonal) and pairwise *F*_ST_ (lower diagonal) for major genetic clusters.
**Table S7:** *Dendroctonus* spp. RADseq accessions used in phylogeny.

**File S1:** Information on the voucher specimens deposited in the Spencer Entomological Collection of the Beaty Biodiversity Museum, University of British Columbia, Vancouver, BC, Canada.

**File S2:** FASTA sequences for the putative low‐quality draft mtDNA genomes of *Ektaphelenchus obtusus* (hicscaffold_001276) and *Bursaphelenchus rufipennis* (hicscaffold_001451).

**File S3:** *OutFLANK* outlier loci and annotation information for pairwise comparisons between western Canada & Wyoming, western Canada & Alaska/Nova Scotia, and Alaska/Nova Scotia & Wyoming. Loci are sorted by statistical significance.

**File S4:** Table of CYP450s for each clan, and their NCBI accession numbers used in Figure S19.

**File S5:** Nematode NCBI accessions used in the species phylogeny of Figure S12.

## Data Availability

Data described within are covered by the Open Government Licence—Canada. The raw sequence data and the annotated *Dendroctonus rufipennis* beetle and *Contortylenchus* sp. T'it'q'et nematode genome assemblies are available at NCBI within BioProject PRJNA640538. The *D. rufipennis* Whole Genome Shotgun project has been deposited at DDBJ/ENA/GenBank under the accession JACBAY000000000. The version described in this paper is version JACBAY010000000. The *Contortylenchus* sp. T'it'q'et Whole Genome Shotgun project has been deposited at DDBJ/ENA/GenBank under the accession JBVVKW000000000. The version described in this paper is version JBVVKW010000000. The complete annotated mitochondrion genomes have been deposited at GenBank under the accessions PV939323 and PX401893 for *D. rufipennis* and *Contortylenchus* sp. T'it'q'et, respectively. The population genetics data are available at NCBI within BioProject PRJNA1375824. NCBI accession numbers for the samples and sequencing data appear below:
Nanopore (PromethION) reads from the GSC (pool of three individual males) [SAMN26681013, SRR18335237] 10× Genomics Chromium Illumina reads from Dovetail Genomics (three males) [SAMN15325384, SRR12053743] Dovetail Genomics Chicago Illumina reads (same three males as 2 above) [SAMN15325384, SRR12053744] Dovetail Genomics Hi‐C Illumina reads (individual male) [SAMN15325521, SRR12053745] 10× Genomics Chromium Illumina reads from GSC (individual male) [SAMN15325870, SRR12054036] 10× Genomics Chromium Illumina reads from GSC (individual female) [SAMN15325942, SRR12054091] ddRAD samples and sequences [SAMN53699806–SAMN53700089, SRR36330985–SRR36331268]. Benefits Generated: Many people and organizations, including indigenous people, contributed to the collection of specimens and are described in the Acknowledgments. Benefits from this research accrue from the sharing of our data and results on public databases as described above. Access to scientific information is relevant to conservation and sustainable use of biological diversity, including biological inventories and taxonomic studies.
