# Supplementary material for: The Genome and Population Structure of the Spruce Beetle, Dendroctonus rufipennis (Kirby, 1837), a Transcontinental Pest of North American Spruce
Source: Mol Ecol Resour. 2026 Oct 29;26(8):e70209. doi: 10.1111/1755-0998.70209 (PMC13717379; doi:10.1111/1755-0998.70209)

Figure S7: Spruce beetle intron splice sites consensus sequence and intron lengths
We calculated the consensus intron donor (A) and acceptor (B) splice sites from the *BRAKER2* annotations. (C) Histogram of the intron lengths.

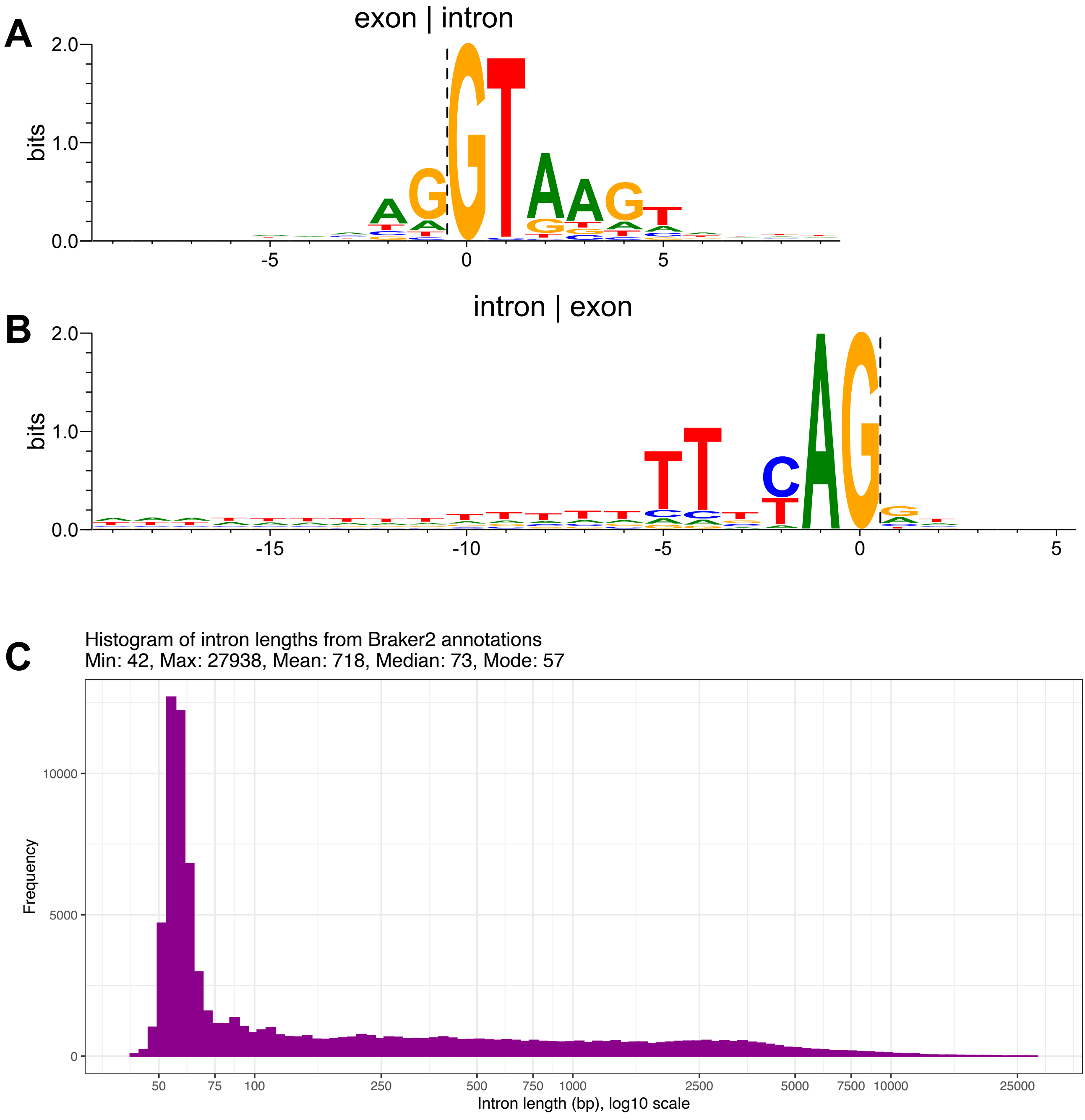

Supplement: Supplementary file 1 [file MEN-26-e70209-s001.pdf]
