# Supplementary material for: The Genome and Population Structure of the Spruce Beetle, Dendroctonus rufipennis (Kirby, 1837), a Transcontinental Pest of North American Spruce
Source: Mol Ecol Resour. 2026 Oct 29;26(8):e70209. doi: 10.1111/1755-0998.70209 (PMC13717379; doi:10.1111/1755-0998.70209)

Figure S13: Mitochondrial genome of *Contortylenchus* sp. T'it'q'et
The circular mitochondrial genome, annotated with protein-coding genes (12, shown in blue) and non-coding genes (22 tRNAs and 2 rRNAs, shown in red). The predicted anticodons are shown in brackets for the tRNAs. A 2,330 bp repetitive region is shown in pink. All genes are on the same strand.

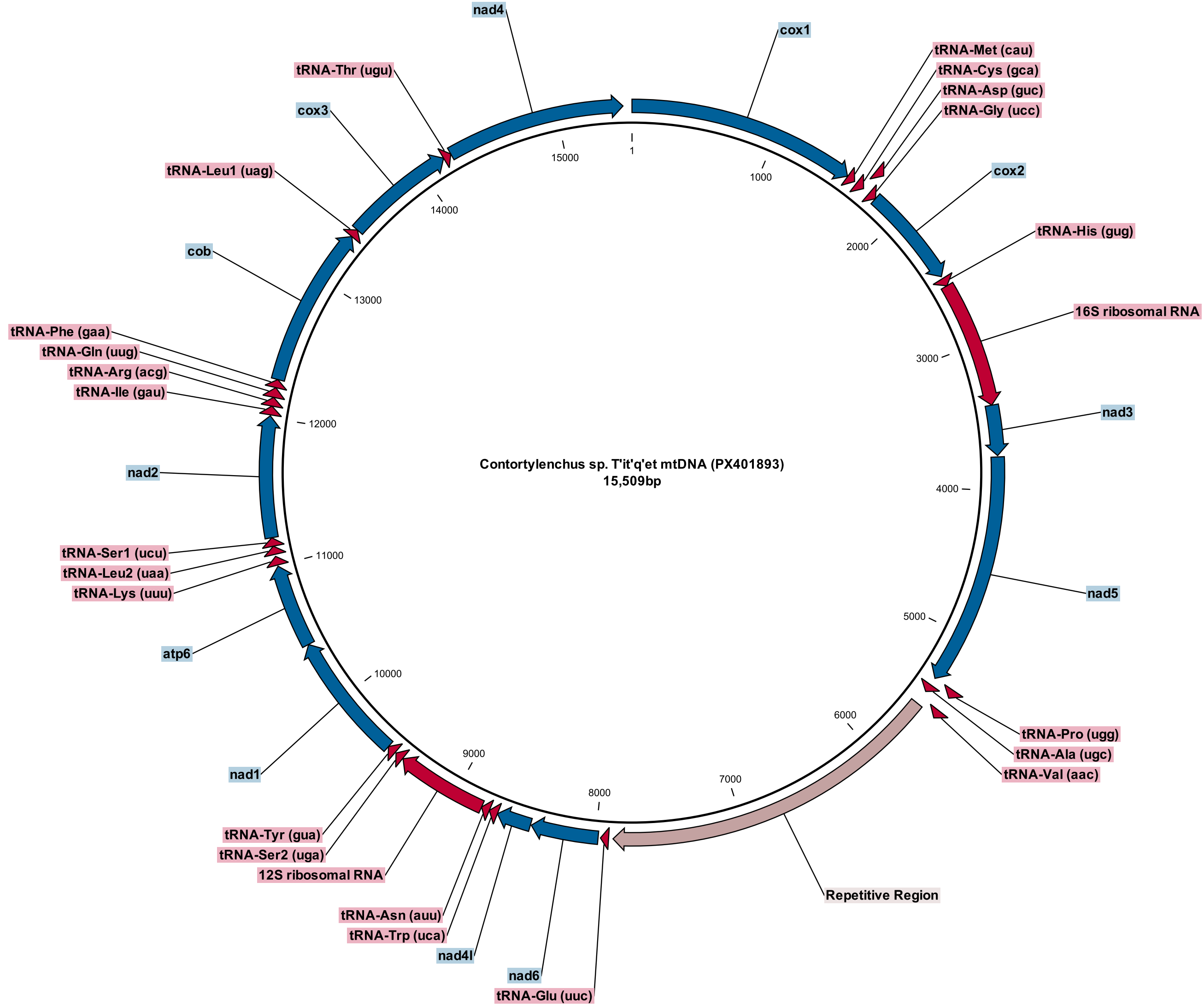

Supplement: Supplementary file 2 [file MEN-26-e70209-s002.pdf]
