# Supplementary material for: The Genome and Population Structure of the Spruce Beetle, Dendroctonus rufipennis (Kirby, 1837), a Transcontinental Pest of North American Spruce
Source: Mol Ecol Resour. 2026 Oct 29;26(8):e70209. doi: 10.1111/1755-0998.70209 (PMC13717379; doi:10.1111/1755-0998.70209)

Figure S4: Benchmarking Universal Single-Copy Orthologs (BUSCO) results for the spruce beetle genome *BRAKER2* and annotations

Presence of conserved single-copy orthologs in the genome assembly and the transcripts from *BRAKER2* annotation (including splice variants). Analyses completed with *BUSCO* v6.0.0 and the coleoptera_odb12, 2025-07-01, n=3729 database. The E metric refers to the percentage of complete BUSCOs that contain internal stop codons.

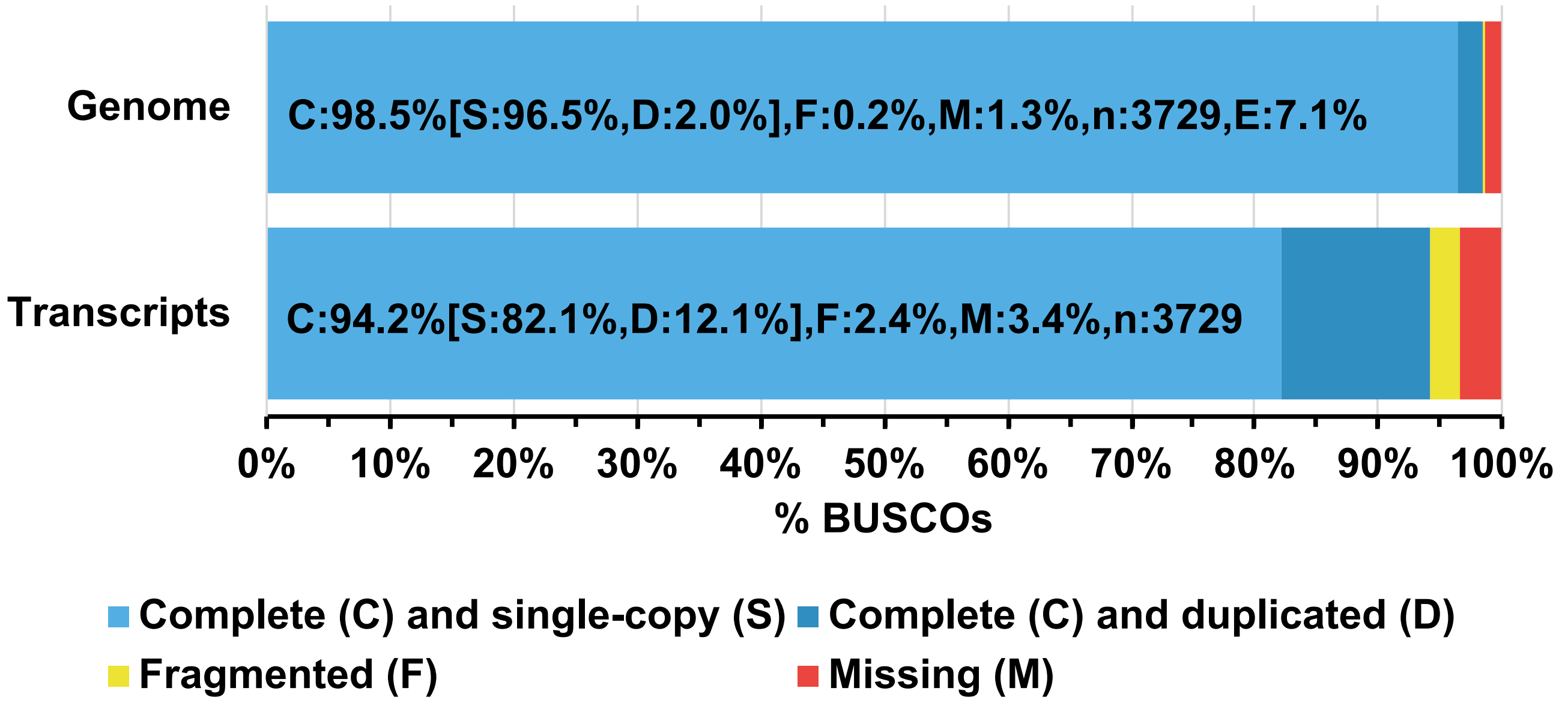

Supplement: Supplementary file 3 [file MEN-26-e70209-s003.pdf]
