# Supplementary material for: The Genome and Population Structure of the Spruce Beetle, Dendroctonus rufipennis (Kirby, 1837), a Transcontinental Pest of North American Spruce
Source: Mol Ecol Resour. 2026 Oct 29;26(8):e70209. doi: 10.1111/1755-0998.70209 (PMC13717379; doi:10.1111/1755-0998.70209)

Figure S17: *OutFLANK* outlier analysis by chromosome for each pairwise comparison
Illustration of the outliers identified by *OutFLANK* and their location within the genome. The western Canada & Wyoming comparison had the greatest number of statistically significant outliers, while the western Canada & Alaska/Nova Scotia and the Wyoming & Alaska/Nova Scotia comparisons had far fewer significant outliers. Most of the significant outlier loci were located on chromosomes 9 and 10.

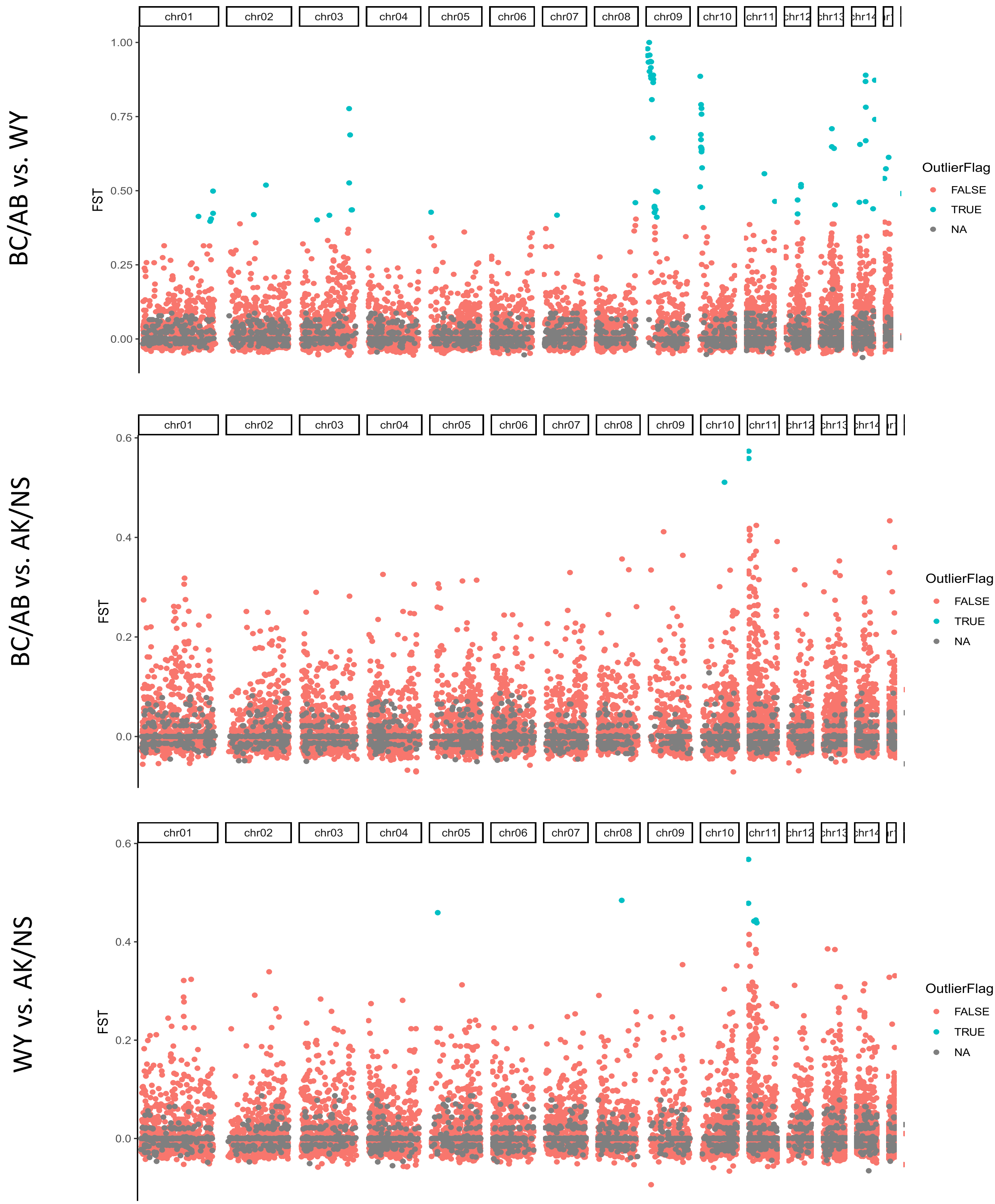

Supplement: Supplementary file 4 [file MEN-26-e70209-s005.pdf]
