# Supplementary material for: The Genome and Population Structure of the Spruce Beetle, Dendroctonus rufipennis (Kirby, 1837), a Transcontinental Pest of North American Spruce
Source: Mol Ecol Resour. 2026 Oct 29;26(8):e70209. doi: 10.1111/1755-0998.70209 (PMC13717379; doi:10.1111/1755-0998.70209)

Figure S2: Hi-C contact heatmap

The contacts are shown for the combined Chicago and Hi-C data at the *Reviewed Hi-C-assembly* step (see Figure S1). The 16 pseudoscaffolds of spruce beetle are shown (numbered in black to the right), followed by the nine nematode pseudoscaffolds (numbered in purple to the left), and then the smaller scaffolds of the draft assembly. The heatmap was generated with *Juicebox* at 100 kb resolution with coverage normalization. Green boxes indicate scaffolds from *ARCS-scaffolded* assembly and blue boxes indicate the scaffolds from the *Reviewed Hi-C-assembly*. To provide a more detailed view of the full heatmap, we took multiple screenshots at this resolution and merged the image tiles with OmniGraffle (v7.25, The Omni Group).

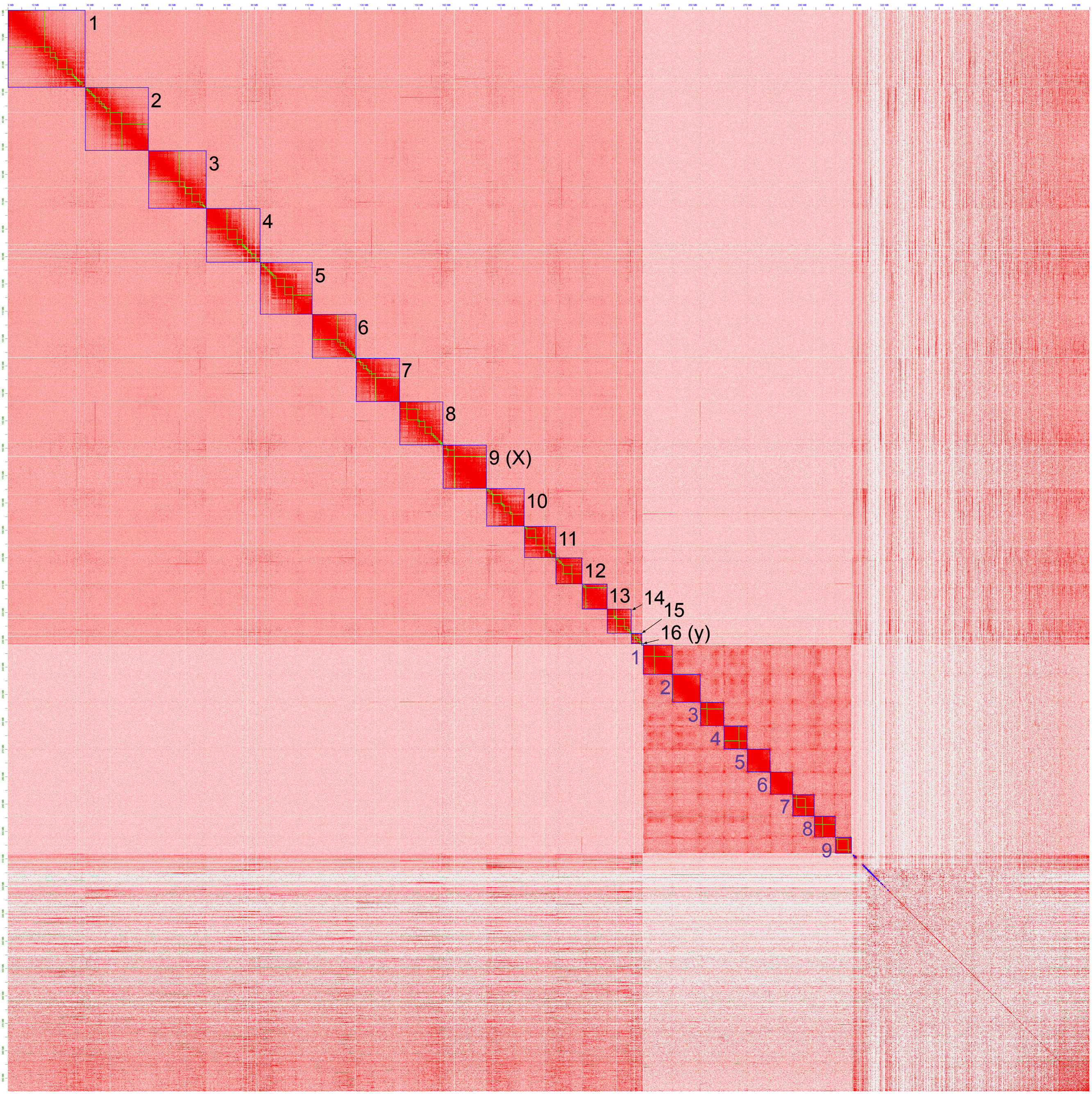

Supplement: Supplementary file 5 [file MEN-26-e70209-s007.pdf]
