# Supplementary material for: The Genome and Population Structure of the Spruce Beetle, Dendroctonus rufipennis (Kirby, 1837), a Transcontinental Pest of North American Spruce
Source: Mol Ecol Resour. 2026 Oct 29;26(8):e70209. doi: 10.1111/1755-0998.70209 (PMC13717379; doi:10.1111/1755-0998.70209)

Figure S10: Nematode genome assembly statistic plots

Genome assembly statistics for (A) *Contortylenchus* sp. T'it'q'et assembly; (B) the other remaining nematode sequences. A red segment represents the longest scaffold while all other scaffolds are arranged in size-order moving clockwise around the plot and drawn in grey starting from the outside of the central plot. Dark and light orange arcs show the N50 and N90 values. The central light grey spiral shows cumulative scaffold count with a white line at each order of magnitude. The dark vs. light blue area around the outside of the plot shows mean, maximum and minimum GC vs. AT content at 0.1% intervals. White regions in this area reflect the proportion of Ns in the assembly. BUSCO scores for the genome assembly are shown in the upper right-hand corner.

A

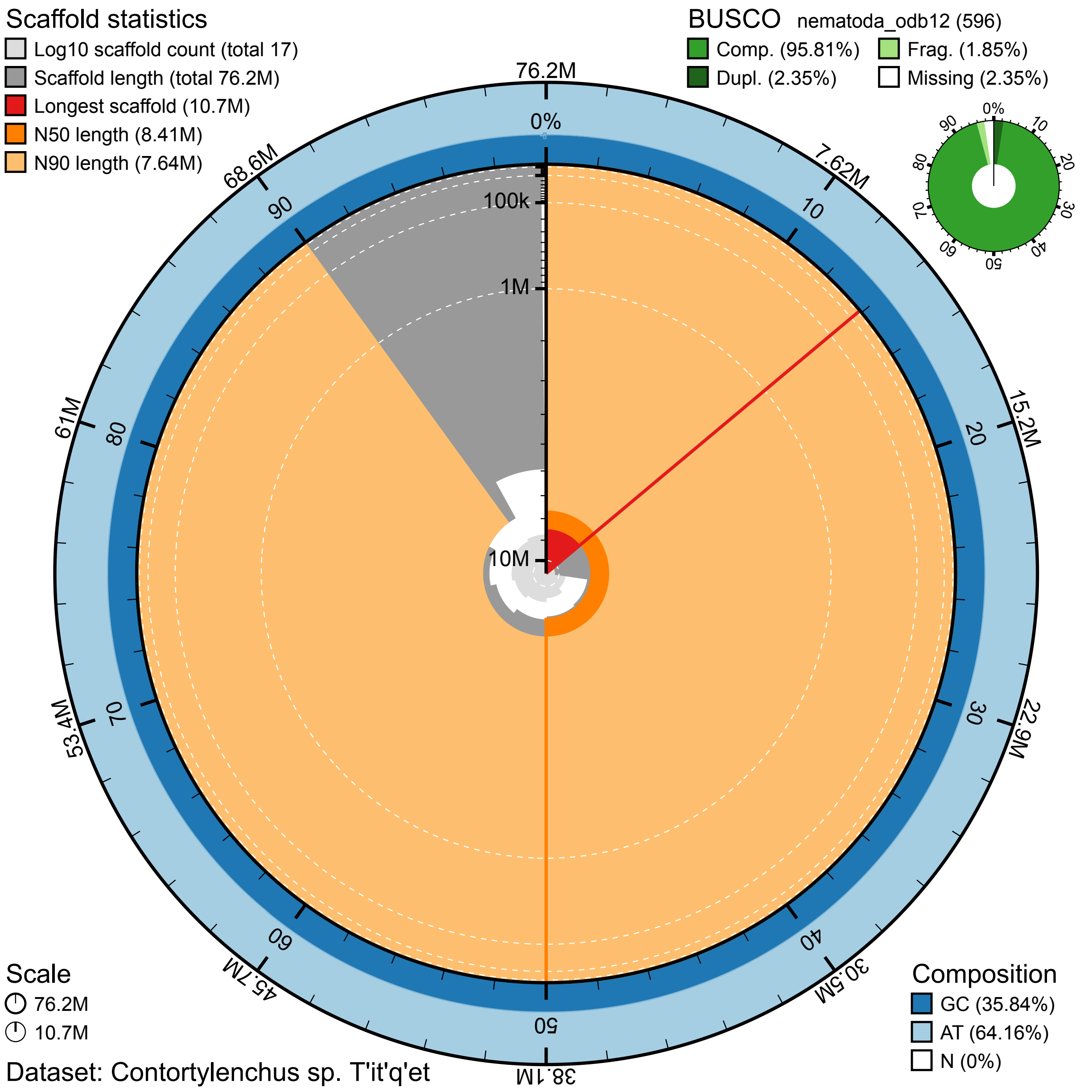

B

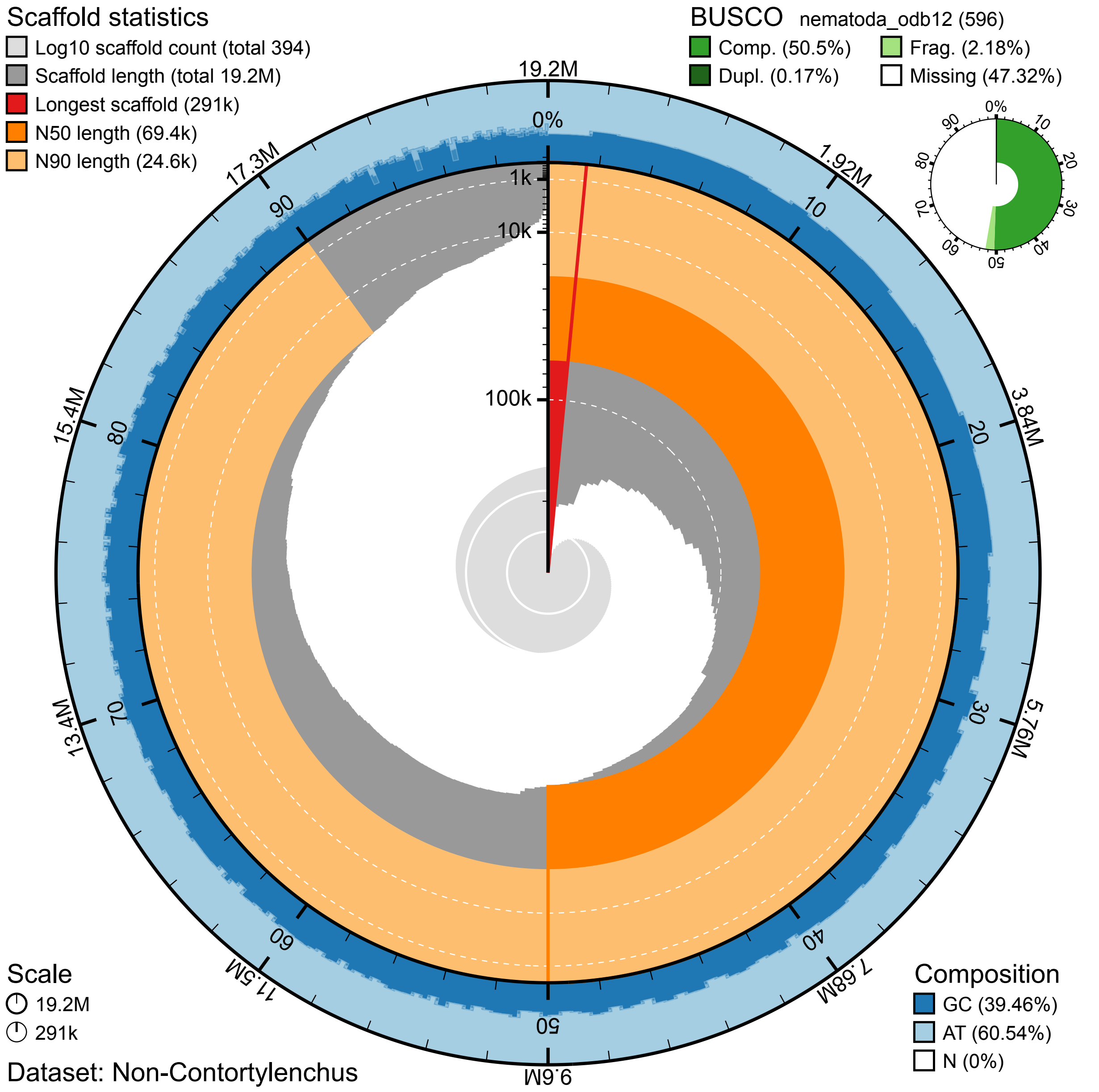

Supplement: Supplementary file 6 [file MEN-26-e70209-s009.pdf]
