# Supplementary material for: The Genome and Population Structure of the Spruce Beetle, Dendroctonus rufipennis (Kirby, 1837), a Transcontinental Pest of North American Spruce
Source: Mol Ecol Resour. 2026 Oct 29;26(8):e70209. doi: 10.1111/1755-0998.70209 (PMC13717379; doi:10.1111/1755-0998.70209)
Supplement: Supplementary file 7 [file MEN-26-e70209-s010.pdf]

Figure S1: Genome assembly schematic

Workflow used to assemble and annotate the genome. Each step is indicated in blue, with the software used shown in italics. The data used at each step are shown in red (with NCBI sample and sequence accessions, if applicable). See main text for further details.

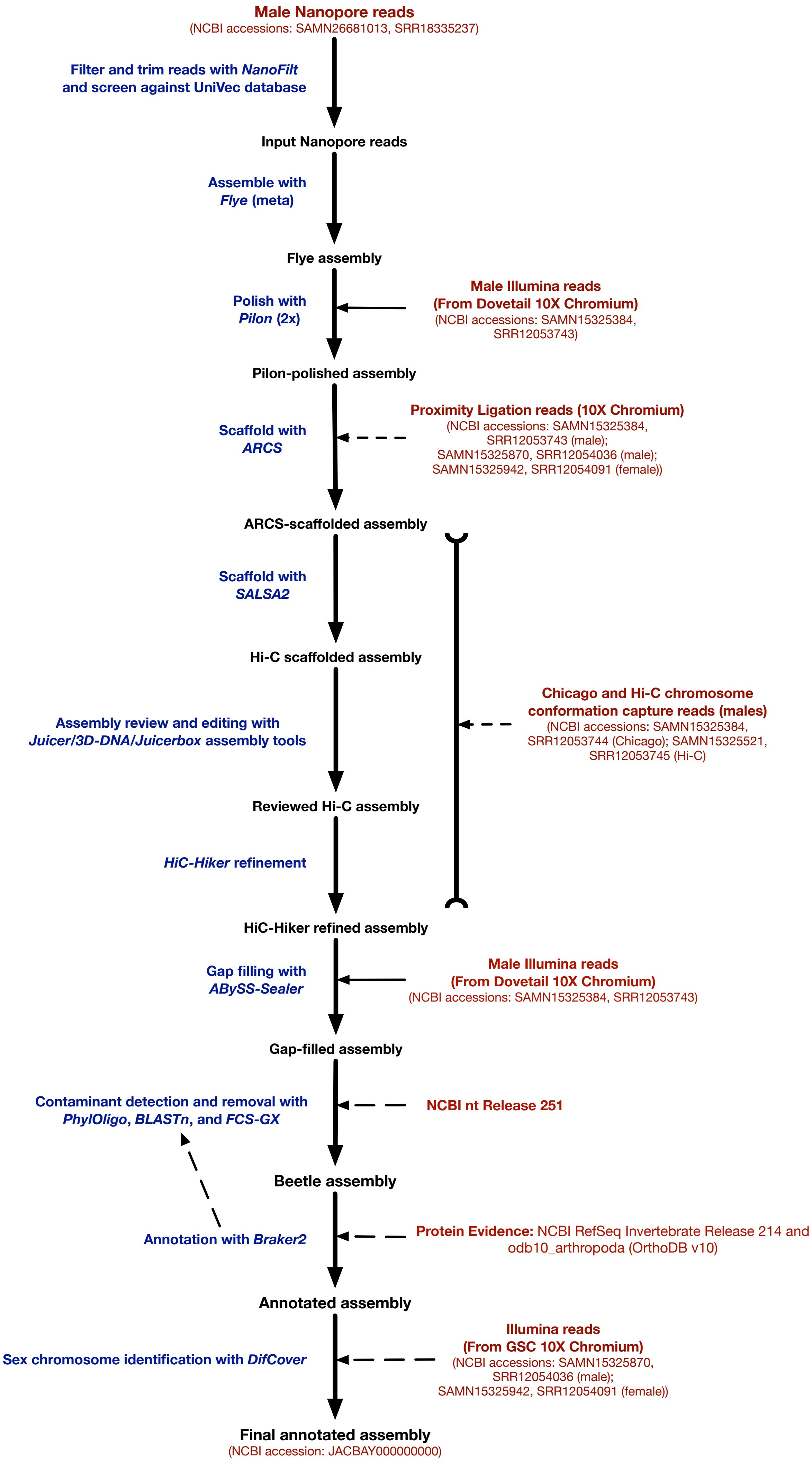

NCBI accession: SAMN31351181 is the combination of SAMN15325384 and SAMN26681013 as they both contribute to the final consensus sequence
