# Supplementary material for: The Genome and Population Structure of the Spruce Beetle, Dendroctonus rufipennis (Kirby, 1837), a Transcontinental Pest of North American Spruce
Source: Mol Ecol Resour. 2026 Oct 29;26(8):e70209. doi: 10.1111/1755-0998.70209 (PMC13717379; doi:10.1111/1755-0998.70209)

Figure S9: Phylogeny of *18S* rRNA gene sequences in Tylenchomorpha

We aligned the non-redundant nematode Tylenchomorpha *18S* rRNA gene sequences from NemaBase with our three nematode *18S* sequences (in red) using *mafft* and prepared a maximum-likelihood phylogeny with *FastTree2*. The circle diameter and colour at each node indicates the relative Shimodaira-Hasegawa-like local support value (bootstrap, between 0-1). We used *FigTree* to visualize the tree. We used the *18S* sequence from *Panagrolaimus davidi* (from Panagrolaimomorpha, in blue) as an outgroup to root the tree.

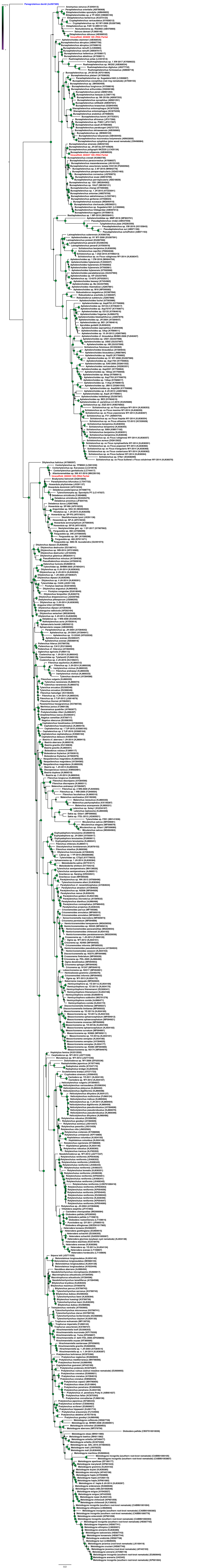

Supplement: Supplementary file 8 [file MEN-26-e70209-s011.pdf]
