# Supplementary material for: The Genome and Population Structure of the Spruce Beetle, Dendroctonus rufipennis (Kirby, 1837), a Transcontinental Pest of North American Spruce
Source: Mol Ecol Resour. 2026 Oct 29;26(8):e70209. doi: 10.1111/1755-0998.70209 (PMC13717379; doi:10.1111/1755-0998.70209)

Figure S18: Pair-wise shared synteny between spruce beetle and red turpentine beetle, mountain pine beetle, and southern pine beetle genomes

(A) Shared synteny between *D. rufipennis* (this study) and *D. ponderosae* (Keeling et al., 2022); (B) *D. rufipennis* and *D. frontalis* (Copeland et al., 2024); and (C) *D. rufipennis* and *D. valens* (Liu et al., 2022) assemblies.

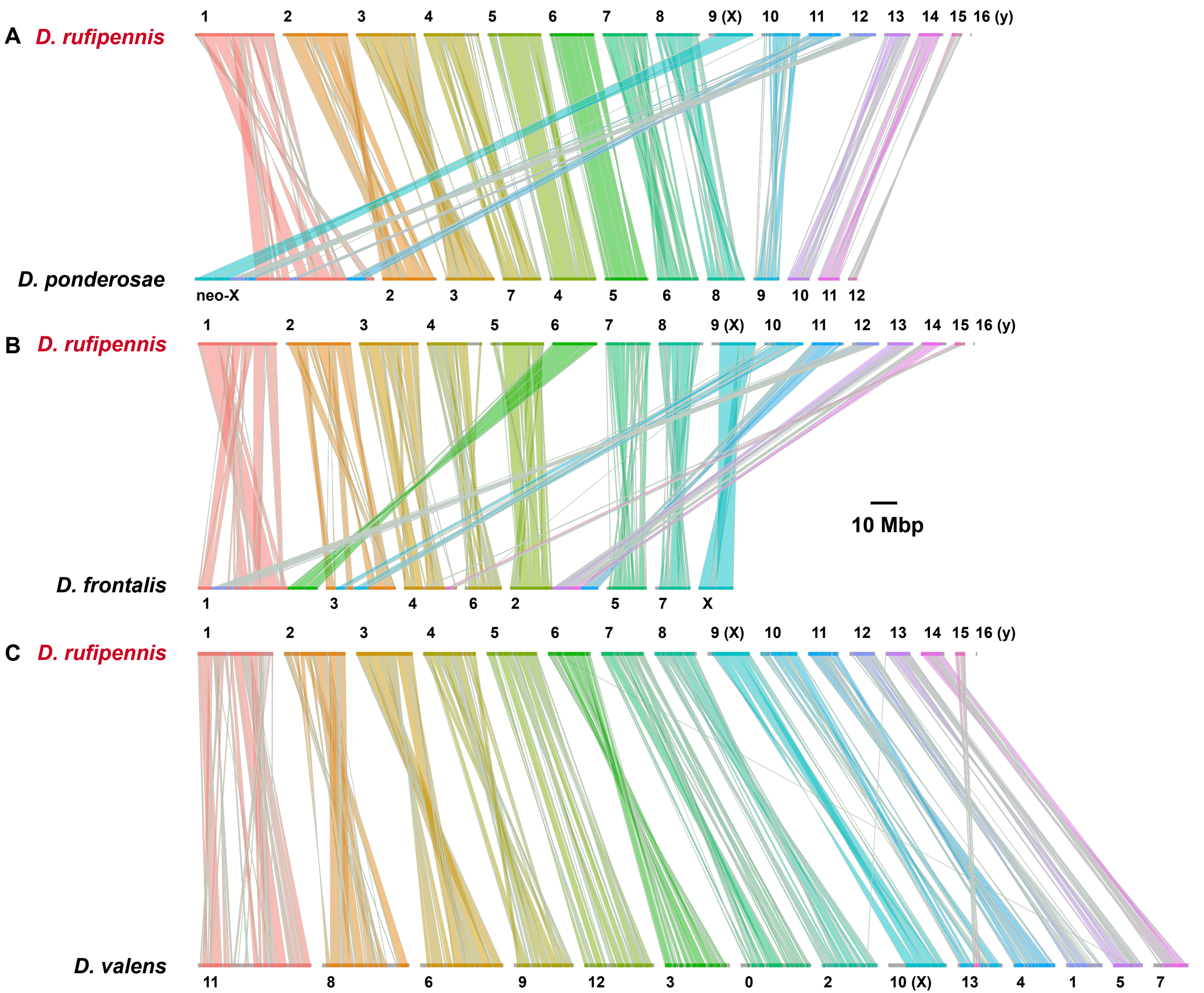

Supplement: Supplementary file 9 [file MEN-26-e70209-s013.pdf]
