# Supplementary material for: The Genome and Population Structure of the Spruce Beetle, Dendroctonus rufipennis (Kirby, 1837), a Transcontinental Pest of North American Spruce
Source: Mol Ecol Resour. 2026 Oct 29;26(8):e70209. doi: 10.1111/1755-0998.70209 (PMC13717379; doi:10.1111/1755-0998.70209)

Figure S15: *ADMIXTURE* cross-validation and additional *K* value clustering
(A) Cross-validation results for *ADMIXTURE* analysis indicate *K* = 3 has the lowest CV error, while *K* = 4 and 5 were similarly supported. Panel (B) shows all three of these *K* values, and demonstrates that even at *K* = 5 the four major groupings hold together. At *K* = 5 Utah demonstrates marginal distinction from Colorado and New Mexico, yet the Nova Scotia and Alaska population hold together despite large geographic distances between these sampling localities.

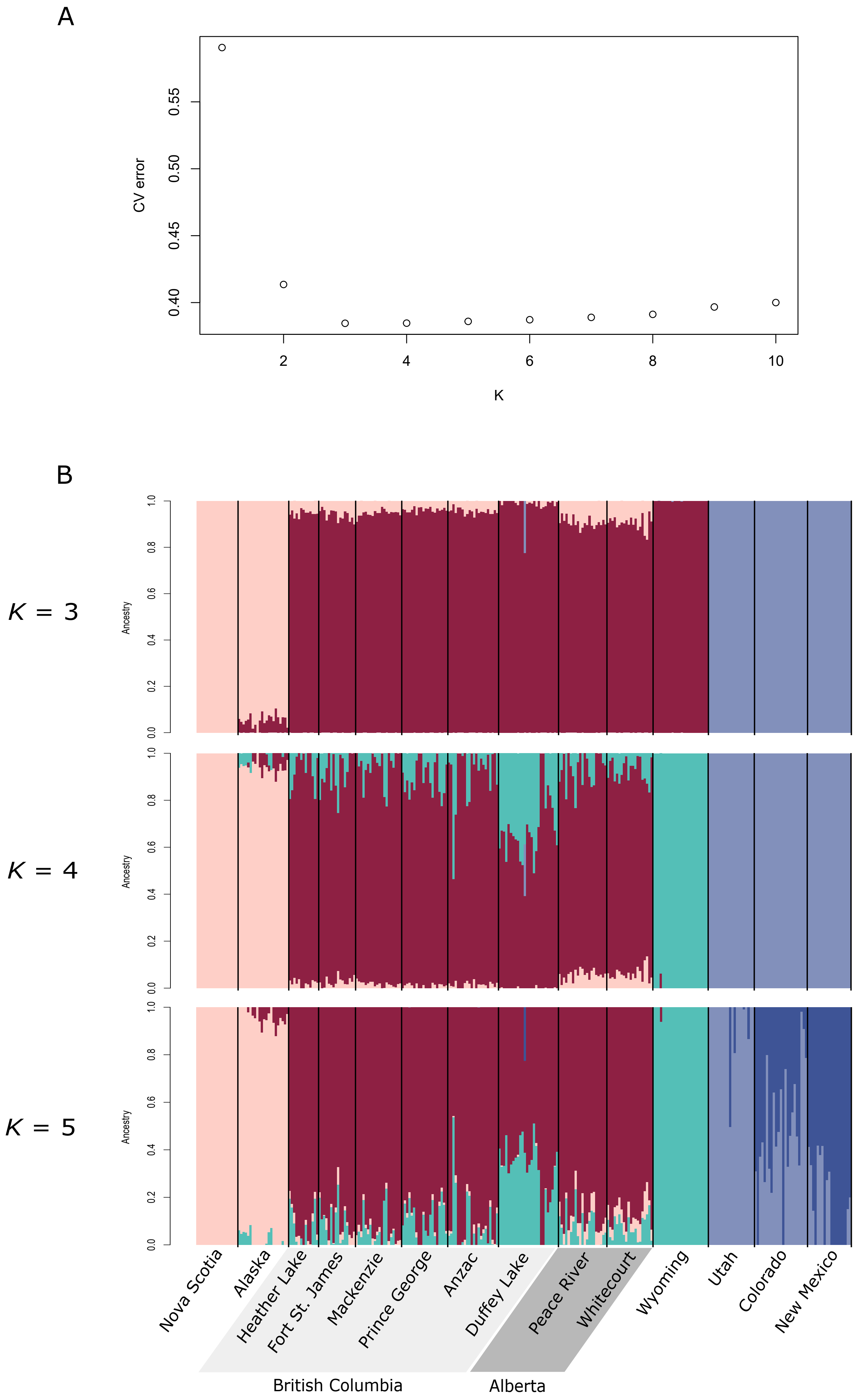

Supplement: Supplementary file 10 [file MEN-26-e70209-s014.pdf]
