# Supplementary material for: The Genome and Population Structure of the Spruce Beetle, Dendroctonus rufipennis (Kirby, 1837), a Transcontinental Pest of North American Spruce
Source: Mol Ecol Resour. 2026 Oct 29;26(8):e70209. doi: 10.1111/1755-0998.70209 (PMC13717379; doi:10.1111/1755-0998.70209)
Supplement: Supplementary file 11 [file MEN-26-e70209-s015.pdf]

Figure S5: Genome size estimation based on *k*-mer counts

(A) *GenomeScope 2.0* predictions of genome haploid length based on *k*-mer counts calculated by *FastK*; (B) *GenomeScope 2.0* outputs where *k* = 31.

A

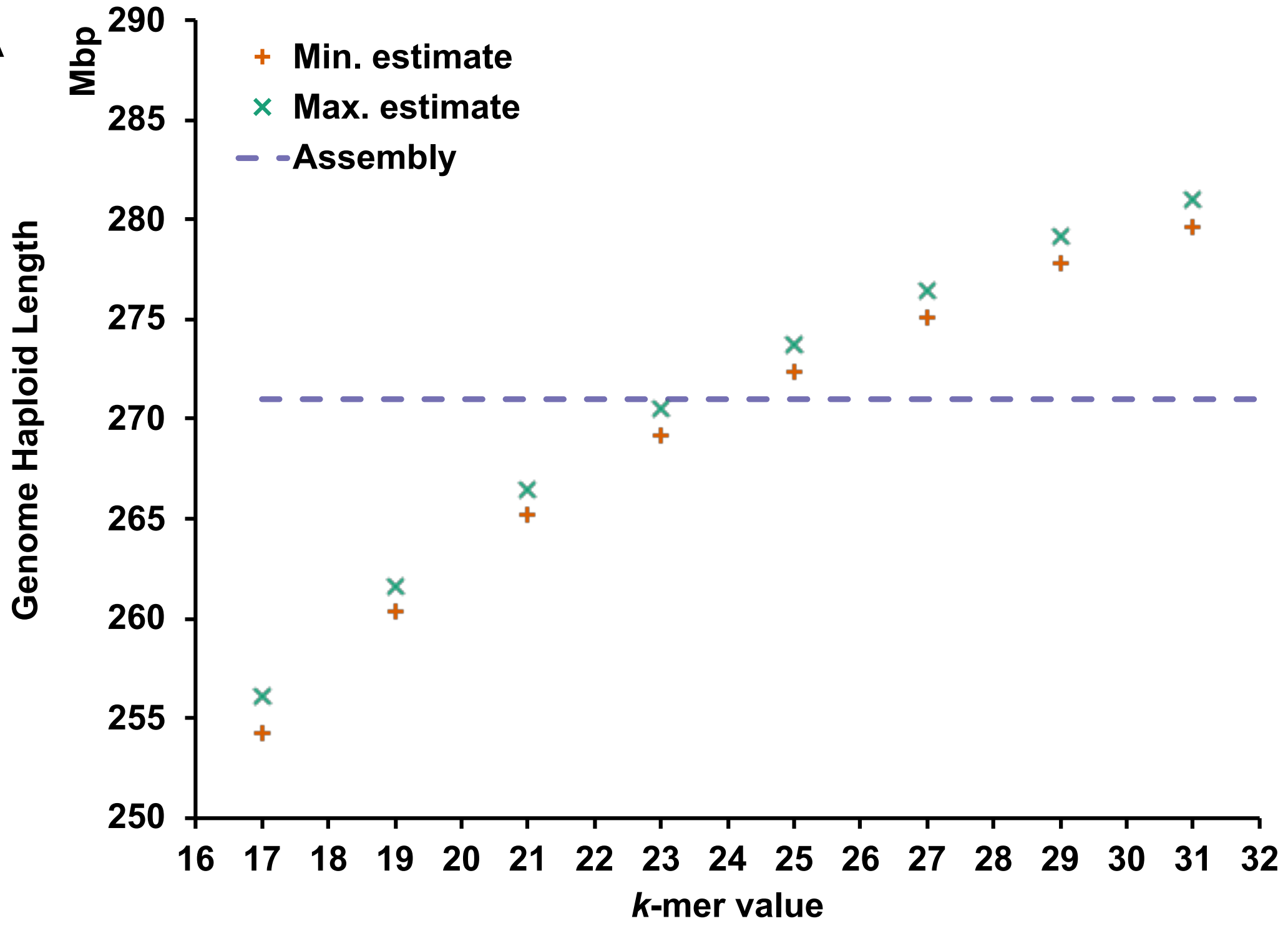

B

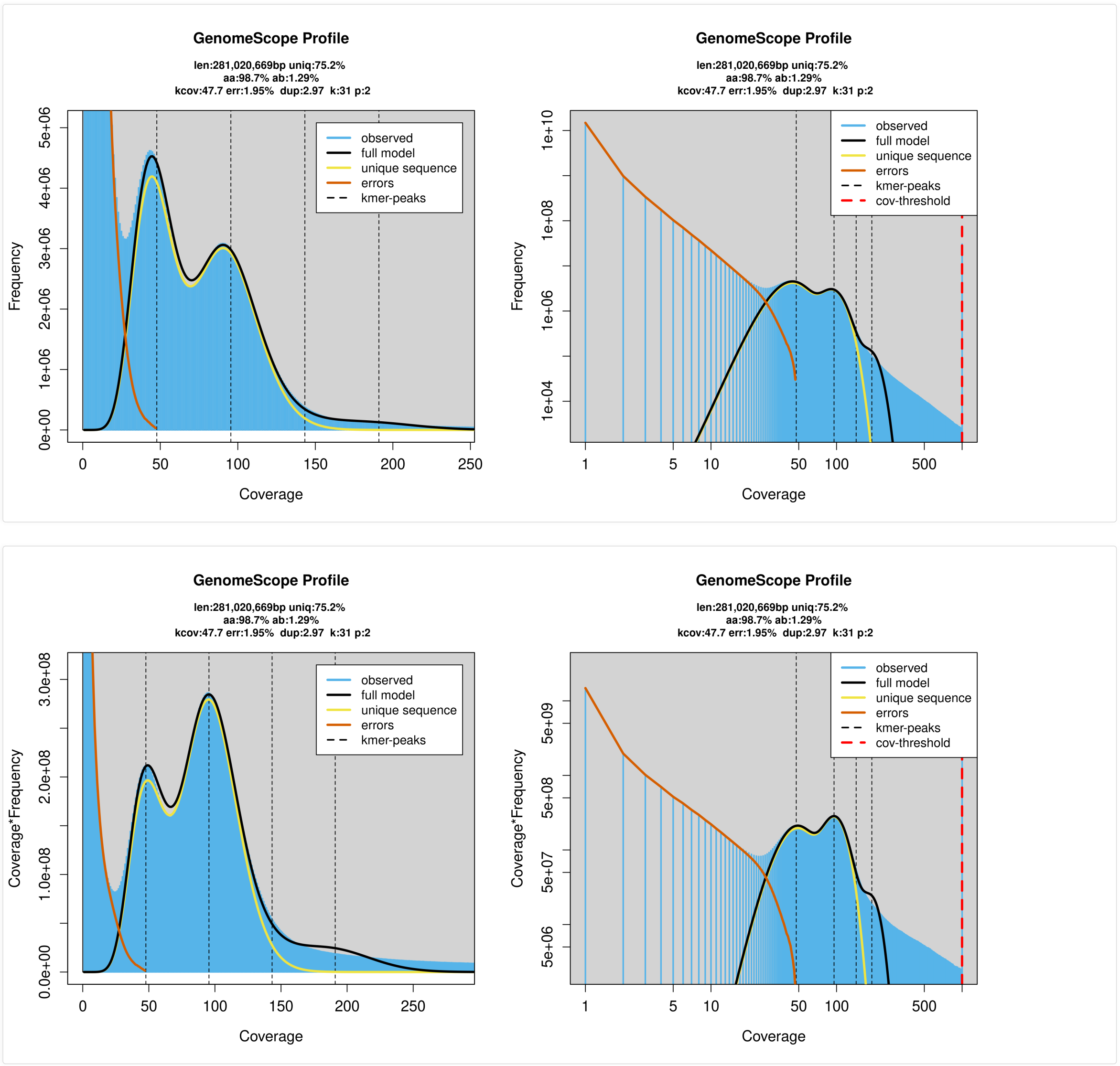

Results

```
GenomeScope version 2.0
input file = user_uploads/ZjazCFev5QCKOUlXt8jM
output directory = user_data/ZjazCFev5QCKOUlXt8jM
p = 2
k = 31

property                      min               max
Homozygous (aa)               98.6939%          98.7242%
Heterozygous (ab)             1.27582%          1.30606%
Genome Haploid Length         279,664,064 bp    281,020,669 bp
Genome Repeat Length          69,235,637 bp     69,571,488 bp
Genome Unique Length          210,428,427 bp    211,449,181 bp
Model Fit                     82.2758%          96.5996%
Read Error Rate               1.95177%          1.95177%
```

Model

```
Formula: y_transform ~ x^transform_exp * length * predict2_0(r1, k, d,
    kmercov, bias, x)

Parameters:
         Estimate Std. Error t value Pr(>|t|)
d       7.421e-02  1.929e-03   38.48   <2e-16 ***
r1      1.291e-02  7.560e-05  170.76   <2e-16 ***
kmercov 4.775e+01  5.777e-02  826.60   <2e-16 ***
bias    2.970e+00  3.282e-02   90.52   <2e-16 ***
length  2.278e+08  6.601e+05  345.15   <2e-16 ***
---
Signif. codes:  0 '***' 0.001 '**' 0.01 '*' 0.05 '.' 0.1 ' ' 1

Residual standard error: 5355000 on 965 degrees of freedom

Number of iterations to convergence: 7
Achieved convergence tolerance: 1.49e-08
```
