# Supplementary material for: The Genome and Population Structure of the Spruce Beetle, Dendroctonus rufipennis (Kirby, 1837), a Transcontinental Pest of North American Spruce
Source: Mol Ecol Resour. 2026 Oct 29;26(8):e70209. doi: 10.1111/1755-0998.70209 (PMC13717379; doi:10.1111/1755-0998.70209)

Figure S6: Sequencing coverage comparisons between male and female samples
The plot shows an equal normalized coverage for male and female samples across most scaffolds, but a two-fold higher coverage for female versus male for Druf_M_20221006_9, suggesting the X chromosome, and low female coverage for Druf_M_20221006_16, suggesting the Y chromosome. AC, Adjustment Coefficient = 1.331, a factor to correct for the difference in overall coverage between male and female samples (mode of 142 for female and 189 for male).

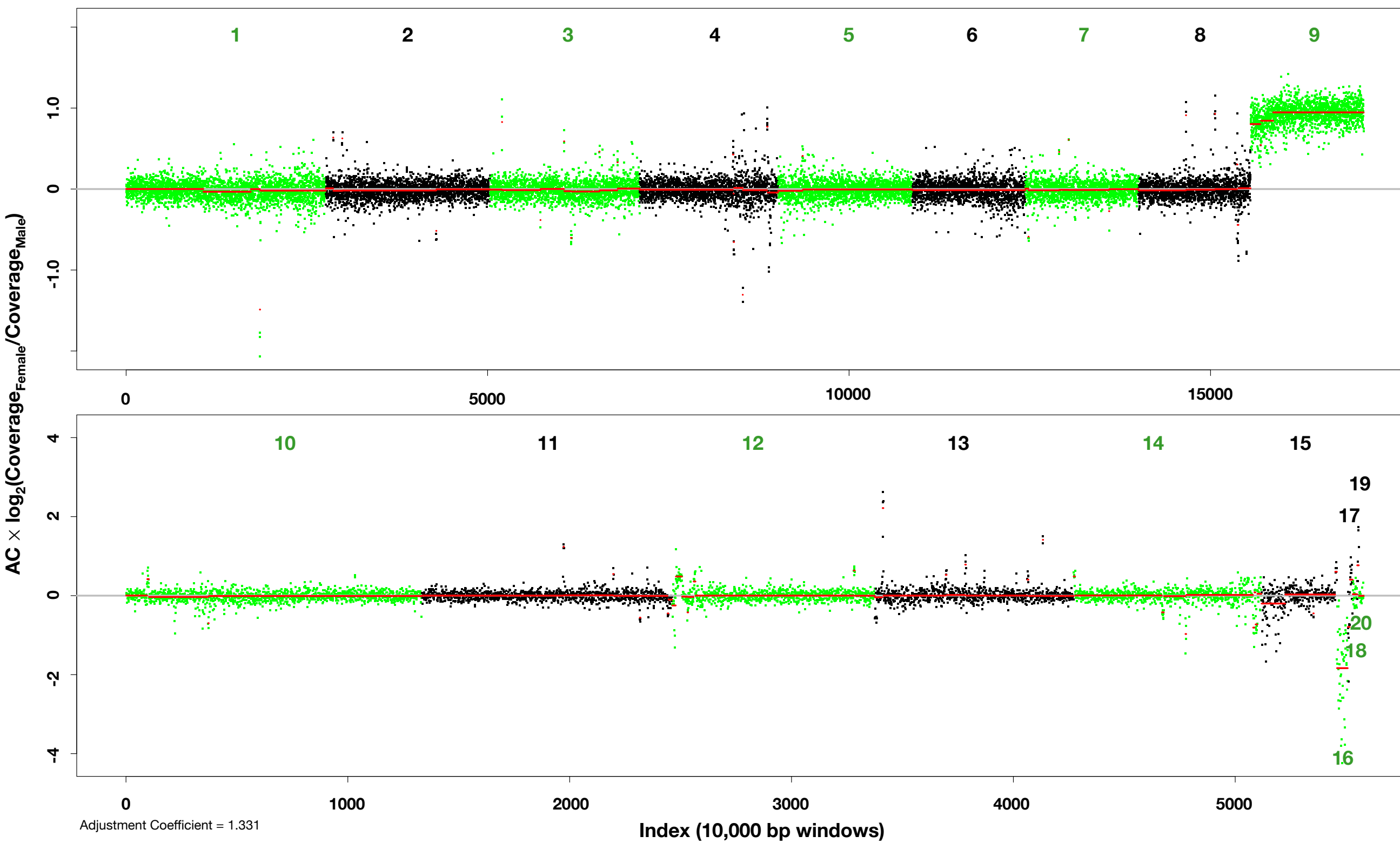

Supplement: Supplementary file 12 [file MEN-26-e70209-s016.pdf]
