# Supplementary material for: The Genome and Population Structure of the Spruce Beetle, Dendroctonus rufipennis (Kirby, 1837), a Transcontinental Pest of North American Spruce
Source: Mol Ecol Resour. 2026 Oct 29;26(8):e70209. doi: 10.1111/1755-0998.70209 (PMC13717379; doi:10.1111/1755-0998.70209)

Figure S12: Species phylogeny of Nematoda

Species phylogeny of the phylum Nematoda based on separate phylogenies of single copy orthologous sequences from reference nematode genomes available from NCBI (231 species, representing 105 genera). We used *wASTRAL* to create the species phylogeny, and rooted the tree mid-branch between the main Enoplea and Chromadorea clusters. *Contortylenchus* sp. T'it'q'et is shown in red. The circle diameter and colour at each node indicates the relative Shimodaira-Hasegawa-like local support value (bootstrap, between 0-1). Species used in the phylogeny and their genome assembly accessions are shown in Supplementary File S5.

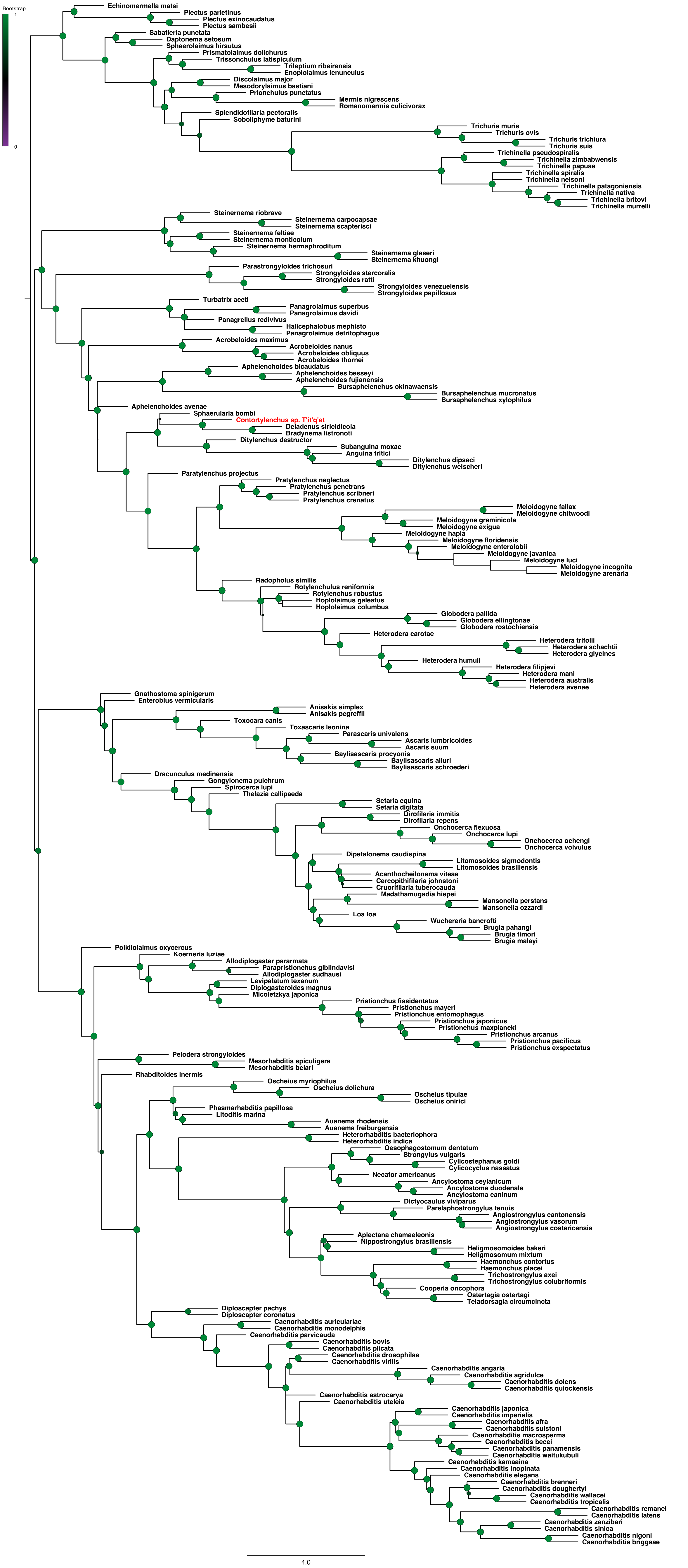

Supplement: Supplementary file 13 [file MEN-26-e70209-s017.pdf]
