# Supplementary material for: The Genome and Population Structure of the Spruce Beetle, Dendroctonus rufipennis (Kirby, 1837), a Transcontinental Pest of North American Spruce
Source: Mol Ecol Resour. 2026 Oct 29;26(8):e70209. doi: 10.1111/1755-0998.70209 (PMC13717379; doi:10.1111/1755-0998.70209)

Figure S20: Phylogeny of insect carboxylesterases and related enzymes

We aligned the manually curated and annotated carboxylesterases and related enzymes from the spruce beetle genome with representative Insecta protein sequences from the ESTHER database (ESTerases and alpha/beta-Hydrolase Enzymes and Relatives) using *mafft* and prepared a maximum-likelihood phylogeny with *FastTree2*. We used *FigTree* to visualize the unrooted tree. Druf_M_20221006_gNNNN indicate the spruce beetle gene models. Those ending with an m or A/B indicate a manually modified or split gene model from the automated annotations, respectively. Labels in red and orange indicate *Dendroctonus rufipennis* and other *Dendroctonus* spp., respectively. Other Coleoptera spp. are shown in blue. Species abbreviations (from ESTHER): acypi, *Acyrthosiphon pisum* (Pea aphid); aedae, *Aedes aegypti* (Yellowfever mosquito); anogl, *Anoplophora glabripennis* (Asian longhorn beetle); apime, *Apis mellifera* (Honeybee); bommo, *Bombyx mori* (Silk moth); denpd, *Dendroctonus ponderosae* (mountain pine beetle); drome, *Drosophila melanogaster* (Fruit fly); lepde, *Leptinotarsa decemlineata* (Colorado potato beetle); locmi, *Locusta migratoria* (Migratory locust); lyghe, *Lygus hesperus* (Western plant bug); pedhb, *Pediculus humanus corporis* (human body louse); pedhc, *Pediculus humanus capitis* (human head louse); solin, *Solenopsis invicta* (Red imported fire ant); spoli, *Spodoptera littoralis* (Egyptian cotton leafworm); tenmo, *Tenebrio molitor* (Yellow mealworm beetle); trica, *Tribolium castaneum* (Red flour beetle); and zoone, *Zootermopsis nevadensis* (Dampwood termite). The circle diameter and colour at each node indicates the relative Shimodaira-Hasegawa-like local support value (bootstrap, between 0-1). Classification Blocks C, H, L, X, their families, and some subfamilies are indicated. We rooted the tree to the midpoint between Block C and H, L, and X Blocks.

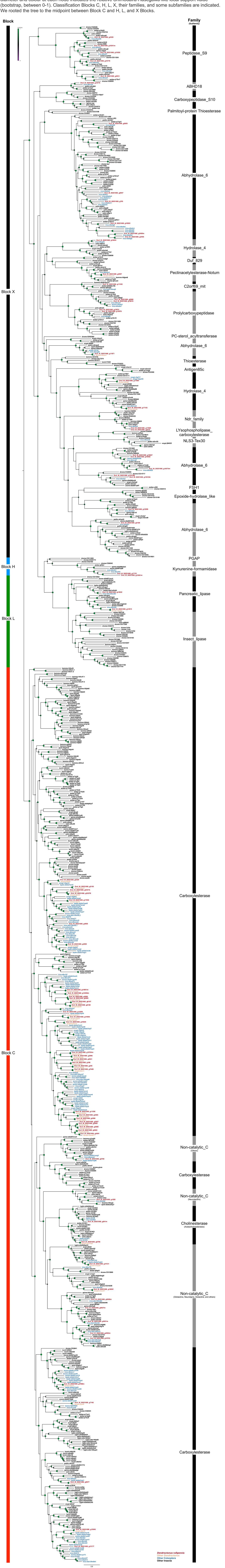

Supplement: Supplementary file 14 [file MEN-26-e70209-s018.pdf]
