# Supplementary material for: The Genome and Population Structure of the Spruce Beetle, Dendroctonus rufipennis (Kirby, 1837), a Transcontinental Pest of North American Spruce
Source: Mol Ecol Resour. 2026 Oct 29;26(8):e70209. doi: 10.1111/1755-0998.70209 (PMC13717379; doi:10.1111/1755-0998.70209)

Figure S16: F_{ST} and d_{xy} plotted by chromosome

Illustration of the relationship between F_{ST} and d_{xy} for the pairwise comparisons shown in the *OutFLANK* outlier analysis. Overall, d_{xy} does not appear to vary much across the genome, and F_{ST} islands of differentiation are not accompanied by obvious changes in d_{xy}.

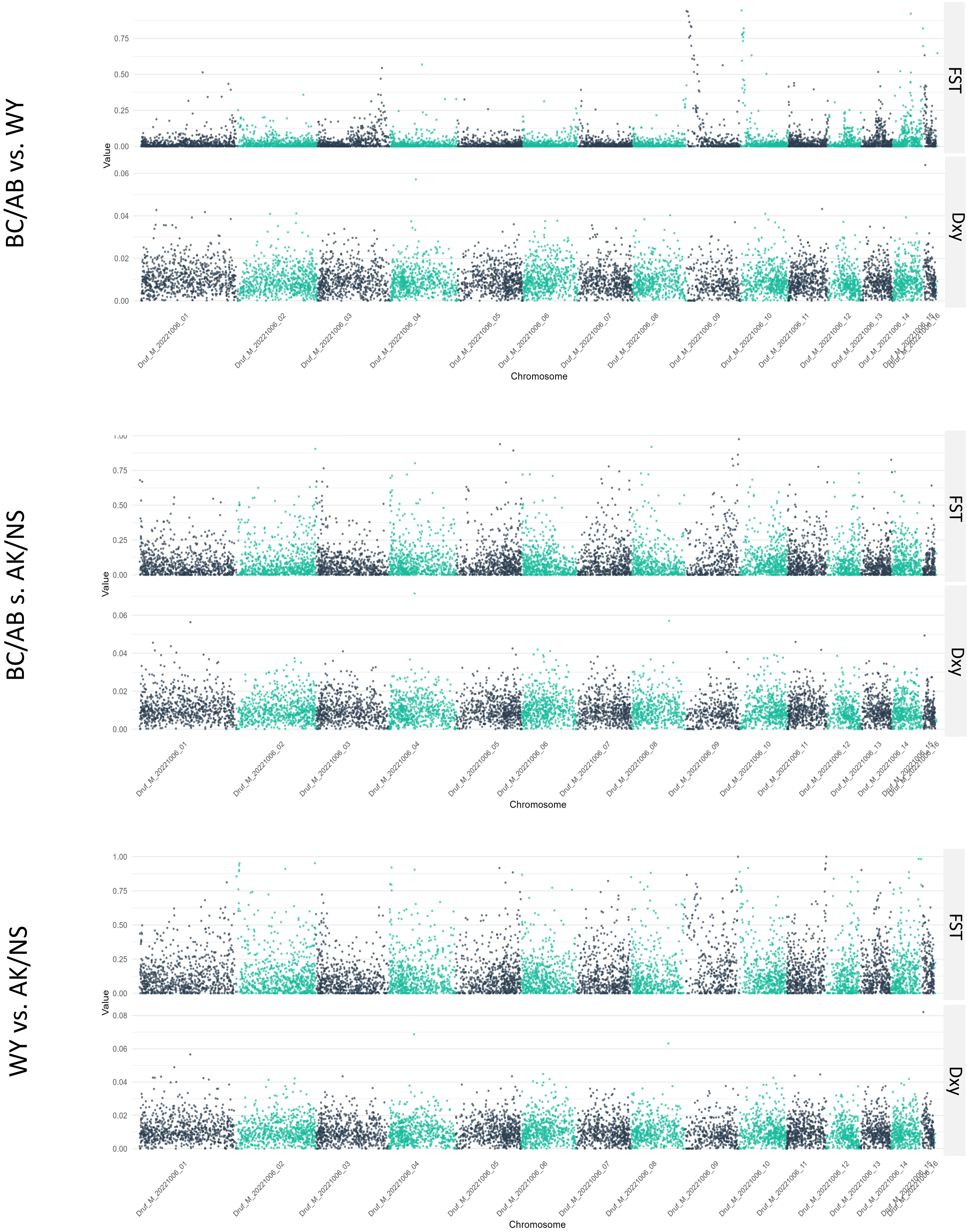

Supplement: Supplementary file 15 [file MEN-26-e70209-s019.pdf]
