# Supplementary material for: The Genome and Population Structure of the Spruce Beetle, Dendroctonus rufipennis (Kirby, 1837), a Transcontinental Pest of North American Spruce
Source: Mol Ecol Resour. 2026 Oct 29;26(8):e70209. doi: 10.1111/1755-0998.70209 (PMC13717379; doi:10.1111/1755-0998.70209)

Figure S8: Mitochondrion genome of *D. rufipennis*

The circular mitochondrial genome, annotated with protein-coding genes (13, shown in blue) and non-coding genes (22 tRNAs and 2 rRNAs, shown in red). The predicted anticodons are shown in brackets for the tRNAs. A 4,877 bp AT-rich region is shown in green, which contains two repeat regions, in pink: a 2,443 bp region with a 40 bp repeat and a 941 bp region with an imperfect 150 bp repeat.

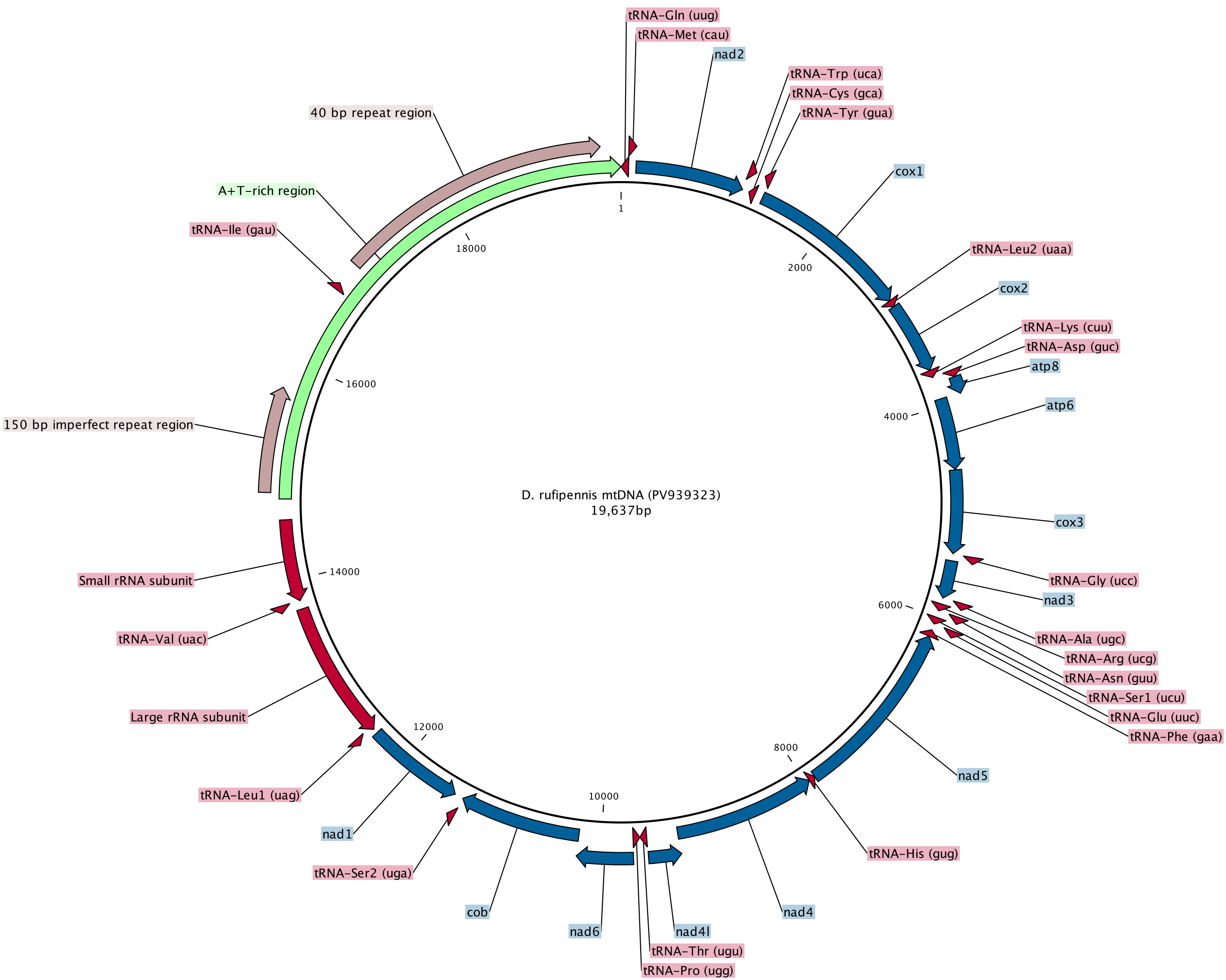

Supplement: Supplementary file 16 [file MEN-26-e70209-s021.pdf]
