# Supplementary material for: The Genome and Population Structure of the Spruce Beetle, Dendroctonus rufipennis (Kirby, 1837), a Transcontinental Pest of North American Spruce
Source: Mol Ecol Resour. 2026 Oct 29;26(8):e70209. doi: 10.1111/1755-0998.70209 (PMC13717379; doi:10.1111/1755-0998.70209)

Figure S3: Schematic of contamination identification using *PhylOligo* and *FCS-GX*

(A) Clusters generated by *hdbscan* with *PhylOligo* with decreasing minimum scaffold size; (B) Summary of main taxonomic grouping of each cluster from *PhylOligo* based on *BLASTn* searches against NCBI nt. Thick arrows link the same cluster members between scaffold sizes and thin arrows indicate a change of cluster membership for a few scaffolds; (C) *FCS-GX* identified 430 scaffolds as not being from *D. rufipennis* (taxid 77170) and indicated closest species matching in its dataset. The bar graph shows proportion of each *FCS-GX* taxonomic grouping in the 1 kbp+ *PhylOligo* cluster membership.

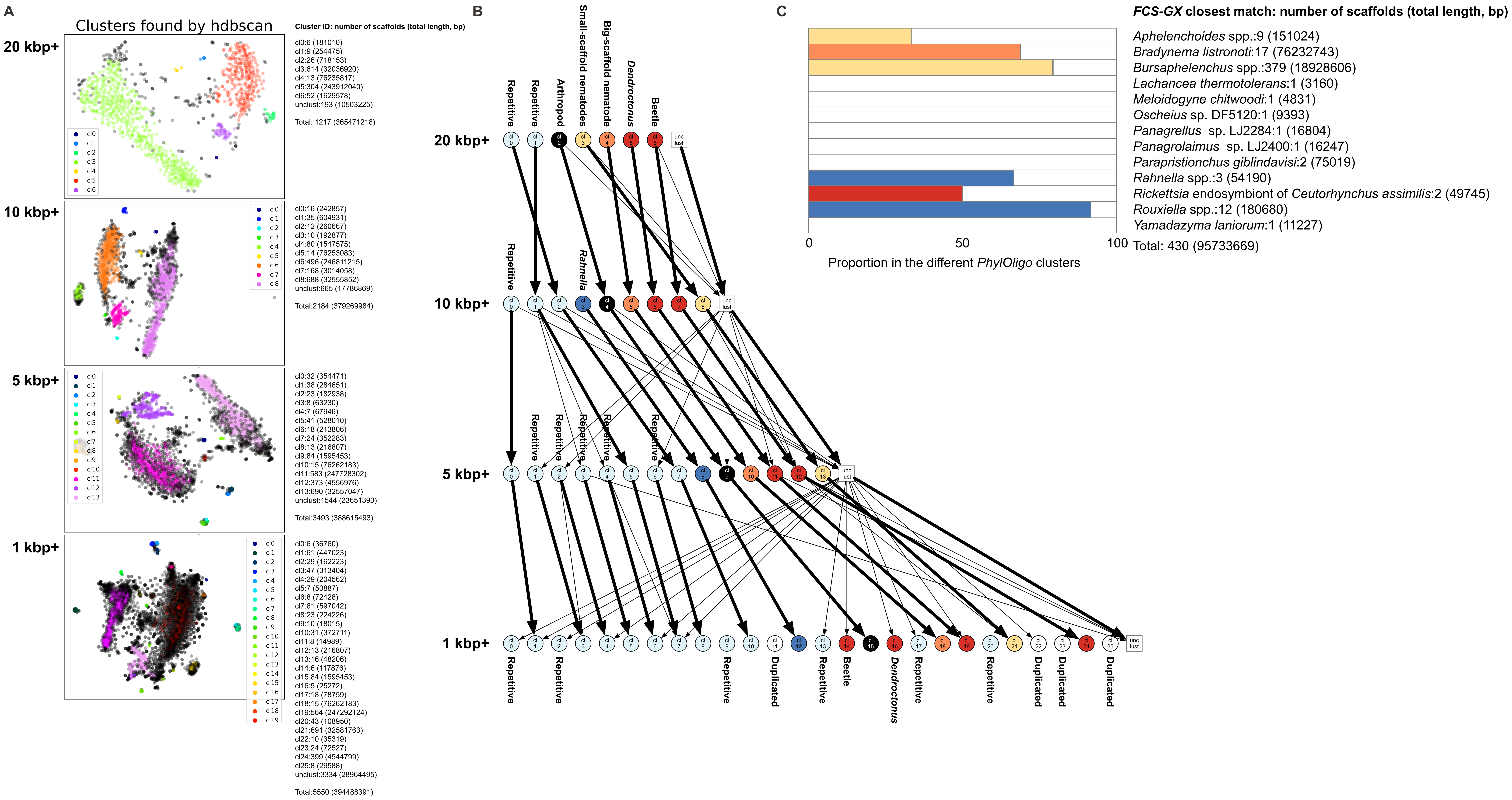

Supplement: Supplementary file 18 [file MEN-26-e70209-s024.pdf]
