# Supplementary material for: The Genome and Population Structure of the Spruce Beetle, Dendroctonus rufipennis (Kirby, 1837), a Transcontinental Pest of North American Spruce
Source: Mol Ecol Resour. 2026 Oct 29;26(8):e70209. doi: 10.1111/1755-0998.70209 (PMC13717379; doi:10.1111/1755-0998.70209)

Figure S14: Substructure analysis of spruce beetle genetic population clusters
(A) PCA of western Canada and Wyoming beetles shows differentiation of the Wyoming population; additional DAPC analysis of the western Canadian beetles only failed to resolve further substructure, as illustrated by the BIC plot in (B). Panel (C) illustrates marginal differentiation between specimens sampled in Utah from those sampled in Colorado and New Mexico, but DAPC did not support additional substructuring, as shown in the BIC plot in (D).

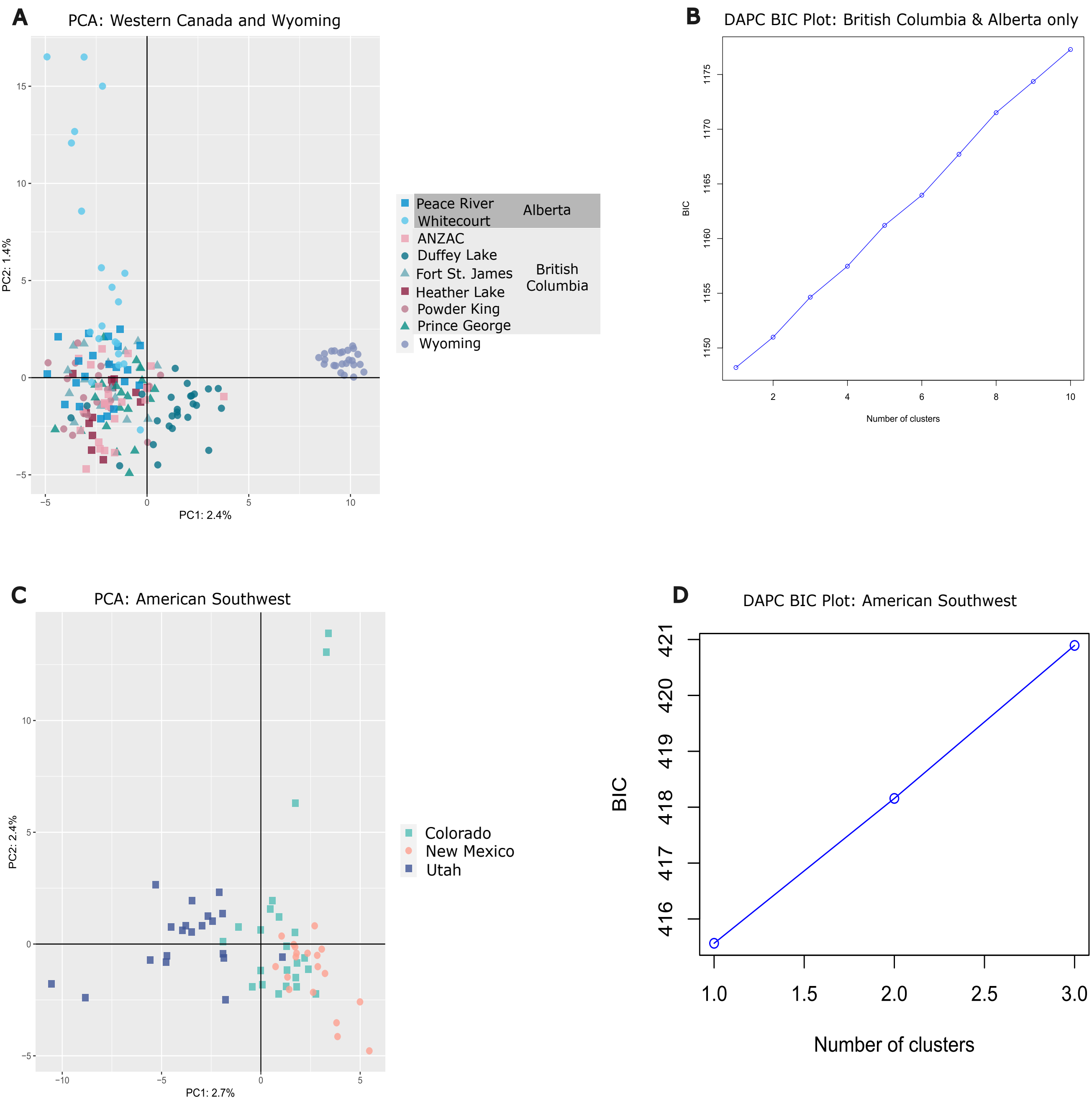

Supplement: Supplementary file 19 [file MEN-26-e70209-s025.pdf]
