# Supplementary material for: The Genome and Population Structure of the Spruce Beetle, Dendroctonus rufipennis (Kirby, 1837), a Transcontinental Pest of North American Spruce
Source: Mol Ecol Resour. 2026 Oct 29;26(8):e70209. doi: 10.1111/1755-0998.70209 (PMC13717379; doi:10.1111/1755-0998.70209)

Figure S19: Phylogeny of cytochrome P450 gene family

We aligned the manually curated and annotated P450s from the spruce beetle genome with representative Coleopteran P450 protein sequences using *MUSCLE* with manual refinement and prepared a maximum-likelihood phylogeny with *FastTree2*. We used *FigTree* to visualize the phylogeny. The circle diameter and colour at each node indicates the relative Shimodaira-Hasegawa-like local support value (bootstrap, between 0-1). Agra, *Anthonomus grandis* (Boll weevil); Darm, *Dendroctonus armandi* (Chinese white pine beetle); Dpon, *D. ponderosae* (Mountain pine beetle); Drhi, *D. rhizophagus*; Druf, *D. rufipennis* (Spruce beetle); Dval, *D. valens* (Red turpentine beetle); Efor, *Euwallacea fornicates* (Polyphagous shot-hole borer); Sory, *Sitophilus oryzae* (Rice weevil); Tcas, *Tribolium castaneum* (Red flour beetle). Human CYP3A4 was used as an outgroup to root the tree. GenBank accessions of P450s used in phylogeny are listed in Supplementary File S4.

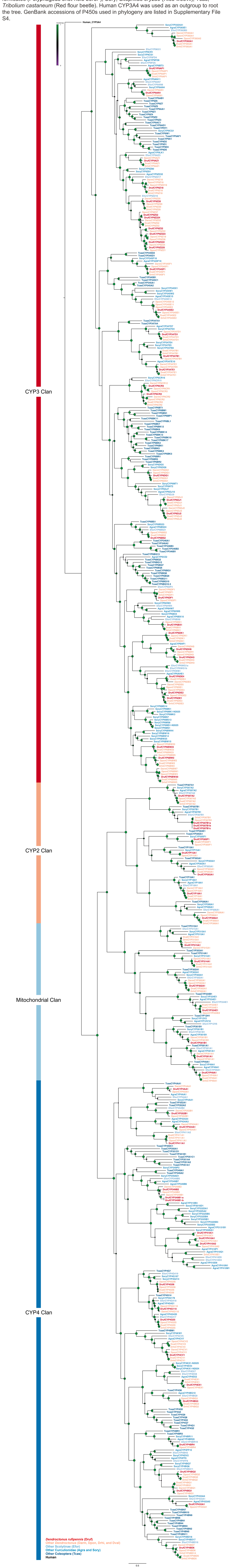

Supplement: Supplementary file 20 [file MEN-26-e70209-s026.pdf]
