# Supplementary material for: The Genome and Population Structure of the Spruce Beetle, Dendroctonus rufipennis (Kirby, 1837), a Transcontinental Pest of North American Spruce
Source: Mol Ecol Resour. 2026 Oct 29;26(8):e70209. doi: 10.1111/1755-0998.70209 (PMC13717379; doi:10.1111/1755-0998.70209)
Supplement: Supplementary file 21 [file MEN-26-e70209-s006.zip › Supplementary Information File S21.pdf]

MOLECULAR ECOLOGY RESOURCES

Supplementary Information for:

The genome and population structure of the spruce beetle, *Dendroctonus rufipennis* (Kirby, 1837), a transcontinental pest of North American spruce

Erin O. Campbell, Philip D. Batista, Véronique Lévesque-Tremblay, Catherine Béliveau, Kathia Bernier, Kateryna Romanenko, Ward B. Strong, Katherine P. Bleiker, Dezene P.W. Huber, and Christopher I. Keeling

Table of Contents:

**Supplementary Tables:**	**2**
**Table S1: Population genetics sample information**	**2**
**Table S2: Spruce beetle repetitive sequences**	**3**
**Table S3: *Contortylenchus* sp. T'it'q'et genome assembly statistics**	**5**
**Table S4: *Contortylenchus* sp. T'it'q'et repetitive sequences**	**6**
**Table S5: Average nucleotide diversity, pairwise d_{xy} (upper diagonal) and pairwise F_{ST} (lower diagonal) for all sampling localities**	**8**
**Table S6: Average nucleotide diversity, pairwise d_{xy} (upper diagonal) and pairwise F_{ST} (lower diagonal) for major genetic clusters**	**9**
**Table S7: *Dendroctonus* spp. RADseq accessions used in phylogeny**	**10**

Supplemental Tables

Table S1: Population genetics sample information

Collection details and sample coding for beetles used in population genetic analyses

Province/State	Locality	Sample Code	Collection date	Putative host	Lat	Long	N	Collected by:
British Columbia	Anzac	S2	2018-05-15	hybrid spruce	54.726277	-122.249006	22	Philip Batista, Jeanne Robert
British Columbia	Mackenzie	S3	2018-05-16	hybrid spruce	55.268107	-122.68583	20	Philip Batista, Jeanne Robert
British Columbia	Heather Lake	S4	2018-05-17	hybrid spruce	55.554303	-123.323083	13	Philip Batista, Jeanne Robert
British Columbia	Fort St James	FJ / S7 / S8	2018-08-30	hybrid spruce	54.165375	-123.956176	24	Cindy Holland
British Columbia	Duffey Lake	DL / DL2	2018-09-28	hybrid spruce	50.37817	-122.34263	21	Katherine Bleiker, Telsa Willsey
British Columbia	Prince George	PG	2018-09-29	hybrid spruce	54.62608	-122.3017	20	Katherine Bleiker, Telsa Willsey
Alberta	Peace River	S9	2018-12-19	black spruce	56.729167	-118.282783	22	Ryan Hermanutz, Matthew Gelderman
Alberta	Whitecourt	WC	2018-09-30	black spruce	54.09703	-116.06283	20	Katherine Bleiker, Telsa Willsey
Nova Scotia		NS	2019-05-31	red spruce	45.361875	-64.7246089	18	Celia Boone
Utah		UT	2018-09-20	Englemann spruce	40.58585	-110.431667	21	Matt Hansen
Colorado		CO / G	2018-09-27	Englemann spruce	39.606267	-105.729183	20	Matt Hansen
Wyoming		WY	2018-09-19	Englemann spruce	43.808417	-110.221517	23	Steve Munson, Chris Fettig
New Mexico		NM	2018-09-11	Englemann spruce	36.681067	-106.2114	20	Andrew Graves
Alaska	Denali State Park	AZ	2018-11-11	black spruce	62.6836	-150.241	22	Jason Moan

Table S2: Spruce beetle repetitive sequences
Summary of repetitive sequences identified by *Repeatmasker* in the *D. rufipennis* genome.

Assembly name: Druf_M_20221006.4.fa
Number of sequences: 3571
Total length: 270673615 bp (270618515 bp excl N/X-runs)
GC level: 35.73 %
Bases masked: 105110034 bp (38.83 %)

	Number of Elements*	Length occupied (bp)	Percentage of sequence (%)
**Retroelements**	60474	39214338	14.49
SINEs:	209	43456	0.02
Penelope	517	150596	0.06
LINEs:	43395	23915358	8.84
CRE/SLACS	0	0	0
L2/CR1/Rex	5726	2887802	1.07
R1/LOA/Jockey	5843	2176396	0.8
R2/R4/NeSL	162	209401	0.08
RTE/Bov-B	12912	6459761	2.39
L1/CIN4	0	0	0
LTR elements:	16353	15104928	5.58
BEL/Pao	3294	7054131	2.61
Ty1/Copia	2863	1470349	0.54
Gypsy/DIRS1	7245	5876706	2.17
Retroviral	115	35419	0.01
**DNA transposons**	51853	19631641	7.25
hobo-Activator	7533	3721217	1.37
Tc1-IS630-Pogo	35440	11228129	4.15
En-Spm	0	0	0
MULE-MuDR	827	423719	0.16
PiggyBac	599	750481	0.28
Tourist/Harbinger	2110	909778	0.34
Other (Mirage, P-element, Transib)	1739	663088	0.24
**Rolling-circles**	3076	1473445	0.54
**Unclassified:**	143456	43540067	16.09

**Total interspersed repeats:**		102386046	37.83
**Small RNA:**	77	7884	1.97
**Satellites:**	70	12157	0
**Simple repeats:**	23497	1013010	0.37
**Low complexity:**	4905	225376	0.08

* Most repeats fragmented by insertions or deletions have been counted as one element

Prepared after *RepeatModeler* (v2.0.7) with *RepeatMasker* (v4.2, default mode) and associated dependencies within the *Dfam TE Tools Container* (v1.93, Dfam Consortium, 2025)

run with *rmblastn*
version 2.14.1+
The query was compared to classified sequences in
"assembly-families.fa"
FamDB:

Table S3: *Contortylenchus* sp. T'it'q'et genome assembly statistics

Parameter	Value
Overall Assembly	
Assembly size (Mb)	76.2
GC content (%)	35.8
Repetitive sequences (%)[1]	15.4
BUSCO completeness of assembly (%)[2]	95.8
Scaffolds	
Number of scaffolds	17
Longest scaffold (Mb)	10.7
N50 scaffold length (Mb)	8.4
L50 scaffold count	5
Contigs	
Number of contigs	31
Longest contig (Mb)	10.1
N50 contig length (Mb)	4.6
L50 contig count	6
Number of contigs in scaffolds	22
Annotation	
Predicted gene models	12,208
Predicted transcripts	13,189
BUSCO completeness of annotation (%)[2]	94.8

Table S4 *Contortylenchus* sp. T'it'q'et repetitive sequences
Summary of repetitive sequences identified by *Repeatmasker* in the *Contortylenchus* sp. T'it'q'et genome.

Assembly name: Contortylenchus_sp_CFS_20250926.0.fa
Number of sequences: 17
Total length: 76232743 bp (76231343 bp excl N/X-runs)
GC level: 35.84 %
Bases masked: 11748338 bp (15.41 %)

	Number of Elements*	Length occupied (bp)	Percentage of sequence (%)
**Retroelements**	2088	417733	0.55
SINEs:	184	35659	0.05
Penelope	0	0	0
LINEs:	1557	220925	0.29
CRE/SLACS	0	0	0
L2/CR1/Rex	421	87659	0.11
R1/LOA/Jockey	0	0	0
R2/R4/NeSL	0	0	0
RTE/Bov-B	0	0	0
L1/CIN4	1136	133266	0.17
LTR elements:	347	161149	0.21
BEL/Pao	118	57463	0.08
Ty1/Copia	0	0	0
Gypsy/DIRS1	134	86926	0.11
Retroviral	0	0	0
**DNA transposons**	10070	3150602	4.13
hobo-Activator	279	76733	0.1
Tc1-IS630-Pogo	7272	2449687	3.21
En-Spm	0	0	0
MULE-MuDR	0	0	0
PiggyBac	0	0	0
Tourist/Harbinger	0	0	0
Other (Mirage, P-element, Transib)	0	0	0
**Rolling-circles**	87	48278	0.06
**Unclassified:**	37616	6170290	8.09

**Total interspersed repeats:**		9738625	12.77
**Small RNA:**	300	47237	0.06
**Satellites:**	37	65348	0.09
**Simple repeats:**	30975	1502467	1.97
**Low complexity:**	7112	370563	0.49

* Most repeats fragmented by insertions or deletions have been counted as one element

Prepared after *RepeatModeler* (v2.0.7) with *RepeatMasker* (v4.2, default mode) and associated dependencies within the *Dfam TE Tools Container* (v1.93, Dfam Consortium, 2025)

run with *rmblastn* version
2.14.1+
The query was compared to classified sequences in
"assembly-families.fa"
FamDB:

Table S5: Average nucleotide diversity, pairwise d_{xy} (upper diagonal) and pairwise F_{ST} (lower diagonal) for all sampling localities.

				British Columbia						Alberta						
		Nucleotide diversity (π)	AK	Heather Lake	Fort St. James	Mackenzie	Prince George	Anzac	Duffey Lake	Peace River	Whitecourt	WY	UT	CO	NM	NS
	AK	0.00843	--	0.011	0.010	0.011	0.010	0.010	0.010	0.010	0.010	0.010	0.013	0.013	0.013	0.009
British Columbia	Heather Lake	0.01020	0.13	--	0.010	0.010	0.010	0.010	0.010	0.010	0.009	0.010	0.013	0.013	0.012	0.011
	Fort St. James	0.00997	0.12	0.00	--	0.01	0.010	0.010	0.010	0.010	0.009	0.010	0.013	0.013	0.012	0.010
	Mackenzie	0.01010	0.12	0.00	0.00	--	0.010	0.010	0.010	0.010	0.009	0.010	0.013	0.013	0.012	0.010
	Prince George	0.00907	0.13	0.01	0.01	0.01	--	0.009	0.009	0.009	0.009	0.009	0.013	0.012	0.012	0.010
	Anzac	0.00970	0.12	0.00	0.00	0.00	0.01	--	0.009	0.009	0.009	0.010	0.013	0.013	0.012	0.010
	Duffey Lake	0.00920	0.14	0.01	0.01	0.01	0.01	0.01	--	0.009	0.009	0.009	0.013	0.012	0.012	0.010
Alberta	Peace River	0.00923	0.12	0.01	0.00	0.01	0.01	0.01	0.01	--	0.009	0.009	0.013	0.012	0.012	0.010
	Whitecourt	0.00851	0.13	0.02	0.02	0.02	0.02	0.02	0.02	0.01	--	0.009	0.012	0.012	0.012	0.010
	WY	0.00913	0.17	0.04	0.04	0.04	0.04	0.03	0.03	0.04	0.05	--	0.013	0.012	0.012	0.010
	UT	0.00606	0.46	0.40	0.39	0.39	0.40	0.38	0.38	0.39	0.40	0.39	--	0.006	0.006	0.013
	CO	0.00593	0.45	0.40	0.39	0.38	0.39	0.38	0.38	0.39	0.40	0.39	0.01	--	0.006	0.013
	NM	0.00543	0.44	0.38	0.38	0.37	0.38	0.37	0.37	0.38	0.39	0.38	0.02	0.01	--	0.013
	NS	0.00727	0.09	0.18	0.17	0.17	0.18	0.17	0.19	0.16	0.17	0.21	0.50	0.50	0.49	--

Table S6: Average nucleotide diversity, pairwise d_{xy} (upper diagonal) and pairwise F_{ST} (lower diagonal) for major genetic clusters

	Nucleotide diversity (π)	AK/NS	BC/AB	WY	UT/CO/NM
AK/NS	0.00833	--	0.01	0.01	0.013
BC/AB	0.00956	0.13	--	0.01	0.013
WY	0.00913	0.17	0.03	--	0.012
UT/CO/NM	0.00587	0.46	0.36	0.41	--

Table S7: *Dendroctonus* spp. RADseq accessions used in phylogeny

Species	NCBI Accession*
D. micans	SRR8838392
D. micans	SRR8838393
D. murrayanae	SRR8838390
D. murrayanae	SRR8838391
D. pseudotsugae	SRR8838350
D. pseudotsugae	SRR8838351
D. punctatus	SRR8838383
D. punctatus	SRR8838384
D. simplex	SRR8838360
D. simplex	SRR8838361

*Part of NCBI BioProject PRJNA530572
