# Supplementary material for: The Genome and Population Structure of the Spruce Beetle, Dendroctonus rufipennis (Kirby, 1837), a Transcontinental Pest of North American Spruce
Source: Mol Ecol Resour. 2026 Oct 29;26(8):e70209. doi: 10.1111/1755-0998.70209 (PMC13717379; doi:10.1111/1755-0998.70209)
Supplement: Supplementary file 21 [file MEN-26-e70209-s006.zip › Supplementary Materials and Methods.pdf]

MOLECULAR ECOLOGY RESOURCES

Supplementary Materials and Methods for:

The genome and population structure of the spruce beetle, *Dendroctonus rufipennis* (Kirby, 1837), a transcontinental pest of North American spruce

Erin O. Campbell, Philip D. Batista, Véronique Lévesque-Tremblay, Catherine Béliveau, Kathia Bernier, Kateryna Romanenko, Ward B. Strong, Katherine P. Bleiker, Dezene P.W. Huber, and Christopher I. Keeling

Table of Contents:

Supplementary Materials and Methods:	2
Sources of beetles	2
DNA extraction methods	2
Genome sequencing library preparation and sequencing	4
Protocol to determine sex of *Dendroctonus rufipennis* by PCR	6
Annotation of cytochrome P450 and carboxyl/cholinesterase (CCE) gene families	10
Mitochondrial genome assembly and annotation	10
Identifying co-sequenced organisms and nematode genome analyses	10
References for Supplementary Materials and Methods	13

Supplementary Materials and Methods:

Sources of beetles

We collected adult spruce beetles for flow cytometry and Nanopore, 10x Chromium, Chicago, and Hi-C sequencing from infested trees at the sites and years stated below. The host trees at Duffey Lake (50.38° N, 122.34° W, elevation 1150 m) were Engelmann spruce or an Engelmann-Sitka spruce hybrid (*P. engelmannii* x *P. sitchensis)*. The host tree at Prince George site (54.33976° N, 122.2811° W, elevation 800 m) was an Engelmann-white spruce hybrid commonly referred to as interior hybrid spruce (*Picea engelmannii* Parry ex. Engelm × *P. glauca*). In each case, mature spruce trees appearing healthy were felled prior to beetle's dispersal flight in the region. Approximately one to two weeks after the flight, when adults were in the process of laying eggs in the felled trees, several 30 cm long logs were cut from the infested portion of boles. The logs were transported to the Pacific Forestry Centre in Victoria, BC and the cut ends covered with paraffin wax to reduce desiccation. Logs were held at 21°C until developing insects were fully sclerotized and darkened adults. We extracted adults from under the bark using a mallet and chisel and then confirmed species and sex (Lyon, 2012). Beetles were immediately flash frozen in liquid nitrogen or in most cases, held on moist filter paper at 2°C for 2 d to reduce gut contents before being flash frozen, and then stored at -80°C until DNA extraction. We sourced adult beetles from the Duffey Lake site in 2017 for 10x Chromium sequencing (male, SAMN15325870; female, SAMN15325942), in 2018 for male 10x Chromium and Dovetail Genomics Chicago (SAMN15325384) and Dovetail Genomics Hi-C (SAMN15325521) sequencing, and in 2019 for male Nanopore sequencing (SAMN26681013). We sourced adult beetles from the Prince George site in 2019 for genome size determination by flow cytometry. Beetles (adult and larvae) for ddRAD sequencing were collected from infested trees from 14 sites in North America (Table S1). Beetles were stored in 95% ethanol or RNAlater at -20°C until DNA extraction and purification. Voucher specimens from these locations have been deposited in the Spencer Entomological Collection of the Beaty Biodiversity Museum, University of British Columbia, Vancouver, BC, Canada (Supplementary File S1). Adult beetles for testing the PCR-based sex determination protocol were collected in 2021 from infested trees in southeastern British Columbia (50.21493° N, 114.97922° W, 1400 m elevation). The sex of adult beetles were determined by the authors morphologically (Lyon, 2012).

DNA extraction methods

Males for Nanopore sequencing (SAMN26681013)

We chose males for the initial assembly because they are the heterogametic sex (XY) and thus we would expect to sequence both sex chromosomes. Genomic DNA was extracted using the Blood and Cell Culture DNA Mini Kit with 20/G Tips (QIAGEN). Insects were homogenized individually in 500 µl of modified G2 buffer (QIAGEN User-Developed Protocol, 2001) using a 2 ml Dounce homogenizer with 20 passages of the tight (B) pestle. Insect homogenates were collected in 2 ml DNA LoBind tubes (Eppendorf) using wide-bore pipette tips. After homogenizing, remaining tissue in the Dounce homogenizer was washed with 500 µl of modified G2, which was added to the

insect homogenate. Each sample was split into two 1 ml fractions and supplemented with 20 µg/ml Ambion recombinant RNase A (Invitrogen) and 0.8 mg/ml Proteinase K (Invitrogen). Samples were incubated in a Thermomixer (Eppendorf) at 50°C and 900 rpm for 2 h. After incubation, samples were centrifuged at 15000 x *g* at 4°C for 20 min. Supernatants were carefully transferred to new tubes without disturbing the pellet. 20/G Genomic tips were equilibrated with 1 ml of QBT buffer. Samples were briefly vortexed before being loaded on Genomic-tip 1 ml at a time. Genomic-tips were then washed four times with GC buffer. DNA was eluted with 2 x 1 ml of QF buffer prewarmed to 50°C and then precipitated by adding 0.7x volumes of isopropanol. Samples were gently mixed by inversion 10-20 times before pelleting the genomic DNA by centrifuging at 15 000 x *g* for 30 minutes at room temperature (instead of 4°C in order to minimize salts and polysaccharides which could co-precipitate with DNA). Supernatants were carefully discarded, and DNA pellets were washed twice with 1 ml of 70% ethanol. Genomic DNA pellets were air-dried and then re-suspended in 100 µl of 10 mM Tris pH 8.0 and left to dissolve overnight at room temperature. DNA concentration was measured using a Qubit 2.0 with the dsDNA BR Assay Kit (Invitrogen). DNA from three individual males were combined to have sufficient DNA for Nanopore sequencing.

Males and females for 10x Chromium sequencing (SAMN15325870 and SAMN15325942)

We prepared high-molecular weight genomic DNA from individual whole insects using the protocol for single insect samples by 10x Genomics (2018). Briefly, we homogenized insects with a razor blade in a 50 µl drop of lysis buffer (600 µl of 10 mM Tris-HCl, 400 mM NaCl, and 100 mM EDTA, pH 8.0, 40 µl of 10% SDS, and 100 µl 20 mg/ml Proteinase K Solution) and then transferred it to a microcentrifuge tube with the remaining lysis buffer. After incubating for 16 h at 37°C, we separated the supernatant from the insect debris with two centrifugations at 2,000 x *g* for 2 min at 20°C. The supernatant was then gently mixed with 240 µl 5 M NaCl and cooled on ice before centrifuging at 1,100 x *g* for 15 min at 4°C. We then pipetted the supernatant with a wide-bore tip into a new 2 ml tube and added 1 ml of 100% isopropanol, gently inverting to mix until homogeneous. A glassy pellet of DNA could then be seen after centrifugation at 6,300 x *g* for 10 min at 4°C. The supernatant was discarded and the pellet was washed twice with 50 µl of ice-cold 70% ethanol, centrifuging at 6,300 x *g* for 5 min at 4°C with each wash. The remaining ethanol was allowed to evaporate for 5 min before 35 µl of TE Buffer (10 mM Tris-HCl, 1 mM EDTA, pH 8.0) was added and the pellet gently resuspended with occasionally inversions over 1 h at room temperature. Quality and quantity were assessed by NanoDrop ND-1000, Qubit 2.0 with the dsDNA BR Assay Kit (Invitrogen), and pulsed-field gel electrophoresis.

Males for 10x Chromium and Dovetail Genomics Chicago (SAMN15325384) sequencing

Dovetail Genomics extracted genomic DNA using the Blood and Cell Culture DNA Mini Kit, in a manner similar to that described above for the males for Nanopore sequencing,

except three males were pooled and they were homogenized using a drill-powered mortar and pestle.

Beetles for population genetics

Genomic DNA was extracted from whole individual larvae using the DNeasy Blood and Tissue Mini kit (QIAGEN) following manufacturer's instructions. For purification, 1/10 vol of 3M NaOAc (pH 5.2) and 2.5 x vol 95% ethanol was added to each sample and incubated overnight at 4°C. Samples were centrifuged at 14,000 x *g* for 30 min at 4°C. The supernatant was discarded, and the pellet was rinsed with 70% ethanol. Samples were centrifuged at 14,000 x *g* for 15 min at 4°C, the supernatant was aspirated, and the pellet was left to dry at room temperature. Each pellet was dissolved in 100 µl of 1X TE. DNA samples were quantified using a Qubit™ 3.0 Thermofisher and Qubit™ dsDNA Quantification Assay Kit (Thermo-Fisher) following manufacturer's instructions.

Genome sequencing library preparation and sequencing methods

Nanopore library preparation and sequencing:

Canada's Michael Smith Genome Sciences Centre (GSC) completed library construction and Oxford Nanopore Technologies (PromethION) sequencing. They prepared whole-genome PCR-free libraries from 2 µg genomic DNA, at a concentration of 42 ng/µL, following the ONT ligation-based library preparation kit (SQK-LSK110) implemented on a NIMBUS liquid handling robot (Hamilton). PromethION flow cells (exhibiting >5000 active pores within 5 d of construction to preserve the pore-targeting adapter) were loaded with 500 ng of recovered library (after ligation and bead purifications) and run for 72 h for maximal sequencing yield.

Chicago Hi-C library preparation and sequencing:

Dovetail Genomics prepared a Chicago library as described previously (Putnam et al., 2016). Briefly, ~500 ng of HMW gDNA (mean fragment length = 100 kb) was reconstituted into chromatin *in vitro* and fixed with formaldehyde. Fixed chromatin was digested with DpnII, the 5' overhangs filled in with biotinylated nucleotides, and then free blunt ends were ligated. After ligation, crosslinks were reversed and the DNA was purified from the protein. Purified DNA was treated to remove biotin that was not internal to ligated fragments. The DNA was then sheared to ~350 bp mean fragment size and sequencing libraries were generated using NEBNext Ultra enzymes and Illumina-compatible adapters. Biotin-containing fragments were isolated using streptavidin beads before PCR enrichment of each library. We sequenced the libraries on an Illumina HiSeq platform to produce 210 million 2x150 bp paired end reads, which provided 174-fold physical coverage of the genome (1-100 kb pairs).

Hi-C library preparation and sequencing:

Dovetail Genomics prepared a Hi-C library in a similar manner as described previously (Lieberman-Aiden et al., 2009). Chromatin from one insect (SAMN15325521) was fixed in place with formaldehyde in the nucleus and then extracted. The libraries were then

prepared from the fixed chromatin as described above. We sequenced the libraries on an Illumina HiSeq HiSeq X platform to produce 172 million 2x150 bp paired-end reads, which provided 143-fold physical coverage of the genome (10-10,000 kb pairs).

10x Library preparation and sequencing:

Prepared by Dovetail Genomics: Genomic DNA was adjusted to a concentration of 1.0 ng/μl and 1.25 ng of template gDNA was loaded on a Chromium Genome Chip. Whole genome sequencing libraries were prepared using Chromium Genome Library & Gel Bead Kit v.2 (10x Genomics, cat. 120258), Chromium Genome Chip Kit v.2 (10x Genomics, cat. 120257), Chromium i7 Multiplex Kit (10x Genomics, cat. 120262) and Chromium controller according to manufacturer's instructions with one modification. Specifically, gDNA was combined with Master Mix, a library of Genome Gel Beads, and partitioning oil to create Gel Bead-in-Emulsions (GEMs) on a Chromium Genome Chip. The GEMs were isothermally amplified with primers containing an Illumina Read 1 sequencing primer, a unique 16 bp 10x bar-code and a 6 bp random primer sequence, and bar-coded DNA fragments were recovered for Illumina library construction. The amount and fragment size of post-GEM DNA was quantified prior using a Bioanalyzer 2100 with an Agilent High sensitivity DNA kit (Agilent, cat. 5067-4626). Prior to Illumina library construction, the GEM amplification product was sheared on an E220 Focused Ultrasonicator (Covaris, Woburn, MA) to approximately 350 bp (55 sec at peak power = 175, duty factor = 10, and cycle/burst = 200). Then, the sheared GEMs were converted to a sequencing library following the 10X standard operating procedure. The library was quantified by qPCR with a Kapa Library Quant kit (Kapa Biosystems-Roche) and sequenced on [a partial lane (~350M reads/1.5Gb of genome)] of the NovaSeq6000 sequencer (Illumina, San Diego, CA) with paired-end 150 bp reads.

Prepared by GSC: A micro-fluidic partitioned library was created using the Chromium system from 10x Genomics (10x Genomics, Pleasanton, CA, USA). GEMs (Gel beads-in-EMulsion) were produced by combining DNA, Master Mix, and partitioning oil in the 10x Genomics Chromium Controller instrument with the micro-fluidic Genome Chip (PN-120216) (10x Genomics). The DNA in each GEM underwent isothermic amplification as a barcode was added to each fragment. Barcoded fragments then underwent Illumina library construction (as per the Chromium Genome Reagent Kits Version 2 User Guide (PN-120229)). The resulting libraries were assessed for quality using Agilent 2100 Bioanalyzer (Santa Clara, CA, USA) and DNA 1000 assays. The average insert size was 425 bp. The library was sequenced on an Illumina HiSeqX sequencer using the paired-end protocol to produce 660 million 150 base reads.

Protocol to determine sex of *Dendroctonus rufipennis* by PCR

A method for extracting gDNA and then PCR for scoring sex based on presence/absence of bands on an agarose gel.

Extraction of genomic DNA

Any appropriate method to extract genomic DNA from insects should work. Our method described here was developed to extract genomic DNA from samples that were destined for RNASeq, with RNA extraction performed using the RNeasy Mini Plant Kit (QIAGEN). The quantity of the material used for gDNA extraction had to be as small as possible. QIAGEN's protocol was modified to enable the collection of a small volume of homogenized sample prior to processing through the QiaShredder column and DNase treatment. This collected fraction of sample homogenate is the starting material for gDNA extraction.

In this modified QIAGEN protocol, 460 µl of RLT buffer was used instead of 450 µl. Samples were homogenized in Eppendorf Safe Lock 2 ml tubes with one 3 mm stainless steel ball bearing using a Mixer Mill Retsch MM200 Mixer Mill at 28 Hz for 1-2 min. After homogenisation, 15 µl of each insect sample was set aside in 2 ml microcentrifuge tubes and stored at -20°C until all RNA extractions were processed. The protocol below is used for the DNA extractions. Normally one stops at step 9.

Adapted from a *Medicago truncatula* gDNA MiniPrep protocol from Daniela Floß, Boyce Thompson Institute for Plant Research, Ithaca, New York (adapted from a method for Maize by Stephen L. Dellaporta, Jonathan Wood, James B. Hicks)

Set the water bath at 65°C for Step 3 and to solubilize the 20% SDS solution

1. Prepare the volume needed of Extraction Buffer by adding 34 µl of β-mercaptoethanol per 50 ml of buffer.

2. Add 750 µl of Extraction Buffer to each sample and vortex.

3. Add 50 µl of 20% sodium dodecyl sulfate (SDS) to each sample and mix by vortexing. Incubate the samples 65°C for 10 min.

4. Add 250 µl of 5 M potassium acetate to each sample. Shake the tubes vigorously. (NO VORTEXING!) Incubate the samples on ice for 20 min.

5. Centrifuge 18 000 x *g* for 20 min at room temperature. Transfer as much supernatant as possible, without transferring any solid, to a new 1.5 ml microcentrifuge tube.

6. Centrifuge 18 000 x *g* for 5 min at room temperature to pellet any transferred solid. Transfer supernatant to a new 1.5 ml microcentrifuge tube.

7. Add 500 µl of isopropanol per sample. Mix by inversion. Incubate the samples at -20°C for at least 30 min.

8. Centrifuge 18 000 x *g* for 20 min at room temperature. Gently pour off (or pipet) the supernatant and dry the DNA pellet. (*Do an additional quick spin and pipet out the leftover supernatant if needed*)

9. It is possible to stop the extraction here by resuspending DNA pellet in 25 to 50 µl of ddH_2O.

   If cleaner DNA is needed, resuspend the DNA pellet in 35 µl of ddH_2O *and continue to step 10.*

10. Add 3.75 µl of 3 M sodium acetate (NaOAc) pH 5.2 per sample.
    Add 25 µl of isopropanol per sample.
    Mix the samples by finger flick.

11. Pellet the DNA by centrifugation 18 000 x *g* for 1 min at room temperature.
    Discard the supernatant and wash the pellet with 1 ml of 70% ethanol.
    Centrifuge 18 000 x *g* for 1 min at room temperature.
    Discard the supernatant and dry the pellet.
    (*Do an additional quick spin to pipet out the leftover supernatant if needed*)

12. Dissolve the DNA in 30 to 50 µl of ddH_2O.

13. Optional: Before using the DNA for PCR, heat at 65°C for 10 min in the water bath. This is especially important if the DNA has been stored frozen before using.

<u>DNA Extraction Buffer</u>
100 mM Tris pH 8.0
50 mM EDTA pH 8.0
500 mM NaCl
**Add β-mercaptoethanol just before use: 34 µl per 50 ml of Extraction Buffer.

D. rufipennis sexing by PCR

Non-homologous regions between the X and Y chromosomes in the spruce beetle genome assembly were selected for primer design. Sex is scored by the presence and number of bands: one band for female (X), and two bands for male (X and Y), at the sizes shown below. The X amplicon acts as a PCR control; a failure to see an X band in

any sample indicates a failed reaction or extraction. Adapted from a method by Chiu *et al.* (2018) for *D. ponderosae*.

Primer used:
VLT230922-01-F (DrufX-F) TTCTAGGGCAGGGCATTTAC
VLT230922-02-R (DrufX-R) CGCACTCTTACCCTTACACTAC
VLT230922-05-F (DrufY-F) CTCTGTGGCTGCAAGTAGATATT
VLT230922-06-R (DrufY-R) CTCGATCGCAAGACGGATTTA
Amplicon sizes:
X: 621 bp
Y: 284 bp
Annealing temperature:
57°C
Elongation:
45 s
Cycles:
Usually, 30 cycles of amplification are enough.
For 1x reaction of PCR using Taq DNA Polymerase with Standard Taq Buffer (NEB, M0273)

gDNA template	1 µl (1 – 10 ng gDNA is normally sufficient)
dNTP mix (10 mM)	0.4 µl
Primer DrufX-F (10 µM)	0.4 µl
Primer DrufX-R (10 µM)	0.4 µl
Primer DrufY-F (10 µM)	0.4 µl
Primer DrufY-R (10 µM)	0.4 µl
10X Standard Taq Buffer	2 µl
50 mM $MgCl_2$*	0.8 µl (2 mM)
Taq	0.1 µl
H_2O	14.1 µl
Total	20 µl

*The concentration of $MgCl_2$ in 1X Standard Taq Buffer is 1.5 mM. This is not usually sufficient for a successful PCR using gDNA extracted from bark beetles.
Adding an extra 2 mM, for a final $MgCl_2$ concentration of 3.5 mM usually solved the problem. For recalcitrant samples, adjusting $MgCl_2$ and DNA template concentrations may be necessary (see: Lorenz, 2012).

Upon completion of the PCR, run samples on an agarose gel, observe the bands, and score sex as described above. A typical gel is shown below.

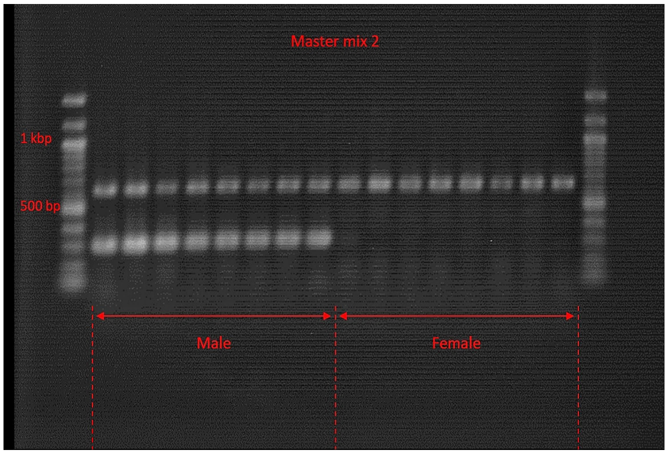
Master mix 2
1 kbp
500 bp
Male
Female

Annotation of cytochrome P450 and Carboxyl/cholinesterase (CCE) gene families

We manually curated the cytochrome P450 superfamily and the carboxyl/cholinesterases (CCEs) gene family using *Apollo* (Dunn et al., 2019) and preexisting *Dendroctonus* spp. protein and transcriptomic data, and the CCE-specific ESTHER (ESTerases and alpha/beta-Hydrolase Enzymes and Relatives) database (Chatonnet et al., 2023). We used *tBLASTn* and *BLASTp* searches of the annotated genome and the predicted proteins from the *BRAKER2* annotations with queries of previously identified P450s (Keeling et al., 2013) and carboxylesterases (Bernier, 2023) from *D. ponderosae*, and all ESTHER CCEs from *Tribolium castaneum* (trica) (downloaded 2025-02-20, Chatonnet et al., 2023). We aligned the P450 proteins with *MUSCLE* in *Geneious* software (v9.1.8, Dotmatics.com) using default parameters except for an *open gap score of -20* and a maximum reiteration of 16. Prior to alignment, we removed subcellular localization signals (signal peptide and mitochondrial localization sequence) identified by *DeepLoc-2.1* (Ødum et al., 2024). We trimmed the flanking sequences on both sides of the alignment, made manual adjustments to minimize gaps, and then ran a second pass in *MUSCLE* in *refine existing alignment* mode. We aligned the CCE proteins with *mafft* and the L-INS-i iterative alignment method (v7.526, Katoh & Standley, 2013). Subsequently, we prepared both phylogenies using *FastTree2* (v2.1.11, with gamma and wag flags, Price et al., 2010) and visualized with *FigTree* (v1.4.4, Rambaut, 2018).

Mitochondrial genome assembly and annotation

We used *minimap2* (v2.28, Li, 2021) to map the Nanopore reads to the published *D. rufipennis* mitochondrial genome (NC_063906, 16,937 bp). Due to excessively high coverage for accurate assembly, multiple random samplings of 2% of the mapped reads with sequence lengths between 5,000 and 15,000 bp were then assembled using *flye* (v2.9.6-b1802, Kolmogorov et al., 2019, 2020), yielding nearly identical circular scaffolds. We then polished a representative scaffold from one assembly with three rounds of *Pilon* as described above. We manually corrected any remaining errors (lingering indel errors) based on mapping 100% of the 5,000 and 15,000 bp Nanopore reads and Illumina reads (SRR12053743) with *minimap2* and *bwa mem*, respectively. Annotations were manually curated after prediction with *MITOS2* (v999, Donath et al., 2019) (on usegalaxy.org/, The Galaxy Community et al., 2024) and *MitoZ* annotate (v3.6, Meng et al., 2019).

Identifying co-sequenced organisms and nematode genome analyses

General

We used *PhylOligo* (v1.0, Mallet et al., 2017) and *hdbscan* (McInnes et al., 2017) with default parameters to cluster scaffolds of the gap-filled assembly longer than 1 kpb, 5 kbp, 10 kbp, and 20 kbp (Figure S19) based on oligonucleotide frequencies. We then used *BLASTn* and the NCBI nt Release 251 database to identify the taxonomic origin of each major cluster. A distinct small cluster of long scaffolds constituted the chromosome-level assembly of the co-sequenced nematode (labelled Big-scaffold nematode in Figure S19). A large cluster of shorter scaffolds constituted the incomplete

genomes of other nematodes (labelled Small-scaffold nematodes in Figure S19). Due to the multiple contaminants in this assembly, the *contalocate.R* function of *PhylOligo* could not adequately separate the scaffolds into beetle vs non-beetle groups nor bin the different contaminants. Instead, we examined the *BLASTn* and *BLASTx* results for each scaffold individually and manually binned them. Those identified as of insect origin were combined to represent the beetle assembly. After *Braker2* annotation of the spruce beetle assembly, we also used *BLASTp-fast* to confirm and correct any scaffold misidentifications. Finally, with the release of *FCS-GX* (v0.5.5-0-g0bda491, Astashyn et al., 2024) we reexamined the assembly and removed four additional scaffolds totaling 62,920 bp from the beetle assembly. The 15 *big-scaffold nematode* sequences were nearly identical to the 17 scaffolds that *FCS-GX* flagged as contaminants closest to *Bradynema listronoti*. The *small-scaffold nematodes* sequences were flagged by *FCS-GX* as contaminants closest to *Bursaphelenchus* and *Aphelenchoides* spp. We assessed whether the big-scaffold nematode sequences originated from one or more related species by mapping the Nanopore reads to the gap-filled assembly with *minimap2* (v2.28, Li, 2021) and determined SNP density with *bcftools* (v1.22, Danecek et al., 2021) after filtering SNPs for quality > 30 and read depth between 50 and 200. We then used methods as described for the spruce beetle to annotate the nuclear and mitochondrial genome of the co-sequenced nematode, except we used the *BUSCO* metazoa_odb12 lineage and the NCBI RefSeq Invertebrate Protein Release 231 for annotation with *BRAKER3* (v3.0.8, Barnett et al., 2011; Bruna et al., 2020, 2021; Buchfink et al., 2015; Gotoh, 2008; Hoff et al., 2016, 2019; Iwata & Gotoh, 2012; Li et al., 2009; Lomsadze et al., 2005, 2014; Stanke et al., 2006, 2008), and the nematoda_odb12 lineage for *BUSCO* analyses.

18S-based phylogeny

We used the 18S-NemaBase (Gattoni et al., 2023) to identify scaffolds containing nematode *18S* ribosomal RNA gene sequences. We then aligned the partial *18S* sequences from these scaffolds (three), with the *18S* sequences from Thylenchomorpha in the 18S-NemaBase, *18S* sequences from *Bursaphelenchus*, *Contortylenchus*, and *Ektaphelenchus* spp. recently added to NCBI, and the *18S* sequence from *Panagrolaimus davidi* (Panagrolaimomorpha) as the outgroup using *mafft* (L-INS-i iterative alignment method, maxiterate=16, v7.526, Katoh & Standley, 2013) after reducing sequence redundancy (sequence identity threshold 0.99) with *cd-hit* (v4.8.1, Fu et al., 2012). We prepared the phylogeny with *FastTreeMP* (*FastTree2* v2.1.10, with gamma flag, Price et al., 2010) and visualized it with *FigTree* (v1.4.4, Rambaut, 2018).

Species phylogenetic tree based on conserved proteins

We situated the unknown species within the phylum Nematoda with its genome assembly sequence data using a weighted, coalescent-based, species phylogenetic tree derived from multiple gene trees. We downloaded all currently available Nematoda reference genomes from NCBI (231 species, representing 105 genera, Supplementary File S5). We then identified the complete single copy orthologous sequences from each genome with *BUSCO* (v6.0.0 and the nematoda_odb12, 2025-07-01, n=596 database,

genome mode). We prepared separate protein alignments (557, each containing at least 192 species) for each *BUSCO* group that was complete and single copy in our unknown species using *mafft* (L-INS-i iterative alignment method, v7.526, Katoh & Standley, 2013) and *FastTreeMP* (*FastTree2* v2.1.10, with gamma and wag flags, Price et al., 2010). We then created a species phylogeny with *wASTRAL* (hybrid mode, *ASTER* v1.23.3.7, Zhang & Mirarab, 2022) and visualized it with *FigTree* (v1.4.4, Rambaut, 2018).

References for Supplementary Materials and Methods

10x Genomics. (2018). *Sample preparation demonstrated protocol: DNA extraction from single insects*. https://assets.ctfassets.net/an68im79xiti/3oGwQ5kl6UyCocGgmoWQie/768ae48be4f99b1f984e21e409e801fd/CG000145_SamplePrepDemonstratedProtocol_-DNAExtractionSingleInsects.pdf

Astashyn, A., Tvedte, E. S., Sweeney, D., Sapojnikov, V., Bouk, N., Joukov, V., Mozes, E., Strope, P. K., Sylla, P. M., Wagner, L., Bidwell, S. L., Brown, L. C., Clark, K., Davis, E. W., Smith-White, B., Hlavina, W., Pruitt, K. D., Schneider, V. A., & Murphy, T. D. (2024). Rapid and sensitive detection of genome contamination at scale with FCS-GX. *Genome Biology*, *25*(1), 60. https://doi.org/10.1186/s13059-024-03198-7

Barnett, D. W., Garrison, E. K., Quinlan, A. R., Stromberg, M. P., & Marth, G. T. (2011). BamTools: A C++ API and toolkit for analyzing and managing BAM files. *Bioinformatics*, *27*(12), 1691–1692. https://doi.org/10.1093/bioinformatics/btr174

Bernier, K. (2023). *Caractérisation fonctionnelle d'une carboxylestérase impliquée dans la libération d'une phéromone d'agrégation de Dendroctonus ponderosae* [M.Sc. Thesis, Université Laval]. https://hdl.handle.net/20.500.11794/125386

Bruna, T., Hoff, K. J., Lomsadze, A., Stanke, M., & Borodovsky, M. (2021). BRAKER2: Automatic eukaryotic genome annotation with GeneMark-EP+ and AUGUSTUS supported by a protein database. *NAR Genomics Bioinformatics*, *3*(1), lqaa108. https://doi.org/10.1093/nargab/lqaa108

Bruna, T., Lomsadze, A., & Borodovsky, M. (2020). GeneMark-EP+: Eukaryotic gene prediction with self-training in the space of genes and proteins. *NAR Genomics Bioinformatics*, *2*(2), lqaa026. https://doi.org/10.1093/nargab/lqaa026

Buchfink, B., Xie, C., & Huson, D. H. (2015). Fast and sensitive protein alignment using DIAMOND. *Nature Methods*, *12*(1), 59–60. https://doi.org/10.1038/nmeth.3176

Chatonnet, A., Perochon, M., Velluet, E., & Marchot, P. (2023). The ESTHER database on alpha/beta hydrolase fold proteins—An overview of recent developments. *Chemico-Biological Interactions*, *383*, 110671. https://doi.org/10.1016/j.cbi.2023.110671

Chiu, C. C., Keeling, C. I., & Bohlmann, J. (2018). Monoterpenyl esters in juvenile mountain pine beetle and sex-specific release of the aggregation pheromone *trans*-verbenol. *Proceedings of the National Academy of Science, USA*, *115*(14), 3652–3657. https://doi.org/10.1073/pnas.1722380115

Danecek, P., Bonfield, J. K., Liddle, J., Marshall, J., Ohan, V., Pollard, M. O., Whitwham, A., Keane, T., McCarthy, S. A., Davies, R. M., & Li, H. (2021). Twelve years of SAMtools and BCFtools. *GigaScience*, *10*(2), giab008. https://doi.org/10.1093/gigascience/giab008

Donath, A., Juhling, F., Al-Arab, M., Bernhart, S. H., Reinhardt, F., Stadler, P. F., Middendorf, M., & Bernt, M. (2019). Improved annotation of protein-coding genes boundaries in metazoan mitochondrial genomes. *Nucleic Acids Research*, *47*(20), 10543–10552. https://doi.org/10.1093/nar/gkz833

Dunn, N. A., Unni, D. R., Diesh, C., Munoz-Torres, M., Harris, N. L., Yao, E., Rasche, H., Holmes, I. H., Elsik, C. G., & Lewis, S. E. (2019). Apollo: Democratizing

genome annotation. *PLoS Computational Biology*, *15*(2), e1006790. https://doi.org/10.1371/journal.pcbi.1006790

Fu, L., Niu, B., Zhu, Z., Wu, S., & Li, W. (2012). CD-HIT: accelerated for clustering the next-generation sequencing data. *Bioinformatics*, *28*(23), 3150–3152. https://doi.org/10.1093/bioinformatics/bts565

Gattoni, K., Gendron, E. M. S., Sandoval-Ruiz, R., Borgemeier, A., McQueen, J. P., M. Shepherd, R., Slos, D., O. Powers, T., & L. Porazinska, D. (2023). 18S-NemaBase: Curated 18S rRNA database of nematode sequences. *Journal of Nematology*, *55*(1), 20230006. https://doi.org/10.2478/jofnem-2023-0006

Gotoh, O. (2008). A space-efficient and accurate method for mapping and aligning cDNA sequences onto genomic sequence. *Nucleic Acids Research*, *36*(8), 2630–2638. https://doi.org/10.1093/nar/gkn105

Hoff, K. J., Lange, S., Lomsadze, A., Borodovsky, M., & Stanke, M. (2016). BRAKER1: Unsupervised RNA-Seq-based genome annotation with GeneMark-ET and AUGUSTUS. *Bioinformatics*, *32*(5), 767–769. https://doi.org/10.1093/bioinformatics/btv661

Hoff, K. J., Lomsadze, A., Borodovsky, M., & Stanke, M. (2019). Whole-genome annotation with BRAKER. *Methods Molecular Biology*, *1962*, 65–95. https://doi.org/10.1007/978-1-4939-9173-0_5

Iwata, H., & Gotoh, O. (2012). Benchmarking spliced alignment programs including Spaln2, an extended version of Spaln that incorporates additional species-specific features. *Nucleic Acids Research*, *40*(20), e161. https://doi.org/10.1093/nar/gks708

Katoh, K., & Standley, D. M. (2013). MAFFT multiple sequence alignment software version 7: Improvements in performance and usability. *Molecular Biology and Evolution*, *30*(4), 772–780. https://doi.org/10.1093/molbev/mst010

Keeling, C. I., Yuen, M. M. S., Liao, N. Y., Docking, T. R., Chan, S. K., Taylor, G. A., Palmquist, D. L., Jackman, S. D., Nguyen, A., Li, M., Henderson, H., Janes, J. K., Zhao, Y., Pandoh, P., Moore, R., Sperling, F. A. H., Huber, D. P. W., Birol, I., Jones, S. J. M., & Bohlmann, J. (2013). Draft genome of the mountain pine beetle, Dendroctonus ponderosae Hopkins, a major forest pest. *Genome Biology*, *14*, R27.

Kolmogorov, M., Bickhart, D. M., Behsaz, B., Gurevich, A., Rayko, M., Shin, S. B., Kuhn, K., Yuan, J., Polevikov, E., Smith, T. P. L., & Pevzner, P. A. (2020). metaFlye: Scalable long-read metagenome assembly using repeat graphs. *Nature Methods*, *17*(11), 1103–1110. https://doi.org/10.1038/s41592-020-00971-x

Kolmogorov, M., Yuan, J., Lin, Y., & Pevzner, P. A. (2019). Assembly of long, error-prone reads using repeat graphs. *Nature Biotechnology*, *37*(5), 540–546. https://doi.org/10.1038/s41587-019-0072-8

Li, H. (2021). New strategies to improve minimap2 alignment accuracy. *Bioinformatics*, *37*(23), 4572–4574. https://doi.org/10.1093/bioinformatics/btab705

Li, H., Handsaker, B., Wysoker, A., Fennell, T., Ruan, J., Homer, N., Marth, G., Abecasis, G., & Durbin, R. (2009). The Sequence Alignment/Map format and SAMtools. *Bioinformatics*, *25*(16), 2078–2079. https://doi.org/10.1093/bioinformatics/btp352

Lieberman-Aiden, E., van Berkum, N. L., Williams, L., Imakaev, M., Ragoczy, T., Telling, A., Amit, I., Lajoie, B. R., Sabo, P. J., Dorschner, M. O., Sandstrom, R., Bernstein, B., Bender, M. A., Groudine, M., Gnirke, A., Stamatoyannopoulos, J., Mirny, L. A., Lander, E. S., & Dekker, J. (2009). Comprehensive mapping of long-range interactions reveals folding principles of the human genome. *Science*, *326*(5950), 289–293. https://doi.org/10.1126/science.1181369

Lomsadze, A., Burns, P. D., & Borodovsky, M. (2014). Integration of mapped RNA-Seq reads into automatic training of eukaryotic gene finding algorithm. *Nucleic Acids Research*, *42*(15), e119. https://doi.org/10.1093/nar/gku557

Lomsadze, A., Ter-Hovhannisyan, V., Chernoff, Y. O., & Borodovsky, M. (2005). Gene identification in novel eukaryotic genomes by self-training algorithm. *Nucleic Acids Research*, *33*(20), 6494–6506. https://doi.org/10.1093/nar/gki937

Lorenz, T. C. (2012). Polymerase Chain Reaction: Basic Protocol Plus Troubleshooting and Optimization Strategies. *Journal of Visualized Experiments*, (63), 3998. https://doi.org/10.3791/3998

Lyon, R. L. (2012). A Useful Secondary Sex Character in Dendroctonus Bark Beetles. *The Canadian Entomologist*, *90*(10), 582–584. https://doi.org/10.4039/Ent90582-10

Mallet, L., Bitard-Feildel, T., Cerutti, F., & Chiapello, H. (2017). PhylOligo: A package to identify contaminant or untargeted organism sequences in genome assemblies. *Bioinformatics*, *33*(20), 3283–3285. https://doi.org/10.1093/bioinformatics/btx396

McInnes, L., Healy, J., & Astels, S. (2017). hdbscan: Hierarchical density based clustering. *The Journal of Open Source Software*, *2*(11), 205. https://doi.org/10.21105/joss.00205

Meng, G., Li, Y., Yang, C., & Liu, S. (2019). MitoZ: A toolkit for animal mitochondrial genome assembly, annotation and visualization. *Nucleic Acids Research*, *47*(11), e63–e63. https://doi.org/10.1093/nar/gkz173

Ødum, M. T., Teufel, F., Thumuluri, V., Almagro Armenteros, J. J., Johansen, A. R., Winther, O., & Nielsen, H. (2024). DeepLoc 2.1: Multi-label membrane protein type prediction using protein language models. *Nucleic Acids Research*, *52*(W1), W215–W220. https://doi.org/10.1093/nar/gkae237

Price, M. N., Dehal, P. S., & Arkin, A. P. (2010). FastTree 2—Approximately maximum-likelihood trees for large alignments. *PLoS ONE*, *5*(3), e9490. https://doi.org/10.1371/journal.pone.0009490

Putnam, N. H., O'Connell, B. L., Stites, J. C., Rice, B. J., Blanchette, M., Calef, R., Troll, C. J., Fields, A., Hartley, P. D., Sugnet, C. W., Haussler, D., Rokhsar, D. S., & Green, R. E. (2016). Chromosome-scale shotgun assembly using an *in vitro* method for long-range linkage. *Genome Research*, *26*(3), 342–350. https://doi.org/10.1101/gr.193474.115

QIAGEN User-Developed Protocol. (2001). *Isolation of genomic DNA from mosquitoes or other insects using the QIAGEN® Genomic-tip*. https://www.qiagen.com/en-US/resources/download/Protocols/qg06-isolation-of-genomic-dna-from-mosquitoes-or-other-insects-using-the-qiagen-genomic-tip

Rambaut, A. (2018). *Figtree* [Computer software]. https://github.com/rambaut/figtree/releases

Stanke, M., Diekhans, M., Baertsch, R., & Haussler, D. (2008). Using native and syntenically mapped cDNA alignments to improve *de novo* gene finding. *Bioinformatics*, *24*(5), 637–644. https://doi.org/10.1093/bioinformatics/btn013

Stanke, M., Schoffmann, O., Morgenstern, B., & Waack, S. (2006). Gene prediction in eukaryotes with a generalized hidden Markov model that uses hints from external sources. *BMC Bioinformatics*, *7*, 62. https://doi.org/10.1186/1471-2105-7-62

The Galaxy Community, Abueg, L. A. L., Afgan, E., Allart, O., Awan, A. H., Bacon, W. A., Baker, D., Bassetti, M., Batut, B., Bernt, M., Blankenberg, D., Bombarely, A., Bretaudeau, A., Bromhead, C. J., Burke, M. L., Capon, P. K., Čech, M., Chavero-Díez, M., Chilton, J. M., … Zoabi, R. (2024). The Galaxy platform for accessible, reproducible, and collaborative data analyses: 2024 update. *Nucleic Acids Research*, *52*(W1), W83–W94. https://doi.org/10.1093/nar/gkae410

Zhang, C., & Mirarab, S. (2022). Weighting by gene tree uncertainty improves accuracy of quartet-based species trees. *Molecular Biology and Evolution*, *39*(12), msac215. https://doi.org/10.1093/molbev/msac215
